# Assessing access barriers to tuberculosis care with the tool to Estimate Patients' Costs: pilot results from two districts in Kenya

**DOI:** 10.1186/1471-2458-11-43

**Published:** 2011-01-18

**Authors:** Verena Mauch, Naomi Woods, Beatrice Kirubi, Hillary Kipruto, Joseph Sitienei, Eveline Klinkenberg

**Affiliations:** 1 KNCV Tuberculosis Foundation, Den Haag, The Netherlands; 2 Merlin, Goma, DR Congo; 3 Gertrude's Children's Hospital, Nairobi, Kenya; 4 Ministry of Public Health and Sanitation Kenya, Division of Leprosy, Tuberculosis and Lung Disease, Nairobi, Kenya; 5 World Health Organization Kenya, Nairobi, Kenya; 6 Department of Clinical Epidemiology, Biostatistics and Bioinformatics, Center for Infection and Immunity Amsterdam (CINIMA), Academic Medical Center, University of Amsterdam, Amsterdam, The Netherlands

## Abstract

**Background:**

The poor face geographical, socio-cultural and health system barriers in accessing tuberculosis care. These may cause delays to timely diagnosis and treatment resulting in more advanced disease and continued transmission of TB. By addressing barriers and reasons for delay, costs incurred by TB patients can be effectively reduced. A Tool to Estimate Patients' Costs has been developed. It can assist TB control programs in assessing such barriers. This study presents the Tool and results of its pilot in Kenya.

**Methods:**

The Tool was adapted to the local setting, translated into Kiswahili and pretested. Nine public health facilities in two districts in Eastern Province were purposively sampled. Responses gathered from TB patients above 15 years of age with at least one month of treatment completed and signed informed consent were double entered and analyzed. Follow-up interviews with key informants on district and national level were conducted to assess the impact of the pilot and to explore potential interventions.

**Results:**

A total of 208 patients were interviewed in September 2008. TB patients in both districts have a substantial burden of direct (out of pocket; USD 55.8) and indirect (opportunity; USD 294.2) costs due to TB. Inability to work is a major cause of increased poverty. Results confirm a 'medical poverty trap' situation in the two districts: expenditures increased while incomes decreased. Subsequently, TB treatment services were decentralized to fifteen more facilities and other health programs were approached for nutritional support of TB patients and sputum sample transport. On the national level, a TB and poverty sub-committee was convened to develop a comprehensive pro-poor approach.

**Conclusions:**

The Tool to Estimate Patients' Costs proved to be a valuable instrument to assess the costs incurred by TB patients, socioeconomic situations, health-seeking behavior patterns, concurrent illnesses such as HIV, and social and gender-related impacts. The Tool helps to identify and tackle bottlenecks in access to TB care, especially for the poor. Reducing delays in diagnosis, decentralization of services, fully integrated TB/HIV care and expansion of health insurance coverage would alleviate patients' economic constraints due to TB.

## Background

The association between tuberculosis (TB) and poverty has been well established [1-6]. The economically vulnerable are more likely to be exposed to conditions that predispose to infection with mycobacterium tuberculosis [3,7-11] and that propagate progression to disease [5,11,12].

The poor face geographical, economic and health system barriers to accessing care [5,13] which cause delays in seeking healthcare [14,15] resulting in more advanced disease and continued transmission of TB in the community. TB affects the most economically-productive age, posing a significant economic burden on affected households [3,16,17].

The three main types of economic constraints that TB patients face are 1) charges for health services, 2) costs for transport, accommodation and nutrition and 3) lost income, productivity and time [5,18-21]. Direct (out-of-pocket) costs for public or private services and indirect (opportunity) costs can trigger a spiral into (deeper) poverty for many families. This situation has been termed 'the medical poverty trap' [22].

By addressing economic barriers and reasons for delay to timely diagnosis and treatment, costs incurred by TB patients can be effectively reduced. A number of studies on patient costs have been published [1,13-16,18,20,21,23,24], however there was a need to address some of the methodological bottlenecks to assess barriers identified in these studies and make the Tool (including the questionnaire, guidelines, data entry template) available for national TB programs and other organizations working with TB. A consortium of partners (KNCV Tuberculosis Foundation, Japan Anti-Tuberculosis Association, World Health Organization Stop TB department and members of the Stop TB Partnership's TB & Poverty Subgroup) therefore developed a Tool to assist TB control programs in assessing such barriers, funded by the Tuberculosis Control Assistance Program (TB CAP/USAID). It can be used worldwide and is not limited to research organizations. The objective is to establish an evidence base upon which interventions can be designed.

A literature review on studies dealing with patients' costs and methodologies employed was conducted to determine at what stage patients are likely to incur which kinds of costs. The findings of the review formed the basis and context for the development of the Tool. The generic Tool is designed to assess direct (out of pocket) and indirect (opportunity) costs incurred by TB patients at two distinct phases: 1) before and during diagnosis and 2) during treatment. The Tool also includes questions on TB patient information; previous TB treatment episodes; health-seeking behavior and delays; costs to the guardian/treatment supporter of the patient; health facility visit costs; social impact of the disease on the family including children; and the impact of TB on food expenditures and the welfare of the household.

The Tool contains an introduction, a literature review on patient cost studies, a generic questionnaire to be adapted to local circumstances, a brief review of socioeconomic indicators, a list of indicators to be measured by the Tool, guidelines on the adaptation of the questionnaire to local circumstances, methods, sampling and training of interviewers, on interpretation of results generated by the questionnaire, and on possible interventions. An Epi Info template for data entry and an Excel template to summarize results have been added to facilitate data analysis. The Tool can be downloaded for free on the websites of the Tuberculosis Coalition for Technical Assistance (TBCTA; http://www.tbcta.org/Library/) and Stop TB Partnership TB & Poverty Subgroup (http://www.stoptb.org/wg/dots_expansion/tbandpoverty/spotlight.asp), see additional file 1.

**Figure 1:**
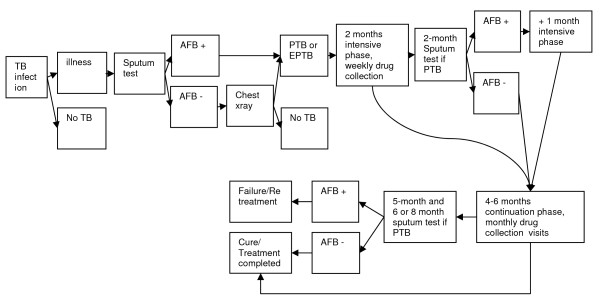
**Diagnosis and Treatment Algorithm**. In 2008/2009, the Kenyan TB program has changed its treatment regimen from an eight-month to a six-month regimen. Retreatment cases receive an eight-month regimen. Abbreviations: AFB: Acid-Fast Bacili; PTB: Pulmonary Tuberculosis; EPTB: Extra-Pulmonary Tuberculosis

The Tool was piloted in Kenya where TB diagnosis (sputum smear microscopy) and treatment at public health facilities are free of charge. The Kenyan national tuberculosis control program (Division of TB, Leprosy and Lung Disease DLTLD) follows the internationally-recommended DOTS approach, promoted by WHO since 2006 [25] with sputum smear microscopy as primary diagnostic tool for TB. Under DOTS, TB patients require a treatment supporter to ensure and document that the treatment has been followed continuously. The procedure followed by TB patients is summarized in figure 1. From a patient perspective, figure 1 shows a simplified process; between illness and diagnosis there are often many different care-seeking episodes. This paper presents the results from this pilot.

## Methods

### Study sites

Kitui North and Mutomo districts, both in Eastern Province, were purposively selected in consultation with DLTLD, considering proximity to Nairobi and level of socioeconomic development [26]. While 75% of incomes stem from agriculture, this area is prone to persistent droughts and crop failures resulting in food and income insecurity [27].

Nine out of 41 health facilities offering TB services in the pilot districts were purposively selected as pilot sites based on caseload and accessibility (table 1); seven were public health facilities and two were faith-based clinics. Private facilities charging fees for service were not selected with the focus of the pilot on the public sector. The target enrolment was 200 patients for a sample size sufficient to accurately evaluate the Tool and ensure sufficient power of the analysis. Interview quotas based on currently registered cases per facility as a proportion of district caseloads were assigned to each of the nine facilities.

**Table 1:** Information on pilot districts, 2008

	Kenya	Pilot District Kitui North	Pilot District Mutomo
Estimated population	38,277,856	473,241	291,576

Annual per capita income 2005 (KSH) [26]	35,480	10,699	n/a

Adult Literacy Rate, 2005 (%) [26]	68.7	69.9	n/a

TB cases (all types) notified in 2008	110,251	1,432	408

TB case notification rate/100.000 population	338	303	140

Estimated HIV prevalence in general population (%)	7.1 [36]	14 [27]	14 [27]

Number of public health facilities	6,696 [37]	74	17

TB treatment centers	2,228	41	12

TB treatment centers/100.000 population	6	9	4

(all data except where otherwise indicated [38])

### Study population

Every patient or treatment supporter representing a patient (guardian) at the selected health facility who fulfilled the in- and exclusion criteria as listed below was eligible and asked to participate. Interviews were conducted during TB clinic days.

#### Inclusion criteria

Patients (or guardians who came in place of patients) who:

- Were new or re-treatment patients

- Had received at least (≥) 1 month of treatment for TB (for their current diagnosis)

- Were at least (≥) 15 years of age

#### Exclusion criteria

- Currently hospitalized patients

- Patients under (<) the age of 15 years

All eligible patients were approached to enroll in the study. All patients who accepted to sign an Informed Consent form after being briefed on their rights as interviewees were enrolled in the study.

### Development of the questionnaire

The generic questionnaire of the Tool was adapted to the local setting in consultation with central program and local district staff. The questionnaire was pretested at one of the sites where four patients were interviewed, and adjustments were made as appropriate. The questionnaire was translated into Kiswahili and back translated into English to ensure accuracy of the translation. During extensive discussion with central and district staff, a decision was reached to ask patients about their HIV status. Six interviewers were selected and trained. All interviewers were Kiswahili speakers, and four were native Kamba speakers, the dominant local language. Interviews were conducted primarily in Kiswahili, with Kamba in prompting questions. The research protocol and standard operating procedure manual were approved by the Scientific Steering Committee of the Kenya Medical Research Institute (KEMRI).

### Data entry and analysis

Responses gathered from patients were entered into Epi Info 6.0, using double entry to enhance data quality. Consistency and range checks were used to ensure completeness of data.

For the pilot in Kenya, direct costs incurred during TB treatment were calculated per drug collection visit. Direct costs are quantifiable, out-of-pocket costs associated with TB: health care costs such as tests, administrative charges and medicines taken for TB symptoms prior to receiving a TB diagnosis; transport costs to and from health facilities; and associated food and accommodation costs. Any reimbursements received by patients through insurance were deducted. Costs were noted in Kenyan Shillings (KSH). The exchange rate was 65 KSH to one U.S. dollar.

Indirect costs were calculated as income lost due to TB. For income lost prior to treatment, the time off work was multiplied by the median reported individual income prior to the onset of TB. For income lost during treatment, the time off work was multiplied by the median reported individual income since the onset of TB.

Data was analyzed using Epi Info 6.0, Excel 2003 and STATA 9.0 (STATA, Statacorp, Texas, USA). The Shapiro-Wilk test was used to test for normality. Due to lack of normality in the quantitative data, non-parametric tests were used in the analysis. Wilcoxon matched pairs signed ranks test was used when it was not appropriate to use the paired comparison t-test. The Kruskal-Wallis test was used to test the equality of independent variables with two or more levels and an ordinal dependent variable. In the analysis of categorical variables, Fisher's exact test was used to test for association where the cell count was less than 5. Unless otherwise specified, median values were used as a measure of central tendency while inter-quartile range (IQR) was preferred to range in order to avoid extreme values. The level of statistical significance, according to the calculated sample size, was p = 0.05 with a confidence level of 95%.

Due to known unreliability of income data [18,28-33], household food expenditures and assets, partly based on the Kenya Demographic and Health Survey 2003 [34], were analyzed as proxies for income in addition to self-reported individual and household income. All respondents were assigned to five income groups to reflect socioeconomic strata in the study districts. The groups were determined jointly by study investigators and district-level TB program staff.

Six months after the pilot, follow-up interviews with key informants in the districts and at national level were conducted to assess the impact of the pilot and to explore potential interventions based on the findings.

## Results

A total of 208 patients were interviewed. No patients or guardians refused or stopped the interview.

**Figure 2:**
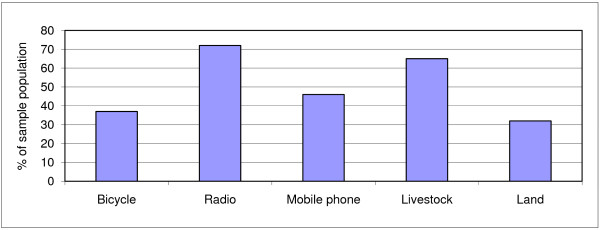
**Household asset ownership at time of interview**.

The majority of interviews (n = 188) took place in Kitui North, with 20 interviews in Mutomo. Nineteen guardians/treatment supporters were interviewed (9%). Differences in responses between guardians and patients were not observed. Tables 2 and 3 and figure 2 provide an overview on socioeconomic and TB-related information for the sample population. 59% of the study population was male with the majority between 26 and 40 years of age. 90% were new TB cases. 51% of primary household income earners had only primary education.

**Table 2:** Sample population characteristics

**Gender **(unknown: n = 2)	% (n)
Male	59 (121)

Female	41 (85)

**Age **(unknown: n = 2)	**% (n)**

≤ 25	21 (43)

26 - 40	51 (105)

>40	28 (58)

**Household size **(unknown: n = 2)	**% (n)**

< 3	10 (21)

3 to 5	44 (91)

> 5	46 (94)

**TB Type **(unknown: n = 2)	**% (n)**

PTB SS+	53 (109)

PTB SS-	39 (80)

EPTB	8 (17)

**TB treatment category; HIV**	**% (n)**

New cases	90 (187)

Retreatment cases	10 (21)

HIV positive	33 (69)


**Table 3:** Socioeconomic information of sample population

**Education of primary household income earner **(unknown: n = 2)	% (n)
Primary school	51 (105)

Secondary school	32 (66)

Not attended	10 (21)

High school certificate	6 (12)

Adult learning	1(2)

**Occupation **(unknown: n = 2)	**% (n)**

Small business	26 (54)

Casual labour	21 (43)

Housework	21 (43)

Subsistence farming	12 (25)

Civil servant	6 (12)

Transport, Student, Retired, Other	14 (29)

**Drinking water source**	**% (n)**

Lake, pond, dam, river	46 (96)

Public well	6 (12)

Private well, bore hole	15 (32)

Piped water, bottled water	33 (68)

**Type of toilet facility available**	**% (n)**

No facility, bush, field	5 (11)

Shared pit toilet, latrine	60 (124)

Own pit toilet, latrine	33 (68)

Flush toilet	2 (5)

**Type of housing**	**% (n)**

Dirt floor, thatched roof	12 (26)

Dirt floor, metal roof	50 (104)

Concrete floor	38 (78)

### Health seeking behaviour

Seventy-five percent of all patients reported accessing a public health facility first. The remaining 25% visited a private clinic or laboratory, and cited waiting times (51%) and distance to the facility (30%) as reasons behind their choice of facility. Only one patient visited a tradtional healer. The median delay between the onset of symptoms and seeking a diagnosis at a public facility was 2 months (IQR 1-3 months).

### Pre-diagnosis/diagnosis costs

Median direct costs incurred from the onset of TB symptoms to diagnosis at a public health facility were 860 KSH (13 USD) per patient. This includes costs related to visits to public, private or informal providers. A small number of patients had been hospitalised before their current TB diagnosis was made (n = 12), incurring disproportionately high costs: 2,177 KSH (hospitalized, median) versus 832 KSH (not hospitalized, median).

Patients required a median of three health facility visits, taking a median of 12 hours before receiving a diagnosis. In the pre-diagnosis phase travel to and from health facilities and costs for diagnostic tests accounted for most direct costs (38% and 37% of pre-diagnosis direct costs respectively). Additional costs were food purchased (lunch for health facility visits, vitamins; 19%) and administrative charges at health facilities (6%).

Pre-diagnosis/diagnosis costs did not vary significantly by age, gender or income level. However, patients with extra-pulmonary TB (EPTB) experienced significantly higher costs during the pre-diagnosis/diagnosis phase mainly due to additional costs of xrays, reporting a median of 1,450 KSH compared to a median of 860 KSH for patients of all other TB types (p = 0.0175).

### Treatment costs

Patients had completed a median of 4 out of an 8 month treatment regimen at the time of the interview. Costs due to DOT visits were not incurred, as all patients interviewed received family DOT at home.

Direct costs during treatment consisted of travel costs to collect drugs or for a follow-up sputum test (54%), food (45%), accommodation, other tests, other drugs and administration costs (together < 1%). Treatment costs did not differ significantly by age, gender, income level or TB type. The median total treatment cost was 105 KSH (1.6 USD; IQR 39-189 KSH) for each visit lasting a median of 3 hours each (IQR 2-4.5).

### Coping costs

Most patients reported having to borrow money (57%) and/or selling assets (52% of which 90% was livestock) to cover the costs incurred. Patient income did not determine whether or not they borrowed money. However, there was a relationship between patient income and asset disposal (p = 0.03; χ^2 ^= 10.53 with 4 d.f.), with middle-income patients selling significantly more than low- or high-income patients. Those who sold assets gained significantly less than the market price estimated by the patient (p = 0.000).

### Indirect costs

Indirect costs for the entire duration of illness (19,123 KSH; 294 USD) constituted 85% of total costs. For the pre-diagnosis phase, this was calculated using patients' median monthly income before the illness (4,250 KSH), multiplied by the median amount of time they were out of work as a direct result of their illness (4.5 months, n = 174). For the treatment phase, median monthly patient income after the onset of TB (0 KSH implying that patients have not resumed work) was multiplied by the amount of time required for TB treatment over 14 drug collection visits (42 hours).

### Total costs and change of income due to TB

**Figure 3:**
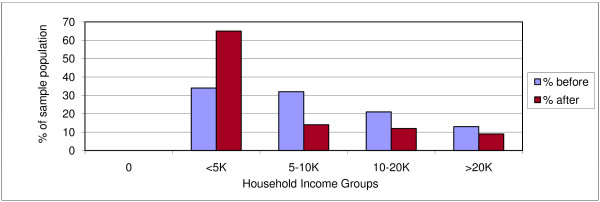
**Household monthly income groups before and after onset of TB**. Reported quintiles are those of the study population.

**Figure 4:**
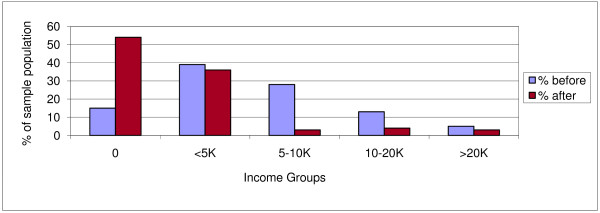
**Individual monthly income groups before and after onset of TB**.

Table 4 summarizes all incurred costs. The median total of direct and indirect costs was 22,753 KSH (350 USD). This was equivalent to 45% of median annual individual incomes (50,960 KSH; 784 USD) and 27% of median annual household incomes before TB illness (84,260 KSH; 1,296 USD). Both household and individual incomes dropped due to TB (figures 3, 4). After the onset of TB, total costs increased by 20%, to 47% of median household incomes. Food and healthcare expenditures increased from 46% to 223% of median monthly household income.

**Figure 5:**
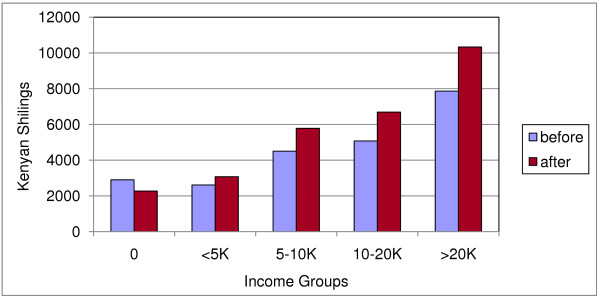
**Monthly household food expenditures by income group**.

Figure 5 shows the lowest income group reduced expenditures on food items due to TB while higher income groups tend to increase food expenditures due to TB. Adequate nutrition is important during treatment to make up for weight previously lost due to the illness. In addition, anti-TB drugs can cause nausea and are more easily digested after food intake.

**Table 4:** Total Costs and Summary

Median Direct Costs	KSH (IQR)	USD	% of median annual individual income (pre-TB)
Pre-Diagnosis/Diagnosis Costs	860 (500-1,670)	13.2 (8-26)	1.7

Treatment Costs (cost/visit × 14 visits)	1,470 (560-2,660)	22.6 (9-41)	2.9

Coping Costs	1,300 (800-2,500)	20 (12-38)	2.6

***Sub-Total***	***3,630***	***55.8***	***7.1***

**Median Indirect Costs**			

Foregone income before diagnosis (4.5 months of work lost × initial monthly income)	19,123 (6,750-40,500)	294.2	37.5

Foregone income during treatment: drug collection time × current income. (Total drug collection time = hours/visit × 14 visits)	42 hours × zero income = 0		

***Sub-Total***	***19,123***	***294.2***	***37.5***

**Median Total Costs**			

***Direct + Indirect Costs***	***22,753***	***350***	***44.6***

### HIV

33% of all patients interviewed reported to be HIV+. Two percent declined to answer the question, 53% confirmed they were HIV-, 4% had not been tested and 8% were unsure. Compared to HIV- patients, HIV+ patients experienced 16% higher direct costs during the pre-diagnosis/diagnosis phase and 48% higher costs during treatment, associated with collection of anti-retroviral drugs, however not statistically significant.

### Gender

**Figure 6:**
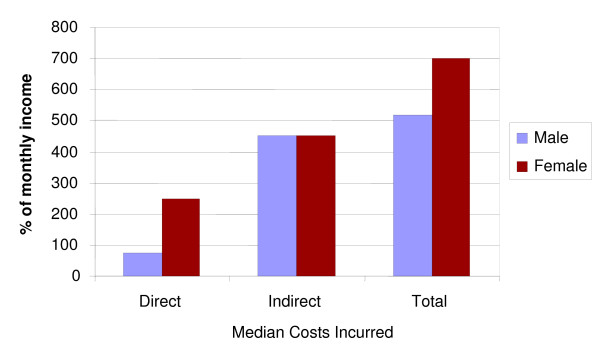
**Total direct and indirect costs by gender as% of monthly individual income**.

Men and women did not report statistically significant differences in costs. However, women were more likely to have low incomes and thus devoted a higher percentage of their income to TB-related costs (figure 6). Reported patient delay did not differ significantly between men and women.

### Productivity and social impact of TB

Patients were asked to estimate the number of hours they worked per week before and after the onset of TB. Of the 208 respondents, 85% reported a decrease as a result of TB illness. 15% of those who worked fewer hours had a family member take over their work, in 5% of cases this was a child. However, no child stopped school to undertake this work. Of all respondents, 9% had children that completed school and worked to raise money during the TB illness. 11% of respondents employed someone to do the housework. A house help earned a median of 2,000 KSH per month, which can be used to assign a value to unpaid household work. 28% of respondents reported disrupted social life due to TB, affecting sexual life (n = 93); job loss (n = 20); divorce (n = 5) and separation from spouse (n = 10).

### Insurance

Twelve percent of patients reported having some form of health insurance, with only one patient having inpatient benefits. Higher-income patients were more likely to have health insurance (p = 0.005). However only in one case insurance covered outpatient costs.

### Results of follow-up interviews

Six months after the presentation and discussion of the Tool's findings with program staff at national and district level, follow-up interviews were conducted with key informants (Kenyan TB experts and local staff involved in the initial study). The objectives of these interviews were to assess the impact of the pilot, the usefulness of study findings and to explore the feasibility of recommendations. Decentralization of treatment services and HIV service integration were seen to be of greatest benefit in tackling TB-related direct costs.

## Discussion

### Key findings

The implementation of the Tool to Estimate Patients' Costs showed that TB patients in Kitui North and Mutomo have a substantial burden of direct and indirect costs due to TB. The majority of costs are indirect (38% of annual individual income before the disease). Inability to work is a major cause of increased poverty and contributes to worsening of individual economic situations. The proportion of patients not earning a regular income increased from 15% to 54% after the onset of disease. Individual incomes dropped to zero after onset of TB and household incomes dropped by 43%. Although less than indirect costs, direct costs are still considerable (7% of individual annual income) with transport as the biggest cost item. Results confirm a 'medical poverty trap' [22] situation in the two districts: expenditures increased while incomes decreased.

### Usefulness of the Tool

The information generated by the Tool can be used as an evidence base for subsequent interventions to alleviate the financial burden of TB patients. Reducing delays in diagnosis, decentralization of services, integration of TB/HIV care and inclusion of TB in- and outpatient services in health insurance schemes while increasing insurance coverage could alleviate economic constraints due to TB [35]. Recommendations that were based on the findings of the pilot and the follow up interviews included: further decentralization of TB care, realignment of the currently used communication strategy to stimulate TB suspects to seek care early, mobile clinics, stronger TB/HIV program coordination to allow for integrated TB/HIV care, further usage of the Tool in other areas.

Following the study, fifteen more facilities in the pilot districts were equipped as TB treatment centres. The district TB and Leprosy coordinators approached the World Food Program to discuss nutritional support of TB patients and local partners for sputum sample transport to distant diagnostic centres to reduce patients' transport costs and time spent on the road. On the national level, a TB and poverty sub-committee was convened to develop a comprehensive pro-poor approach within the routine TB program.

### Comparability of the Tool

TB costing studies done elsewhere in East Africa and Asia show similar results (Table 5) in respect to direct costs (Malawi) and indirect costs (India). Due to study design differences, exact costs comparisons cannot be made, the results are nonetheless informative.

**Table 5:** TB Patient Cost Study Comparison

Study	Costs Incurred (% of household annual income)			Lost Work (months)
	Direct Costs	Indirect Costs	Total Costs	
India (rural/urban) [17]	13	26	40	2.5

Zambia (rural/urban) [21]	8.3	4.8	13.1	n/a

Thailand (rural/urban) [16]	8.6	2.3	10.9	2.7

China (rural) [13]	n/a	n/a	45	n/a

Kitui (rural/urban)	4.3	22.7	27	4.5

	**Costs Incurred** **(% of individual annual income)**			

Malawi (urban) [18]	5	6.3	11.5	

Kitui (rural/urban)	7.1	37.7	44.8	

### Study limitations

Estimating monetary incomes was difficult for those without regular salaries. Disaggregated costing questions about everyday amounts would be more graspable. Asking the same questions at different times of the agricultural year might yield different responses and it is difficult to ensure that patients are reporting incomes, not turnover from business. Furthermore, assigning a monetary value to unpaid housework and health insurance were difficult concepts to communicate. Patient recall declined noticeably after four months on TB treatment and time-sequence prompts were useful. A weighted asset index was not made as it was beyond the purpose of this study. Patients' mobility was omitted, hence migration patterns of TB patients were not assessed. Questions relating gender and equitable access were difficult to convey. More gender sensitization in interviewer trainings may have been necessary. Reporting expenditures for food as done in this study is not equal to food consumption. Therefore reduction in food expenditure does not necessarily imply a reduction in food consumption as households may produce their own food or may receive food from others when in need. Including local program staff as interviewers gave them an opportunity to speak with patients in-depth and to conceptualize strengths and challenges of the TB program. However, it is possible that the presence of staff influenced the responses of patients.

In Mutomo only 20 patients could be interviewed. This district was therefore relatively undersampled, because 22% (n = 44) were targeted based on case numbers. Due to the small sample, an analysis for sensitivity between the districts could not be done. However, significant differences between Mutomo and Kitui North are not expected as they used to be one district until 2008. Thus, a bias of study results is not presumed.

Sensitivity of income data was not analyzed in this study. When comparing annual per capita income of the Kenya Human Development Report [26] in 2005 (Kenya 35,480 KSH; Kitui North 10,699 KSH; 76 KSH per USD) with our finding of median annual individual income of 50,960 KSH in 2008 (65 KSH per USD), it is likely that self-reported income was over-estimated in this study. If we assume an over-estimate of 43%, median annual income before TB would be 523 USD instead of 748 USD; and median annual household income 906 USD instead of 1296 USD. Total costs would constitute 67% of annual individual income and 39% of annual household income which is 22% and 12% more than what our findings indicate. Future studies and implementers of this Tool are therefore advised to compare income results to recent national demographic and health surveys, data from the national statistics office or Human Development Reports to put results into context. In addition, food consumption and asset indices are recommended as proxies for income. Despite challenges in assessing income, the results of this study nevertheless show a remarkable impact of TB on the welfare of households and individuals.

## Conclusion

The Tool to Estimate Patients' Costs proved to be a valuable instrument to assess patients' costs, the socioeconomic situation of the patients, health-seeking behavior, concurrent illnesses such as HIV, as well as social and gender-related impacts. In addition, the Tool helps to assess the economic impact of TB on individual and household welfare and to identify bottlenecks in access to TB care. The mere usage of the Tool can already have an impact by involving staff who take responsibility in improving the situation for TB suspects and patients. Challenges such as recall bias and gender-related sensitivities are difficult to address; each cultural setting will need to find its best-suited approach. The improved Tool has been subsequently implemented in the Dominican Republic, Ghana and Viet Nam. Establishing evidence on patients' costs in different parts of the world will be instrumental in advancing the calls for free TB diagnosis, integration of TB/HIV services and comprehensive health and disability insurance for the poor.

## List of abbreviations used

AFB: Acid-Fast Bacilli; DLTLD: Division of Leprosy, Tuberculosis and Lung Disease; DOT(S): Directly Observed Treatment (Short-Course); EPTB: Extra-Pulmonary Tuberculosis; HIV: Human Immune Deficiency Virus; IQR: Inter-quartile Range; JATA: Japan Anti-Tuberculosis Association; KEMRI: Kenya Medical Research Institute; KNCV: Royal Netherlands TB Association; KSH: Kenyan Shillings; PTB: Pulmonary Tuberculosis; TB: Tuberculosis; TB CAP: Tuberculosis Control Assistance Program; TBCTA: Tuberculosis Coalition for Technical Assistance; WHO: World Health Organization

## Competing interests

No conflict of interest declared. The development and pilot of the Tool and this article are made possible by the generous support of the American people through the United States Agency for International Development (USAID), The Global Health Bureau, Office of Health, Infectious Disease and Nutrition (HIDN), that has supported financially through TB CAP under the terms of Agreement No.GHS-A-00-05-00019-00. The contents of this article are the responsibility of TB CAP and do not necessarily reflect the views of USAID or the United States Government.

## Authors' contributions

VM conceived, designed and coordinated the study, drafted the manuscript and prepared the final version. NW participated in the conception and design of the study, analysed and interpreted the data, drafted the manuscript and reviewed it critically for substantial intellectual content. BK participated in the conception and design of the study, the analysis and interpretation of data and the drafting of the manuscript and critically reviewed it for substantial intellectual content. HK participated in the conception and design of the study, supported data analysis and interpretation of data, and critically reviewed the manuscript for substantial intellectual content. JS participated the conception and design of the study and critically reviewed the manuscript for substantial intellectual content. EK participated in the conception and design of the study and its coordination and critically reviewed the manuscript for substantial intellectual content. All authors read and approved the final manuscript.

## Authors' information

At the time of the study Naomi Woods was graduate student of public policy at the Hertie School of Governance in Berlin, Germany.

## Pre-publication history

The pre-publication history for this paper can be accessed here:

http://www.biomedcentral.com/1471-2458/11/43/prepub

## Supplementary Material

Additional file 1 **Tool to Estimate Patients' Costs**. The complete Tool that is described in the manuscript. Click here for file
