# Supplementary material for: Assessing access barriers to tuberculosis care with the tool to Estimate Patients' Costs: pilot results from two districts in Kenya
Source: BMC Public Health. 2011 Jan 18;11:43. doi: 10.1186/1471-2458-11-43 (PMC3033813; doi:10.1186/1471-2458-11-43)
Supplement: Additional file 1 — Tool to Estimate Patients' Costs. The complete Tool that is described in the manuscript. [file 1471-2458-11-43-S1.PDF]

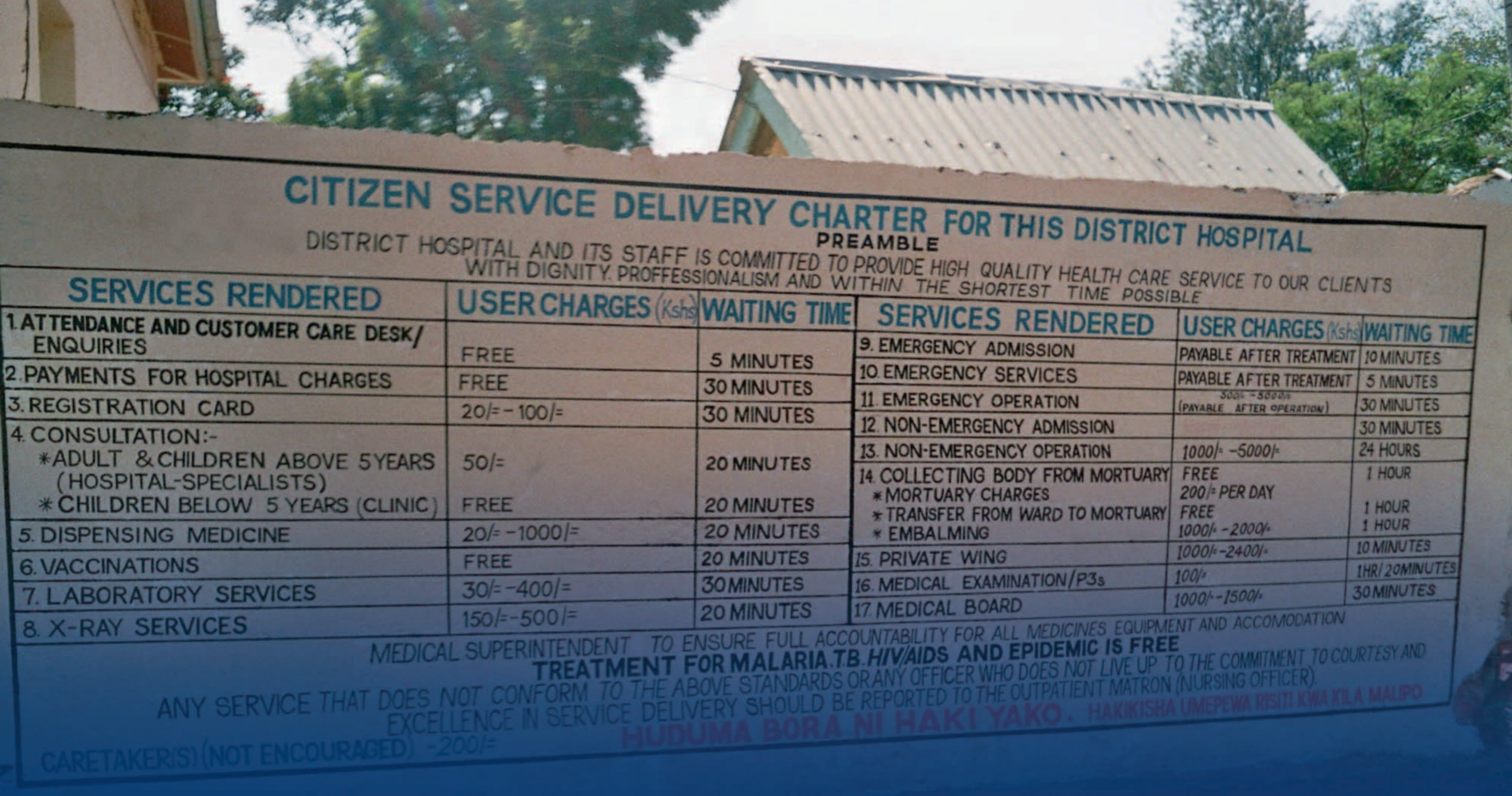

The Tool to Estimate Patients' Costs

Table of contents

TBCTA
The Tuberculosis Coalition for Technical Assistance

USAID
FROM THE AMERICAN PEOPLE

Tool to estimate patients' costs

1 Introduction

2 Literature Review

3 Review SE indicators

4 List of indicators to be measured

5 Questionnaire

6 Guidelines adaptation

7 Guidelines methods sampling interviewer training

8 Guidelines interpretation of results

9 Guidelines interventions

Separated PDF-printfiles of these chapters 1 t/m 9

10 Epi Info data entry template.MDB

Epi Info is needed to open this file.

Epi Info can be downloaded: http://www.cdc.gov/epiinfo/downloads.htm

11 Results summary calculation sheet.xls

12 Presentation of results example.ppt

back to index

Tool to Estimate Patients' Costs

Introduction

Contents

Acknowledegements
Background and Context
The tool and its parts
Types of Costs
Limitations of the tool

The Tool to Estimate Patients' Costs has been developed by KNCV Tuberculosis Foundation, the World Health Organization and the Japan Anti-Tuberculosis Association from 1. October 2007 until 30.September 2008, coordinated by KNCV. Its development was funded by the Tuberculosis Control Assistance Program TB CAP www.tbcta.org. We aim to continuously improve the tool and will be therefore grateful for any suggestion or comment. In this case, please write to Verena Mauch mauchv@kncvtbc.nl.

1. Acknowledgements

We would like to thank and acknowledge the following individuals and organizations for their contributions:

Naomi Woods (Hertie School of Governance Berlin)
Beatrice Kirubi (KEMRI, Kenya)
Eveline Klinkenberg (KNCV Tuberculosis Foundation)
Bertha Nhlema-Simwaka (REACH Trust, Malawi)
Olivia Oxlade (McGill University, Canada)
Gillian Mann (Kadale Consultants, UK)
Andrea Pantoja (WHO Stop TB Geneva)
Delia Boccia (FIND Diagnostics, Geneva)

2. Background and Context

Tuberculosis is a disease that disproportionately affects the poor. TB programs therefore need to ensure that the economically and socially disadvantaged groups do not face barriers that keep them from seeking treatment. In addition, TB programs need to ensure that TB doesn't stand at the beginning of a spiral into (deeper) poverty.

By addressing barriers and reasons for delay to timely diagnosis and treatment by the NTP, costs to TB patients, particularly among the poor, can be effectively reduced. The Poverty Sub-Working Group of the Stop TB Partnership has therefore decided to develop a tool which can assist TB programs to estimate the costs of TB patients before and during diagnosis and during treatment by the NTP.

The aims of the tool are to:

1. To make economic constraints to individuals and households more apparent.
2. To Provide means to assess the impoverishing impact of TB on patients and their families.
3. To establish an evidence-base upon which subsequent interventions can contribute to poverty reduction, increased equity in access to diagnosis and treatment, increased case detection, better treatment adherence

As a first step in developing the Tool, a literature review on studies dealing with patients' costs and methodologies employed was conducted. The objective of the review was to provide a detailed account of research findings at which stage what kinds of costs are incurred. The findings of the review formed the basis and context upon which the tool has been developed. The Tool to Estimate Patients' Costs integrates findings of the review with respect to types of costs, magnitude of costs, specific cost items and indicators to be measured.

3. The Tool and its parts – The tool consists of:

1. **Introduction** (this document)
2. **Detailed literature review on patient cost studies**

The literature review provides a comprehensive overview of past research on patient costs, conceptual frameworks, definitions of different types of costs, approaches to measure the costs of illness, income data and income indicator usage, knowledge gaps and methodological problems. We developed the tool according to findings of the literature review. It is recommended to first read the review in order to understand the tool, and the choice of questions in the questionnaire.

3. **Brief review of socioeconomic indicators**

This short review of was prepared to identify the most important se indicators to ask for in the questionnaire. With the help of these indicators, it will be easier to understand the se background of patients, their vulnerability and their ability to cover the costs they incur due to TB. The review also lists useful literature on se indicators and their usage.

4. **List of indicators to be measured**

This list basically shows what we will know after analysing the results obtained from patient interviews with the questionnaire. It therefore gives you a quick overview what kind of information will be generated through application of the questionnaire.

5. **Generic questionnaire to be adapted to local circumstances**

The questionnaire is the heart of the tool. It is designed to interview patients about their costs due to TB. It has been successfully tested in Kenya in 2008. It is a generic version which needs to be adapted to the respective country and setting. Please refer to the guidelines on adaptation and methods when adapting the questionnaire.

6. Guidelines on adaptation to local circumstances

These guidelines give advice on translating the questionnaire and pretesting it, and it lists and explains all questions that need to be adapted to the local setting.

7. Guidelines on methods, sampling and training of interviewers

These guidelines provide a step by step overview of the methodology that needs to be adhered to in order to produce replicable results. They highlight important points to be considered when the sample, sample size, the target group and in- and exclusion criteria are chosen, data is analyzed and the training of interviewers is planned; in addition, they provide examples of other patient cost survey methods and the coding of questions. It is recommended to refer to a qualitative and quantitative research methods book in addition to these guidelines; respective literature is listed and a quick guide and explanation to the Epi Info data entry template can be found here as well.

8. Guidelines on interpretation of results generated by the questionnaire

These guidelines will help you to interpret your findings - what the results actually mean. The guidelines address each type of costs and related issues such as gender, socioeconomic questions, affordability, productivity etc that were measured by the questionnaire; in each section, the respective indicator and the questions that were used to measure the costs are listed - to guide you through each topic on a step-by-step basis. In addition, the guidelines provide an overview of income indicator usage and income data with links to databases and websites, so that you can compare your income data results with those of other sources.

9. Guidelines on possible interventions

This table will help to think of measures to address the issues found through the analysis of the data generated by the patient interviews. It lists problems and related possible action points and therefore works in the sense of: if you found this, you could think of doing that. The conceivable action points and recommendations are based on the WHO guideline *Addressing Poverty in TB Control.*

10. Epi Info template for data entry

In order to alleviate data entry after patient interviews, we have developed a template which is aligned with the generic questionnaire. The software Epi Info can be downloaded for free on the CDC website. The template needs to be still adapted in line with the adaptations made to the questionnaire according to the local setting.

11. MS Excel template to summarize results

This Excel template lists all types of costs measured by the questionnaire. You can enter results and it will give you a nice overview of all costs incurred and it calculates for you aggregated costs and summaries. This will make it easier for you to report on the costs found by your study. It is recommended to look at this template in the preparation stage of your study to get an idea what the end result should look like.

back to index

4. Types of costs

The three main types of costs are:
1. charges for health services,
2. transport, accommodation and subsistence and
3. lost income, productivity and time.[1]

Individuals suffering from TB are often ill in their most economically-productive age, which poses a significant economic burden on the household. Poor people have longer pathways to care and costs of accessing care are generally higher before than after diagnosis.[2] Relative costs for poor people as a percentage of their income is much higher than for non-poor patients, although aggregate real costs may be smaller.[3] Out-of-pocket costs for public and private healthcare services may stand at the beginning of a spiral into poverty for many families and exacerbate the poverty of the already-poor. This situation has been termed the "the medical poverty trap"[4].

Stratification of patients along several indicators (gender, geography, socioeconomic status) is therefore necessary to obtain an accurate picture of the economic situation facing TB patients.

<u>Direct costs:</u>

- □ Travel, food, accommodation during visits to care givers for seeking help in private and public sector including pharmacies, traditional healers etc. before diagnosis by the program
- □ Expenditures on medicines, special foods, tests before diagnosis by the program
- □ Travel forth and back for tests and receiving test results
- □ Food and “special foods”
- □ Guardian costs
- □ Diagnostic tests (if not provided for free)
- □ Additional informal payments
- □ Charges for drugs
- □ ‘Under the table’ fees
- □ Costs due to hospitalization
- □ Travel, food, accommodation for follow up tests
- □ Travel, food for DOT visits (if applicable)
- □ Travel, food for medicine collection visits (if applicable)
- □ Consultation / user fees (if applicable)
- □ Guardian costs (person accompanying the patient to health center)
- □ Informal payments (if applicable): additional diagnostic tests, drugs
- □ Additional costs due to (parallel) treatment sought by other providers
- □ Additional costs for TB-HIV co-infected patients
- □ health insurance up front payments to be reimbursed later (if applicable)

---
[1] WHO 2005
[2] Nhlema et al 2003, Kamolratanakul 1999, Rajeswari et al 1999
[3] Nhlema et al 2003, Kemp et al 2007
[4] Dahlgren & Whithead 2006

back to index

Indirect costs:

- ☐ Income reduction due to missed work days/hours, lost job, loss of time to seek job, uptake of less paid labor due to illness
- ☐ Reduced household activities (or cost of other household member replacing household work)
- ☐ Missed work for guardian/DOT supporter
- ☐ Decreased productivity
- ☐ Coping costs: use of savings, reduction of food intake, assets are sold, extra job, kids drop out of school to work, debt / loans

5. Limitations of the tool

The tool can only reach those who, in the end, reached a health facility which provides DOTS. It does not reach those who have not sought help or begun treatment or defaulted due to high costs. Unless specifically defaulters are targeted and interviewed, the tool is biased towards those who have somehow been able to afford treatment and all that is related. Depending on the place of the interview, automatically a certain group of patients is excluded. The results of the tool will be heavily dependent on the districts and facilities where patients are interviewed; If the sampling strategy was purposive, the results will not be representative for all TB patients, but only for those considered poor or who live in the chosen districts; this needs to be taken into consideration when interpreting the results and designing interventions based on the results.

"This study/report/audio/visual/other information/media product (specify) is made possible by the generous support of the American people through the United States Agency for International Development (USAID). The contents are the responsibility of TB CAP and do not necessarily reflect the views of USAID or the United States Government."

"The Global Health Bureau, Office of Health, Infectious Disease and Nutrition (HIDN), US Agency for International Development, financially supports this workshop/ document/ training etc. through TB CAP under the terms of Agreement No.GHS-A-00-05-00019-00."

TBCTA The Tuberculosis Coalition for Technical Assistance

USAID FROM THE AMERICAN PEOPLE

back to index

Tool to Estimate Patients' costs

Literature Review / Discussion paper

© KNCV Tuberculosis Foundation / Verena Mauch 2008

Table of Contents:

1. Conceptual Framework, Definitions, Approaches 1
   Conceptual Framework 1
   Definitions 3
   Approaches to measure the cost of illness 4
   Limitations of the review 7
2. Pre-Diagnosis Costs 9
3. Diagnosis / Pre-treatment Costs 11
4. Treatment Costs 13
5. Total Costs 16
6. HIV-Coinfection Costs 18
7. Gender 18
8. Summary of study results 20
9. Methodological issues in designing the tool 23
   Income Indicator usage 24
   Income data 25
   Remaining Questions 25

Annex I Literature List 27

1. Conceptual Framework, Definitions, Approaches

1.1. Conceptual Framework

Tuberculosis is a disease that disproportionately affects the poor. TB programs therefore need to ensure that the economically and socially disadvantaged groups do not face barriers that keep them from seeking treatment. In addition, TB programs need to ensure that TB doesn't stand at the beginning of a spiral into poverty. The question therefore is how a TB program can target the poor and alleviate their financial burden.

The WHO guideline *Addressing Poverty in TB Control* (2005) lists four different kinds of barriers to access care: geographical, social/cultural, health system and economic barriers. The three main types of costs are: 1) charges for health services, 2) transport, accommodation and subsistence and 3) lost income, productivity and time.[1] Individuals suffering from TB are often in their economically most productive age, which poses a significant economic burden on the household. Poor people have longer pathways to care and costs of accessing care are generally higher before than after diagnosis.[2] Relative costs for poor people as a percentage of their income is much higher than for non-poor patients, although aggregate real costs may be smaller.[3] Out-of-pocket costs for public and private health-care services may stand at the beginning of a spiral into poverty for many families and exacerbate the poverty of the already-poor. This situation has been termed the "the medical poverty trap"[4]. Stratification of patients along several indicators (gender, geography, socioeconomic status) is therefore necessary.

By addressing barriers and reasons for delay to timely diagnosis and treatment by the NTP, costs to TB patients, particularly among the poor, can be effectively reduced. The Poverty Sub-Working Group of the Stop TB Partnership has therefore decided to develop a tool which can assist TB programs to estimate the costs of TB patients before and during diagnosis and during treatment by the NTP. The tool to assess patients costs will make economic constraints to individuals and households more apparent. With the help of more adequate information on patient costs, it will be easier to design targeted, alleviating measures.

The tool should

- be a feasible and realistic tool,
- be applicable world-wide
- permit national programs to estimate the costs for TB patients before & during diagnosis and during treatment
- relate to all sectors providing TB care
- consider costs due to HIV-Co-infection

<u>The aims of the tool are:</u>

- To make economic constraints to individuals and households more apparent.

- To provide means to assess the impoverishing impact of TB on patients and their families.

- To establish an evidence-base upon which subsequent interventions can contribute to poverty reduction, increased equity in access to diagnosis and treatment, increased case detection, better treatment adherence

[1] WHO 2005
[2] Nhlema et al 2003, Kamolratanakul 1999, Rajeswari et al 1999
[3] Nhlema et al 2003, Kemp et al 2007
[4] Dahlgren & Whithead 2006

Objective of the literature review

As a first step to develop the tool, a literature review on studies dealing with patients costs and methodologies employed has been conducted. The objective of this review is to provide a detailed account of research findings at which stage what kinds of costs are incurred. The findings of the review will form the basis and context upon which the tool will be developed.

Literature was identified through searches of meta-databases such as PubMed/Medline, EBSCO host, Elsevier, Science Direct and to a large extent through examining bibliographies and references of published material. Publications in English, French and German, with a special focus on publications since 1990 were sought.[5] Inclusion criteria were applied to identify studies that had dealt with low or middle-income countries or with methodologies employed to measure cost of illness (including studies not dealing with TB). Studies exclusively dealing with costs to healthcare providers were excluded. Studies were screened for methods employed, stage of diagnostic/treatment process when costs were assessed and findings related to delays and indirect and direct costs for patients or households. This yielded a total number of 29 studies. In addition, three studies that only deal with patient delays were included for comparison of delay times. The studies cover the following countries:
Africa: Malawi, Zambia, Sierra Leone, Ghana, South Africa, Ethiopia, Tanzania, Gambia, Uganda
Asia: India, Thailand, Myanmar, Bangladesh, Viet Nam, China
Latin America: Haiti, Bolivia
Europe: --

1.2. Definitions

Studies on the cost of illness to patients or households aim to get a comprehensive idea of illness costs incurred by patients. Illness costs are broken down into direct and indirect costs. Direct costs are out-of-pocket costs linked to seeking diagnosis and treatment including medical expenses, fees, transport, accommodation and food expenditures. Indirect (opportunity) costs differ from financial cost as they include the cost of foregone income due to the inability to work because of the illness and loss of time due to visits to health facilities, time spent on the road to and at health facilities, lost productivity and loss of job. Another approach used by the Commission on Macroeconomics and Health (2001) includes the translation of loss of well-being of a patient into economic cost. This can be subdivided into three parts a) the reduction in market income due to the disease, b) the reduction in longevity, c) the reduction in psychological well-being (pain and suffering).
Besides direct and indirect costs, a third category of costs are those incurred through coping strategies (coping costs) of a household to meet daily requirements despite extra expenditures or loss of income. These include the sale of assets, taking up debt, saving on food or other items, taking a child out of school to care for the patient or taking up another job (Russell 2004).

The economic unit is either the individual or the household. Since direct and indirect illness costs fall on the caregiver and the patient, the household is generally the preferred unit of analysis, but

[5] Search keywords included TB + patient cost, household cost, cost diagnosis, spending, treatment cost, affordability, cost, cost-effectiveness,TB-HIV, Coinfection, HIV, DTC, VTC, cost evaluation, expenditure, socioeconomic, care barriers, treatment affordability, financial costs, economic costs, economic burden, economic impact, access to treatment, economic evaluation, methods cost evaluation, healthcare costs.

data is often collected on a per capita level. This review subdivides costs incurred into the stages where they occur:

1) Before Diagnosis
2) During Diagnosis / Pre-Treatment
3) During Treatment

The causal linkages of these factors are depicted by Russell (2004), p.148:

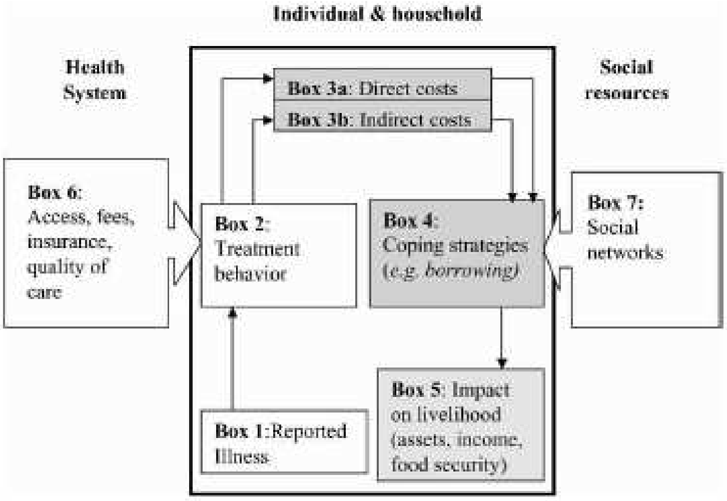

FIGURE 1. Conceptual framework for analyzing the economic burden of illness for households.

At the stage of boxes 1 and 2, decisions are made whether and how treatment is sought as a response to the event of illness. The health system is captured in Box 6. Direct costs capture expenditures related to seeking treatment while indirect costs are loss of labor time for patients and their caregivers. The severity of illness and characteristics of health services affect direct and indirect costs and influence access to and choice of provider. The cost burden and coping strategies of struggling with this burden (mobilizing resources outside the household such as credit – box 7) determine household assets and impoverishing processes, hence the link between illness and poverty.[6]

1.3. Approaches to measure the cost of illness (Malaney 2003)

There are four approaches to measure the cost of illness: The Human Capital Method, the Willingness to Pay model, the Production Function approach and the Friction Cost method. The first two are the classic ones deriving from the 1960s. All of them but the last assess the cost of illness to an individual as well as to society. The following summary will focus specifically on the aspects related to patients cost and neglect some of the aspects mentioned in the literature regarding macroeconomic measurements of the cost to society.

The **Human Capital Method** (HCM) estimates the cost to society of lost future productivity, discounted to the present. The calculations aim at a sum of future earnings of the premature

[6] Russell 2004

dead by looking at life expectancy, labor force participation and average salary data. This is sometimes called the 'top-down-approach'. It includes direct and indirect costs. Indirect costs are productivity losses, measured by estimating income foregone due to morbidity and mortality. The cost of morbidity is the value of lost workdays. Future earnings are discounted to assess the present value of lost income. (One dollar in a year from now is worth less, than a dollar today, taking into account the cost of capital during this year.) Calculations should include lost value of unpaid work in the household, but it is almost never done, due to its difficulty of assessment. The same holds true for the assessment of pain and suffering.

The standard formula for the total costs of illness is:
Cost of illness = private medical costs + non-private medical costs[7] + forgone income + pain and suffering

The HCM has been criticized of inaccuracy when assessing productivity: where productivity is lost, labor substitution by other family or community members happens. Labor then falls disproportionately on women. Second, it does not incorporate forgone household activity and leisure time. In addition, the use of wages as measure of productivity is criticized. Hence, the HCM approach, though used widely, struggles with capturing costs that are not easily measurable in numeric terms.

The **Willingness To Pay Method** (WTP) deduces (by means of household surveys or revealed preferences) the monetary value that a person associates to variations in risk of illness (or death). It is therefore sometimes called the bottom-up approach. It incorporates the cost of pain and suffering, since people are expected to include them when evaluating how much they would pay to reduce their risk of illness or death. Malaney (2003) notes that the cost of an illness on welfare of the household can be determined by the value the household would put on avoiding the disease. This would capture lost productivity, treatment costs, forgone leisure time and pain and suffering.
It has been argued[8] that, in comparison with WTP, the HCM understimates the economic burden of disease on households.

Ability vs. Willingness to Pay
According to Russell (1996), costs of accessing healthcare are affordable, when service utilization is not deterred for financial reasons and opportunity costs don't cause levels of consumption and investment go below minimum needs in the short run. Fabricant et al (1999) considers expenditures as affordable if they have no lasting effects on health, economic or social status on the household. There seems to be consensus though that 3-5% of annual income spent on healthcare expenditures are affordable.[9] Russell (1996) argues that willingness to pay is not equal to ability to pay for the poor, because they might be willing but unable and therefore compensating by sacrificing on nutrition and other important items. Jack (2000) describes the decision of the individual to seek diagnosis to be based on the weighting of the benefit of early detection with the cost. The weighting changes with the severity of symptoms. When the symptoms are bad enough for the expected benefit of diagnosis to outweigh the cost, medical attention will be sought. Reducing the costs of seeking care will help to induce individuals to seek care early. Willingness to pay for treatment is therefore correlated with income and costs of treatment with availability of services.

---

[7] "Non-private medical care costs are public expenditures on both prevention and treatment of the disease" (Malaney 2003, 5)
[8] Malaney 2003
[9] Russell 1996, Russell 2004, Jack 2000, McIntire 2005, Fabricant et al 1999

Once household resources are known, the NTP can determine whether the cost of seeking and obtaining TB care is affordable.

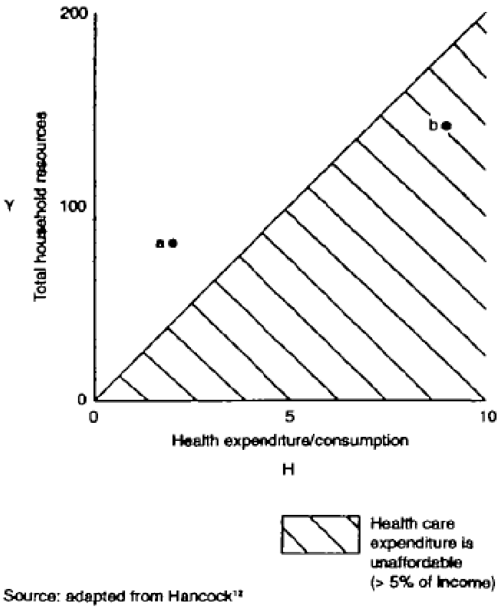

Graph 1 (Russell 1996):

Graph 1 shows total household resources on the Y-axis and health expenditures on the x-axis. If total costs of TB care are too high (in the shaded area), they are no longer affordable.

The Theory behind Willingness to Pay

Graph 2 shows the budget constraint, t1, reavealing the combination of goods given the patient's income and prices of goods, t1 is the patient's ability to pay (ATP). Its function is:
– (price of healthcare / price of food). M1 is the indifference curve that describes a person's preferences (willingness to pay WTP). At Point A, the person is healthy and needs little healthcare, at point B, the person is sick. With a decrease of a patient's income due to inability to work, the budget constraint shifts left, t2. Given the new budget constraint t2, the patient cannot obtain his/her desired level of consumption (point B). The intersections of the y and x axis with the budget constraint are calculated as income/price of food and income/price of healthcare.

The difference D between the ability to pay (t1) and the willingness to pay (m2), is the cost of TB on the welfare of the household, including pain and suffering, so to say the true cost of TB. If the household's income decreases (t2), the difference D becomes even greater. M2 is the maximum the patient is willing to pay given his resources; t1 is the maximum the he/she is able to pay.

At the intersection of t1 with the y-axis, the patient spends 50 on food and 0 on healthcare. At the intersection with the x-axis, he spends 50 on healthcare and 0 on food. This scheme can be applied to any point in the graph. Hence, the ability to pay are all possibilities on t1, for example:

A: 20 H and 30 F = 50
B: 35 H and 15 F = 50
C: 25 H and 25 F = 50

Whereas the willingness to pay is along m2, for example E (35 H and 35 F = 70). The difference between ability and willingness to pay D is 70-50 = 20. The true cost of TB including pain and suffering is therefore 70. The method to ask for the willingness to pay is most likely the only method to capture costs of TB on household welfare and to show the difference between actual and desired consumption due to TB.

Graph 2:

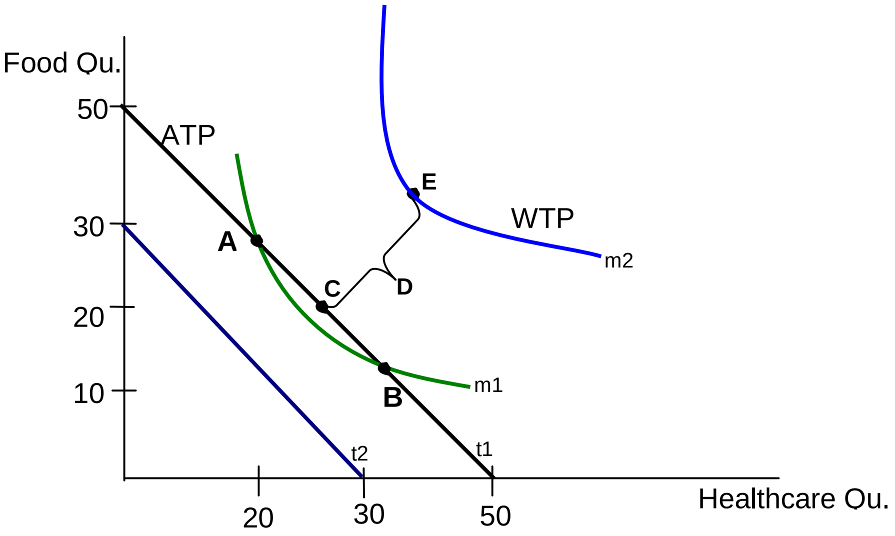

This means in practice, if the NTP knows the budget of a (poor) household, the costs of treatment and the costs for food (or other items), it can see whether the costs of TB care are affordable.[10]

The **Production Function Approach** takes into account decreased productivity of chronically ill patients and decreased productivity of workers not being able to recover fully before taking up work again. The method estimates a production function of an output unit (crop land, factory, household) and by using regression analysis to evaluate the loss of output due to the illness. This model has not been used much because of the difficulty to specify and data problems. It does not capture direct costs of a disease and is therefore not of much use to estimate patients' costs.

The **Friction Cost Method** assesses indirect costs by determining the time span organizations need to restore the initial production level after production was lost due to disease (Drummond 1997, Koopmanschap et al 1995). This period differs according to level and education of the worker, location and industry. The friction cost method takes the viewpoint of the firm and of society and is therefore not useful to assess costs on patient level.

[10] The intersections of the y and x axis with the budget constraint are calculated as income/price of food and income/price of healthcare.

Summary of approaches:

Approaches to measure cost of illness	Approach	Strength	Limitation
Human Capital Method	estimates the cost to society due to morbidity and mortality of lost future productivity, discounted to the present. Cost of morbidity is the value of lost workdays.	Easy to use, since data on forgone income can be easily collected. Most widely used compared to other methods.	Doesn't capture labor substitution by family members, forgone household activities and leisure time. Use of wages as measure of productivity criticized for inaccuracy. Underestimates burden of disease on household.
Willingness to Pay	deduces the monetary value that a person associates to variations in risk of illness (or death). How much would you pay to prevent illness?	Incorporates burden to household of treatment costs, loss of productivity, cost of pain and suffering and value of forgone leisure	subject to personal interpretations of question; social desirablity bias in answering. Willingness to pay ≠ Ability to pay
Production Function	estimates a production function of an output unit and evaluates loss of output due to prevalence of illness.	Captures effect of illness on productivity also when ill people return to work before being completely cured.	difficulty to specify and to collect meaningful data. Doesn't capture direct costs of a disease.
Friction Cost Method	assesses indirect costs by determining time span organizations need to restore initial production level after production was lost due to disease.	Captures indirect costs of prevalent disease to society.	takes the viewpoint of the firm; not useful to assess costs on patient level.

Summed up, the only two methods which are applicable to measuring costs from the individual perspective, relatively easy to use and which provide meaningful data, are the Human Capital Method and the Willingness to Pay model. The limitations of these two models will need to be addressed when designing the tool.

1.4. Limitations of the review

- *HIV Coinfection*

The literature available on additional costs because of HIV coinfections is very limited. Literature on the cost of HIV/AIDS to patients during their lifetime is available, but the nature of the disease (lifelong) makes it difficult to associate these costs with costs incurred by TB patients.

- *Paediatric, unemployed and elderly TB patients and household work*

A limited number of studies (Beyers 1994, Geetharamani 2001) focus on children and economic value of housework. Most studies just capture salaries which excludes unpaid work in the household and the unemployed who lose time to seek new employment. Additional costs to a household due to elderly patients living in the same household are only captured through guardian costs of travel, accommodation and food.

- *Similar tools to improve to service delivery*

There is a multitude of studies on targeting the poor, developing measures to estimate cost burdens and socioeconomic measures, measuring access to healthcare and developing proxies for assessing income. However, the author of this review has not found any study which has reflected on the practicability, design, and impact on service delivery of such a tool for an NTP or

other programs. This does not mean that such studies do not exist. Further research is needed here.

- *Comparative value*

Costs associated with seeking treatment, receiving diagnosis and the treatment itself can be divided into three phases: Costs incurred prior to diagnosis, costs incurred during diagnosis (prior to treatment) and costs incurred during treatment. It is difficult to compare study results, because of different methodological approaches and study designs. The same holds true for the distinction between the three periods in which costs are incurred. Some studies include diagnostic costs when calculating treatment costs, whereas others include diagnostic costs when assessing the pre-diagnostic burden. Therefore, studies discussing more than one period will be mentioned in both periods.

Other difficulties to compare studies include:

- **different usage of currencies**. Most studies converted results into US$. Results of three studies[11] that reported in local currencies (Thai Baht, Indian Rupees) were converted by the author of this review into US$ to allow comparison (exchange rate as reported in study, alternatively year of study). However, Dollar amounts can only give a very rough idea of costs, because of different inflation levels in each country and the value change of the US$ relative to other currencies over time, different purchasing power parities and different price levels of services.

- **different definitions and measures of direct costs** (including transport or only medical expenses). Some studies distinguished between direct expenditures and medical costs on drugs and laboratory tests. In these cases, medical expenditures were included into direct costs.

- **different definitions and measures of indirect costs** (months affected by illness or actual days off work, integration of non-remunerated work). Some studies measured indirect costs as self-reported forgone salaries, some as self-reported forgone income, some estimated forgone income on the basis of hours worked per day or per month; some used the average wage rate, some used GDP or GNI per capita, some used income levels estimated by household surveys. Some included caretaker indirect costs, though most didn't. Data is presented as percentage of monthly or annual household or per capita income. Few calculated lost productivity into forgone income. It is impossible to standardize all of these results. Hence, all numbers declared as indirect costs in these studies are compared as such. Most studies assessed indirect costs according to self-reported data collected through surveys or interviews. Coping costs are not included in indirect cost measurements, but are mentioned here separately.

- **different units of analysis** (household or per capita).

[11] Rajeswari 1999, Muniyandi 2005, Kamolratanakul 1999

back to index

2. Costs Incurred Before Diagnosis

Delays

Many studies have documented delays from the onset of symptoms to diagnosis[12]. These delays do not only increase the infectivity of a patient and lead to more serious illness[13] by the time the patient presents him/herself, but also represent a time span in which additional costs are incurred. The delay can be subdivided into the period from onset of symptoms until a patient presents him/herself at a health facility (patient delay), the period from presentation to diagnosis (diagnostic delay), the period between first visit to a health facility and diagnosis (doctor delay), the period between diagnosis and beginning of treatment (treatment delay) and the time span between first visit and start of treatment (health system delay).[14]

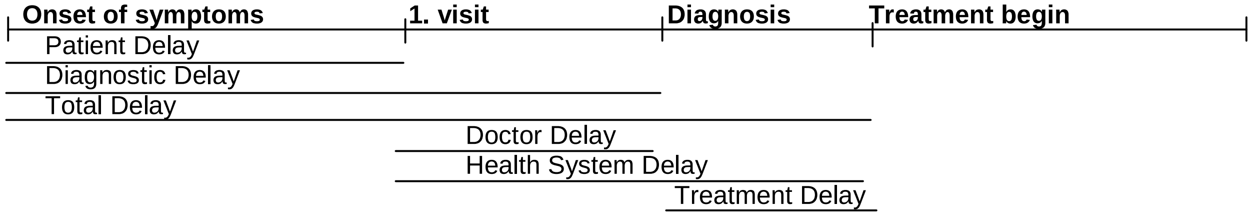

Studies suggest however, that the delay caused by the health system is longer than the patient caused delay[15], in Ghana especially for rural dwellers.[16] Demissie et al's (2002) study found a much shorter system delay than patient delay, but it recognizes its findings to be low compared to findings of other studies. Lonnroth et al (1999) found patient and provider delay to be more pronounced in urban areas because of more options, weak referral and coordination mechanisms.

The times of delay from onset of symptoms to diagnosis vary from study to study to a great extent, ranging from 8 weeks[17] to 19 months.[18] The majority reports time spans between 2-4 months for adults[19] and 1 month for children[20] with the number of health encounters during this time ranging between 2.7 and 6.7[21].

Direct costs

Patients repeatedly cited lack of money in general and transportation costs in particular as reasons for delay.[22] In Needham's study (2004) in Zambia, transportation costs amounted to 16% of mean monthly income. The amount of transportation varies with urban or rural location of the patient. Patients in Zambia living outside Lusaka spent twice as much on transport than those living in the proximity of or in Lusaka.[23] In his study of 687 patients in Thailand, Kamolratanakul (1999) determined the direct average cost to households between $55-225. This

[12] Kemp et al 2007, Needham et al 2001, 2004, 1998, Lawn et al 1998, Beyers et al 1994 to name just a few
[13] Gibson et al 1998, Lawn et al 1997, 1998
[14] Lawn et al 1998, Karim 2007
[15] Beyers et al 1994, Lawn et al 1998, Needham et al 2004, Squire 2005, Lonnroth et al 2001, Equi TB 2005, Lonnroth et al 2007
[16] Lawn et al 1998
[17] Needham 1998, 2004, Demissie 2002
[18] Gibson et al 1998
[19] Lawn 1998, Needham 1998, 2001, 2004, Demissie 2002
[20] Beyers 1994
[21] Equi TB 2005, Gibson et al 1998, Kemp et al 2007, Needham et al 1998, 2004
[22] Squire et al 2005, Needham et al 2004, 1998, Gibson et al 1998, Croft 1998, Muniyandi 2005
[23] Needham et al 1998

is in line with findings by Jacquet al (2006) in Haiti. Russell (2004) determines direct costs to amount to 5-21% of annual household income.

Several studies have reported pre-diagnostic costs incurred through visits to private providers, pharmacies and traditional healers.[24] Needham (2004) notes that TB infected persons in his study in Malawi paid 10% of their monthly income to traditional healers for consultation. These visits were associated with longer delays between 15 and 41 days. Lonnroth et al (2001) found that 65% of the study population in Vietnam had been treated with TB drugs by more than one provider, while 50% of patients opted for private care. The public program was perceived to be more time consuming with repeated visits for diagnostics and long-waiting times.

Indirect Costs
Most of the studies dealing with prediagnostic costs focussed on lost income, days of work lost, decreased earning ability, change in work and costs associated with coping strategies.[25] Indirect cost estimates range from $16[26] (Malawi, Bangladesh, India, Zambia) to $68[27] (Malawi, Zambia). In these studies, workdays lost range from 18[28] to 48[29] (both Zambia) for patients and 9 to 13[30] for guardians. Muniyandi (2005, India) reports 71% of patients borrowing money to cope with costs. Croft (1998, Bangladesh) reports similar findings with half of her study population coping by selling land and livestock or taking out a loan.

Total Costs
Total costs (direct and indirect) for patients prior to diagnosis, measured as % of mean monthly income, varies between 127% (Needham et al 1998) and 135% (Kemp et al 2007). In Dollar terms, this amounts to 59 and 29 US$ respectively. Lonnroth et al (2001) found total costs to lie between 15 and 77 US$. Needham (1998) found caregiver costs to amount to 31% of mean monthly income. Striking is the difference between costs expressed in mean monthly income between the poor and non-poor in Malawi.[31] Whereas the poor have associated costs amounting to 244% of their monthly income on accessing diagnosis, the non-poor's burden amounts to 129%. Needham (1998) reports economic loss to be especially grave for self-employed persons.

Studies consulted on pre-diagnostic costs:

Beyers et al (1994). Delay in the diagnosis, notification and initiation of treatment and compliance in children with tuberculosis. Tuberc Lung Dis 75, 260-265.

Boillot & Gibson (1995). The formal and informal costs of tuberculosis in Sierra Leone, TuberLungDis 76, supplement 2, 114.

Croft & Croft (1998). Expenditure and loss of income incurred by tuberculosis patients before reaching effective treatment in Bangladesh. Int J Tuberc Lung Dis 2, 252-254.

Demissie et al (2002). Patient and health service delay in the diagnosis of pulmonary tuberculosis in Ethiopia. BMC Public Health 2(23).

[24] Kemp et al 2007, Muniyandi 2005, Lonnroth et al 2001, Needham et al 2004
[25] Kemp et al 2007, Muniyandi 2005, Croft 1998, Needham 1998, 2004
[26] Kemp et al 2007, Needham 1998
[27] Jacquet et al 2006
[28] Needham et al 1998
[29] Needham et al 2004
[30] Kemp et al 2007
[31] Kemp et al 2007

EQUI-TB Knowledge Programme (2005). Barriers to accessing TB care: how can people overcome them? Liverpool School of Tropical Medicine.

Floyd et al (2006). Cost and cost-effectiveness of PPM-DOTS for tuberculosis control: evidence from India. Bull World Health Organ 84(6), 437-45.

Gibson et al (1998). The cost of tuberculosis to patients in Sierra Leone's war zone. Int J Tuberc Lung Dis 2(9), 926, 731.

Jacquet et al (2006). Impact of Dots Expansion on tuberculosis related outcomes and costs in Haiti. BMC Public Health 6, 209.

Kemp et al (2007). Can Malawi's poor afford free tuberculosis services? Patient and household costs associated with a tuberculosis diagnosis in Lilongwe. Bulletin of the WHO 85, 580-585.

Kamolratanakul et al. (1999). Economic impact of tuberculosis at the household level. Int J Tuberc Lung Dis 3,596-602.

Karim et al (2007). Gender differences in delays in diagnosis and treatment of tuberculosis. Health Policy & Planning 22, 329-334.

Lambert (2005). Delays to treatment and out-of-pocket medical expenditure for tuberculosis patients, in an urban area of South America. Ann Trop Med Parasitol.99(8), 781-7.

Lawn et al (1997). Pulmonary tuberculosis: diagnostic delay in Ghanaian adults. Int Jour Tub & Lung Dis 2, 635-640.

Lonnroth al (2001). Can I afford free treatment? Perceived consequences of health care provider choices among people with tuberculosis in Ho Chi Minh City, Vietnam. Soc Sci Med 52, 935-948.

Lonnroth et al (2007) Social franchising of TB care through private GPs in Myanmar: an assessment of treatment results, access, equity and financial protection. Health Policy and Planning 22, 156-166.

Muniyandi (2005). Costs to patients with tuberculosis treated under DOTS programme. Indian J of Tub 52, 188-196.

Needham et al (2001). Socio-economic, gender, and health services factors affecting diagnostic delay for tuberculosis patients in urban Zambia. Trop Med Int Health 6, 256-259.

Needham (1998). Barriers to tuberculosis control in urban Zambia: the economic impact and burden on patients prior to diagnosis. Int J Tuberc Lung Dis 2, 811-7.

Pantoja et al (forthcoming). Free TB treatment at a high cost: economic burden faced by TB patients in a public-private mix initiative in Bangalore, India.

Squire et al. (2005) Lost smear positive pulmonary tuberculosis cases; where are they and why did we lose them? Int J Tub and Lung Dis 9(1), 25-31.

Zhang et al (2007). Persistent problems of access to appropriate, affordable TB services in rural China: experiences of different socio-economic groups. BMC Public Health 7, 19.

back to index

3. Costs During Diagnosis / Pre-Treatment

Costs specifically measured for diagnosis are difficult to discern and rarely addressed by themselves. Most studies combine the assessment of diagnostic costs with costs occurring before diagnosis or during treatment. Netherless, if costs occurring at the diagnostic stage were reported separately in studies, they are reported here as well.

Delays
Two studies in Malawi and Bolivia[32] looked specifically at pretreatment delays, which includes the delay before diagnosis. Lambert et al (2005) found pretreatment delays to be mostly due to the provider (14 weeks) and less due to the patient (9 weeks), with an average total of 12.6 weeks. Lonnroth et al (2007) reports 15 days delay from first contact with the health system to treatment due to the provider and a total of 26 days (3.7 weeks) from onset of symptoms to treatment start. Kemp et al (2007) in Malawi found that 4.5-6 visits to health centers were necessary before treatment was started.

Direct costs
Direct costs incurred during diagnosis range widely, per household between $2 in Tanzania[33] and $57 in Thailand[34], and per patient between $6 in India[35] and $130 in Bangladesh[36]. The majority lies between $10-50. Russell (2004) found pre-+post-diagnosis direct costs to amount to 8-13% of annual household income.

Cases of overprescriptions, charges for drugs (India) and informal payments occur (China, Sierra Leone and Tajikistan)[37], though this seems to depend strongly on the setting. Kemp et al (2007) found informal payments to be rare in Malawi. In China, often-times patients are charged for additional, unnecessary drugs and diagnostic tests leading to a substantial increase of the costs to patients (personal communication).

Indirect costs
Indirect costs similarly have a wide-spread range between $16 in Malawi[38] and $115 in Bangladesh[39], with the majority lying between $10-30, however only three studies specify indirect costs incurred exclusively at the diagnostic stage. Workdays lost up to diagnosis lie between 20[40] and 48 days[41].

Total Costs
A better picture emerges when looking at total costs incurred during diagnosis. Here, the majority lies between $10 and 30, but reports are going all the way up to $245 in Bangladesh.[42] Total costs as a percentage of income are 135% of mean monthly household income in Malawi[43] and 31% of annual income per capita in Bangladesh[44], 58% for the poor in Myanmar[45].

[32] Kemp et al 2007, Lambert et al 2005
[33] Wyss et al 2001
[34] Kamolratanakul 1999
[35] Rajeswari 1999
[36] Croft & Croft 1998
[37] Gibson et al 1998, Equi-TB 2005, Muniyandi 2005, Boillot & Gibson 1995, Falkingham 2003
[38] Kemp et al 2007
[39] Croft & Croft 1998
[40] Kemp et al 2007
[41] Rajeswari 1999
[42] Ibid
[43] Kemp et al 2007
[44] Croft & Croft 1998

Interestingly, Kemp et al (2007) found that the poor spent 244% of monthly income on diagnosis which is 110% more than the average. This emphasizes the fact, that averages do not adequately represent the economic burden of the poor.

Studies consulted on diagnostic / pre-treatment costs:

Boillot & Gibson (1995). The formal and informal costs of tuberculosis in Sierra Leone, TuberLungDis 76(supplement 2), 114.

Croft & Croft (1998). Expenditure and loss of income incurred by tuberculosis patients before reaching effective treatment in Bangladesh. Int J Tuberc Lung Dis 2, 252-254.

EQUI-TB Knowledge Programme (2005). Barriers to accessing TB care: how can people overcome them? Liverpool School of Tropical Medicine.

Falkingham (2003). Poverty, out-of-pocket payments and access to health care: evidence from Tajikistan. Social Science & Medicine 58, 247-258.

Gibson et al (1998). The cost of tuberculosis to patients in Sierra Leone's war zone. Int J Tuberc Lung Dis 1998, 2(9), 926,731.

Kamolratanakul et al. (1999). Economic impact of tuberculosis at the household level. Int J Tuberc Lung Dis 3,596-602.

Kemp et al (2007). Can Malawi's poor afford free tuberculosis services? Patient and household costs associated with a tuberculosis diagnosis in Lilongwe. Bulletin of the WHO 85, 580-585.

Lambert et al.(2005). Delays to treatment and out-of-pocket medical expenditure for tuberculosis patients, in an urban area of South America. Ann Trop Med Parasitol.99(8), 781-7.

Lonnroth et al (2007). Social franchising of TB care through private GPs in Myanmar: an assessment of treatment results, access, equity and financial protection. Health Policy and Planning 22, 156-166.

Lonnroth et al (1999). Delay and discontinuity - a survey of TB patients' search of a diagnosis in a diversified health care system. Int J Tuberc Lung Dis 3(11), 992-1000.

Muniyandi (2005). Costs to patients with tuberculosis treated under DOTS programme. Indian J of Tub 52, 188-196.

Rajeswari et al (1999). Socio-economic impact of tuberculosis on patients and family in India. Int J Tuberc Lung Dis 3, 869-77.

Uplekar (1996). Tuberculosis patients and practitioers in private clinics. Bombay: the foundation for research in community health.

Wyss et al (2001). Cost of tuberculosis for households and health care providers in Dar es Salaam, Tanzania. Trop Med Int Health 6, 60-8.

[45] Lonnroth et al 2007

4. Costs During Treatment

Delays

A multitude of studies deals with treatment delays (capturing patient and system delay) defined as the time elapsed from onset of symptoms until the beginning of treatment. Results from studies that looked at patient costs found treatment delays (capturing herein also prediagnostic and diagnostic delay) to lie between 6 and 16 weeks.[46] Taking into account however that prediagnostic delays alone were reported to last already between 5 and 17 weeks, treatment delays should theoretically exceed prediagnostic delays, assuming that time elapses between diagnosis and start of treatment. On the other hand, these numbers provide us at least with a time frame of 5-17 weeks in which we can assume that the patient incurrs costs due to forgone income because of his/her inability to work and time lost during his/her efforts of seeking treatment.

Direct costs

Direct costs vary widely across studies which depends of course on local prices for food, transport etc. Starting from $5 in Tanzania[47] up to $150 in Haiti[48], with the majority between $20 and 50. Items requiring most of the expenditures are travel and food and for drugs if they are not provided for free.[49] In India[50], expenditures on health visits, travels and drugs were found to be higher among urbanites than among patients living in rural areas. In South Africa, Sinanovic (2003) identified DOT visits to be the item accumulating most of the costs. She further found that workplace supervision was much less costly ($11) than clinic supervision ($111). In India[51], direct costs were found to be higher for women than for men.

In Thailand, out-of-pocket direct expenditures of the very poor for diagnosis and treatment amounted to 15% of their annual per capita income,[52] in Haiti, they were 49%.[53]

Medical expenses amounted to 40% of annual income of Chinese households, for low-income households, they were equivalent to 112% of annual income[54]. Russell (2004) determined direct post-diagnosis costs to amount to 18.4% of annual household income. Moalosi (2003) investigated in Botswana direct costs for care-givers and found that home-based care cost 23% less for care givers than hospitalization.

Hospitalization:

Floyd et al (1997) found that the average length of hospital stay for patients in South Africa was 17.5 days for those patients entering community DOT after discharge and two months for conventional hospitalization during the intensive phase. Admission to hospital constituted 76% of patient cost, with a day in hospital costing the patient $4. DOT at hospital was more expensive than DOT at health clinic or community level. A hospital visit cost the patient 5 hours. Okello et al (2003) had similar results in Uganda: one day hospital cost $1.30 for the patient, overall, hospital based care was more expensive than community based care ($252 vs $206). Moalosi et al (2003) found home-based care in Botswana to be 42% cheaper for patients than hospital-based care; while the average hospital stay with home-based care was 21 days, it was 93 days with hospital-based care. Needham (1998) found caregiver costs to be greater for in-patients than for

---

[46] Lambert et al 2005, Lonnroth et al 1999, Lienhardt et al 2001, Rajeswari 2002, Lawn et al 1998
[47] Wandwalo 2005
[48] Jacquet et al 2006
[49] Kamolratanakul 1999, Wyss et al 2001, Sinanovic 2003
[50] Rajeswari et al1999
[51] Rajeswari et al 1999
[52] Kamolratanakul 1999
[53] Jacquet el al 2006
[54] Zhang et al 2007

out-patients, in his 2004 study however, he attributes less delays for patients seeking hospitalization.

Indirect costs
Indirect costs in Dollar terms amount to $7[55] - $50[56], with a tendency towards $20[57]. Productivity in household or occupation drops by ca 30%. $150-200 or 15%-20% of annual household income is lost; patients cannot work for ca 2-4 months and 20-75% of patients incur some form of debt.

<u>Summary:</u>

Direct Cost as % of income: Household: 18 -112%
Per capita: 15% - 49%

Indirect Costs:

Productivity

- Household activities and childcare falls by 30%-40%
- 74% loss of working capacity

Income

- loss of 2-45% of annual household income (majority ca. 15%)
- 9-112% of annual per capita income (majority 10-30%)
- 15-89% of GDP/capita (majority ca 15%)

Work time lost:

- 2 -14 months (majority 2-4 months)
- One person per household cannot follow an occupation during period of illness

Coping costs:

- 11% of children discontinued school, 8% took up employment (India, Geetharamani 2001)
- 55 – 75% of patients or households borrow money or incur other forms of debt

Total Costs
Total costs (direct and indirect) of TB treatment to patients are reported to be between $9.5[58] and $202[59], with the majority being below $100[60]. Total cost of TB treatment is found to be between 20 and 30% of annual household income.[61] Sinanovic (2003) found community based care more affordable than clinic based care (due to DOT visits). Wandwalo's (2005) cost-effectiveness study in Tanzania supports this finding. On average, ss+ patients had to make 58 visits to a health facility for DOT, a ss- had a total of 24 visits, compared to a patient under community DOT with a total of 10 visits.

Studies consulted on treatment costs:

Ahlburg (2000). The economic impact of TB: ministerial conference Amsterdam, WHO.

[55] Wandwalo 2005
[56] Jacquet et al 2006
[57] Wandwalo 2005, Muniyandi et al 2005
[58] Gibson et al 1998
[59] Jacquet et al 2006
[60] Muniyandi et al 2005, Uplekar 1996, Wandwalo et al 2005, Rajeswari et al 1999
[61] Ramachandran et al 1997, Croft & Croft 1998

Croft & Croft (1998). Expenditure and loss of income incurred by tuberculosis patients before reaching effective treatment in Bangladesh. Int J Tuberc Lung Dis 2, 252-254.

Fryatt (1997). Review of published cost-effectiveness studies on tuberculosis treatment programmes. Int J Tuberc Lung Dis 1(2),101-109.

Floyd et al (1997). Comparison of cost-effectiveness of directly observed treatment and conventionally delivered treatment for tuberculosis: experiences from rural South Africa. BMJ 315(7120), 1395-6.

Geetharamani et al (2001). Socio-economic impact of parental tuberculosis on children. Ind J Tub 48, 91-94.

Gibson et al (1998). The cost of tuberculosis to patients in Sierra Leone's war zone. Int J Tuberc Lung Dis 1998, 2(9), 926, 731.

Jacquet et al (2006). Impact of Dots Expansion on tuberculosis related outcomes and costs in Haiti. BMC Public Health 6, 209.

Kamolratanakul et al (1999). Economic impact of tuberculosis at the household level. Int J Tuberc Lung Dis 3,596-602.

Lambert et al (2005). Delays to treatment and out-of-pocket medical expenditure for tuberculosis patients, in an urban area of South America. Ann Trop Med Parasitol.99(8), 781-7.

Lonnroth et al (2007). Social franchising of TB care through private GPs in Myanmar: an assessment of treatment results, access, equity and financial protection. Health Policy and Planning 22, 156-166.

Lonnroth et al (1999). Delay and discontinuity - a survey of TB patients' search of a diagnosis in a diversified health care system. Int J Tuberc Lung Dis 3(11), 992-1000.

Lienhardt et al (2001). Factors affecting time delay to treatment in a tuberculosis control porgramme in a sub-saharan african country: the experience of the Gambia. Int.Jour of Tub and Lung Dis 5, 233-239.

Lawn et al (1997). Pulmonary tuberculosis: diagnostic delay in Ghanaian adults. Int Jour of Tub and Lung Dis 2, 635-640.

Moalosi et al (2003). Cost-effectiveness of home-based care versus hospital care for chronically ill tuberculosis patients, Francistown, Botswana. Int J Tuberc lung Dis 7, 80-5.

Muniyandi (2005). Costs to patients with tuberculosis treated under DOTS programme. Indian J of Tub 52, 188-196.

Okello et al (2003). Cost and cost-effectiveness of community-based care for tuberculosis patients in rural Uganda. Int J Tuberc Lung Dis 7(9), 72-79.

Rajeswari al (1999). Socio-economic impact of tuberculosis on patients and family in India. Int J Tuberc Lung Dis 3, 869-77.

Ramachandran et al (1997). Economic impacts of tuberculosis on patients and family. Tuberculosis Research Centre, Indian Council of Medical Research, Chennai, South India.

Russell (2004). The economic burden of illness for households in developing countries: a review of studies focusing on Malaria, Tuberculosis and Human Immunodeficiency Virus/Acquired Immunodeficiency syndrome. Am J Trop Med Hyg 71 (Suppl2), 147-155.

Saunderson (1995). An economic evaluation of alternative program designs for tuberculosis control in rurual Uganda. Social Science and Medicine 40, 1203-1212.

Sinanovic et al (2003). Cost and cost-effectiveness of community-based care for tuberculosis in Cape Town, South Africa. Int J Tuberc Lung Dis 7(9), 56-62.

Uplekar (1996). Tuberculosis patients and practitioers in private clinics. Bombay: the foundation for research in community health.

Wandwalo et al (2005). Cost and cost-effectiveness of community based and health facility based directly observed treatment of tuberculosis in Dar es Salaam, Tanzania. Cost Eff Resour Alloc. 14, 3-6.

Wyss et al (2001). Cost of tuberculosis for households and health care providers in Dar es Salaam, Tanzania. Trop Med Int Health 6, 60-8.

Zhang et al (2007). Persistent problems of access to appropriate, affordable TB services in rural China: experiences of different socio-economic groups. BMC Public Health 8(7), 19.

5. **Total Costs** (Pre-Diagnosis, Pre-Treatment, Treatment)

In order to assess the costs of a complete treated TB episode relative to its parts, it is useful to review studies that have investigated the total costs of TB treatment while including costs incurred at each level. Two reviews by Ahlburg (2000) and Russell (2004) are particularly useful.

Direct costs

Direct costs vary again strongly by country, starting with $24 in Zambia[62] up to $346 in China[63]. It can be observed however that the most frequent dollar range is between $60 and 130[64]. Direct costs of TB amount to 3.7 – 15% of annual income (highest for the poor).[65] In comparison, Jackson (2006) found that direct costs to be equivalent to 55% of annual household income in China. Direct cost burdens are exacerbated by widespread use of private providers, particularly in urban settings.[66] In addition, direct costs are unevenly distributed across households, minorities bearing high costs compared to the majority of the population.[67] Russell (2004) attributes more meaning to median figures than to mean figures, however, mean figures are mostly presented in the literature. Costs vary strongly and the mean is therefore determined by outliers.

Indirect costs

Indirect costs vary between $28[68] and $1384[69] with the majority lying in the range of $100-500[70]. Russell found TB indirect costs to amount to 5-8% of annual household income, Rajeswari (1999) to 26%. In terms of workdays lost, Needham (1996) reports 2 weeks in Zambia whereas others

[62] Needham et al 1996
[63] Jackson et al 2006
[64] Russell 2004, Rajeswari 1999, Kamolratanakul 1999, Ahlburg 2000
[65] Kamolratanakul 1999, Rajeswari 1999, Russell 2004
[66] Russell 2004
[67] Russell 2004
[68] Russell 2004
[69] Wyss 2001
[70] Russell 2004, Rajeswari 1999, Kamolratanakul 1999, Wyss et al 2001, Jackson et al 2006, Jacquet et al 2006, Ahlburg 2000

report an average loss of 8 -12 weeks[71]. Interestingly, according to Ahlburg (2000), treated patients lose 2 months of work compared to untreated ones losing 12 months. In respect to coping costs, Jackson (2006) reports 66% of patients borrowing money from relatives or friends, 45% sold assets and 8% borrowed money from banks. Rajeswari (1999) reports 14% of annual household income forgone for debt redemption.

Total Costs

The economic burden of TB can be well-understood with the help of % of income. The poor spend a far greater proportion on meeting basic needs (food etc) whereas the non-poor have more disposable income. The burden of each $ spent is significantly higher for the poor. Russell (2004) deems a cost burden of more than 10% of annual household income to be already catastrophic for a household's financial situation. Taking this into account, study results point to the enormous burden of households and individuals of 20-30% of monthly income[72] and 10-90% of annual household income[73] (highest for the very poor), the majority being approximately between 10% and 40%[74]. Ahlburg (2000) determined the cost of morbidity of treated TB to be 15% of GDP per capita.

Studies consulted on Total TB costs for patients:

Ahlburg (2000). The economic impact of TB: ministerial conference Amsterdam, WHO.

Jacquet et al (2006). Impact of Dots Expansion on tuberculosis related outcomes and costs in Haiti. BMC Public Health 6, 209.

Jackson et al (2006).Poverty and the economic effects of TB in rural China. Int J Tuberc Lung Dis.10(10), 1104-10.

Kamolratanakul et al. (1999). Economic impact of tuberculosis at the household level. Int J Tuberc Lung Dis 3, 596-602.

Muniyandi (2005). Costs to patients with tuberculosis treated under DOTS programme. Indian J of Tub 52, 188-196.

Needham (1996). Economic barriers for TB patients in Zambia. The Lancet 348(9020), 134-5.

Rajeswari et al (1999). Socio-economic impact of tuberculosis on patients and family in India. Int J TUberc Lung Dis 3, 869-77.

Russell (2004). The economic burden of illness for households in developing countries: a review of studies focusing on Malaria, Tuberculosis and Human Immunodeficiency Virus/Acquired Immunodeficiency syndrome. Am J Trop Med Hyg 71 (Suppl2),147-155.

Wyss et al (2001). Cost of tuberculosis for households and health care providers in Dar es Salaam, Tanzania. Trop Med Int Health 6, 60-8.

[71] Rajeswari 1999, Kamolratanakul 1999, Ahlburg 2000
[72] Needham et al 1996, Muniyandi et al 2005
[73] Russell 2004, Rajeswari 1999, Jacquet et al 2006, Ahlburg 2000
[74] Russell et al 2004, Kamolratanakul 1999, Ahlburg 2000, Rajeswari 1999

6. TB/HIV Coinfection costs

There are plenty of studies on mortality cost of HIV deaths to society, but there is hardly any literature on costs for TB patients that are coinfected with HIV. Jacquet et al (2006) bases the time range in which there is a loss of productivity on the number of years anticipated to survive after development of active TB and number of years survived with HIV infection before developing active TB, with a total average survival of 9.8 years. In his review study, Beck et al (2001) reports a community loss of 0.4 potential years of life lost per person in India; In Uganda, incremental lost income per person with Aids death was $12.256 in 1992. With the lack of studies on this topic, the incremental costs of an HIV coinfection are difficult to determine. In terms of indirect costs, greater mortality, lower productivity, long-term reduced ability to earn and prolonged morbidity of TB-HIV coinfected persons are definite, especially if the patient presents him/herself late.[75] Annex I specifies extra cost items due to an HIV infection.

7. Gender

A number of studies emphasize higher costs for women than for men. Women take longer to seek care (patient delay) [76] due to stigma and social exclusion, heavier workloads, prioritization of other family members over own well-being, lack of independence, inaccessibility to financial resources and powerlessness in decision-making[77]; they experience longer provider, diagnostic and treatment delays[78]; they are engaged in more activities that need to be replaced in the household, while girls replace these activities more than boys[79]. In addition, women have higher direct costs than men, because they often need somebody to accompany them[80], they are less mobile and have less financial resources[81] and women experience greater loss of income probably because of more lost work days[82].

Studies consulted on TB-HIV Coinfection costs and Gender

Currie et al (2005). Cost, affordability and cost-effectiveness of strategies to control tuberculosis in countries with high HIV prevalence. BMC Public Health 5, 130.

Beck et al (2001). The cost of HIV treatment and care. A global review. Pharmacoeconomics 19(1), 13-39.

Jacquet et al (2006). Impact of Dots Expansion on tuberculosis related outcomes and costs in Haiti. BMC Public Health 6, 209.

Lawn et al (1997). Pulmonary tuberculosis: diagnostic delay in Ghanaian adults. Int Jour Tuber & Lung Dis 2, 635-640.

Needham et al (2001). Socio-economic, gender, and health services factors affecting diagnostic delay for tuberculosis patients in urban Zambia. Trop Med Int Health 6, 256-259.

[75] Lawn et al 1997
[76] Equi-TB 2005, Needham 2001, Karim et al 2007
[77] Lawn et al 1998, Needham 2001, Karim et al 2007
[78] Needham et al 2001, Karim et al 2007
[79] Kemp et al 2007
[80] Muniyandi 2005
[81] Needham 2001
[82] Needham 1998

Karim et al (2007). Gender differences in delays in diagnosis and treatment of tuberculosis. Health Policy & Planning 22, 329-334.

8. Summary of Study Results

Pre-Diagnostic costs:

Delay:	2-4 months, 3-7 health encounters
Direct costs:	$55-225, 5-21% of annual household income
Indirect costs:	$16-68, 18-48 days lost
Total:	127 per capita -135% household mean monthly income

Types of costs (direct):

- Travel, food, accommodation during visits to care givers for seeking help in private and public sector including pharmacies, traditional healers etc.
- Expenditures on medicines, special foods, tests

Indirect:

- Income reduction due to missed work days/hours, lost job, loss of time to seek job, uptake of less paid labor due to illness
- reduced household activities (or cost of other household member replacing household work)
- missed work for caretaker
- decreased productivity
- coping costs: use of savings, reduction of food intake, assets are sold, extra job, kids drop out of school to work, debt / loans

Diagnosis/Pre-treatment costs:

Delay:	3 months (including pre-diagnosis), 5 health encounters
Direct costs:	$10-50, 8-13% of annual household income
Indirect costs:	$10-30, 20-48 days lost
Total:	ca 135% mean montly household income, ca 31% annual income per capita

Types of costs (direct):

- travel forth and back for tests and receiving test results
- accommodation
- food and “special foods”
- guardian costs
- diagnostic tests (if not provided for free)
- additional informal payments
- charges for drugs
- user fees
- ‘under the table’ fees

Indirect:

- Income reduction due to missed work days/hours, lost job, loss of time to seek job, uptake of less paid labor due to illness
- reduced household activities (or cost of other household member replacing household work)
- missed work for caretaker
- decreased productivity
- coping costs: use of savings, reduction of food intake, assets are sold, extra job, kids drop out of school to work, debt / loans

Treatment costs:

Delay: 1 ½ - 4 months (incl pre-diagnostic and diagnostic delays), total of 5-17 weeks
Direct costs: $20-50, 15-49% of annual per capita income, 40-112% of annual household income
Indirect costs: ca $20, 2-4 months of lost work, 15-20% annual household income, 20-75% of patients incur debt, productivity loss of 30%
Hospitalization: 17 – 21 days home-based care; 60-93 days hospital care, $1.3 – 4 per day
Total: below $100, 20-30% of annual household income

Types of costs (direct):

- Costs due to hospitalization
- Travel, food, accommodation for follow up tests
- Travel, food for DOT visits (if applicable)
- Travel, food for medicine collection visits (if applicable)
- Consultation / user fees (if applicable)
- Guardian costs
- Informal payments (if applicable): additional diagnostic tests, drugs
- Additional costs due to parallel treatment sought by other providers
- Additional costs for TB-HIV coinfected patients: travel and food to ARV clinic, screening intake, test result, medicine collection
- health insurance up front payments to be reimbursed later (if applicable)

Indirect:

- Income reduction due to missed work days/hours, lost job, loss of time to seek job, uptake of less paid labor due to illness
- reduced household activities (or cost of other household member replacing household work)
- missed work for caretaker
- decreased productivity
- coping costs: use of savings, reduction of food intake, assets are sold, extra job, kids drop out of school to work, debt / loans

Total Costs TB episode:

Direct costs: $60-130, 4-15% of annual per capita income
Indirect costs: $100-500, 5-16% of annual household income, 2-3 months lost work, 70% borrow
Total: 20-30% of monthly income (household and per capita), 10-90% of annual household income, 15% of GDP/capita

It can be clearly seen that costs (indirect and direct) incurred at the prediagnostic stage are higher than during the following stages. Delays in the prediagnostic stage are most costly for the patient and society, for the patient is still infectious and his/her health and productivity are deteriorating. Direct costs frequently pass the 10% of household income and Indirect costs often exceed direct costs. This has been shown by an influential review study on household costs due to illnesses.[83] For direct costs, the **most expensive items are travel (especially DOT visits to health facilities), food and private sector charges**. **In total, TB patients lose 2-4 months of income because of the inability to work, mostly during the treatment phase.**

Coping Costs
In regard to coping costs, two studies[84] have summarized the order of coping strategies used by patients and neatly complement the findings presented in this review. They both found that the

[83] McIntire 2006
[84] Sauerborn 1996, McIntire 2006

household first reallocates tasks. Second, savings (if any) are used; third, consumption is reduced (mostly food); fourth, assets are sold (land, livestock); fifth, loans are taken up and last, income is diversified (additional job); the community is asked for help as a last resort. This means on the one hand, that the household would be a more suitable unit of analysis and on the other, that debt (and interest payments) is not inherently the logical consequence of cost constraints. The sale of assets however reduces future income and therefore TB can stand in the beginning of a spiral into deeper poverty.

Summed up, costs to patients depend on:

- The nature, frequency and duration of the illness
- The healthcare seeking behaviour of affected individuals
- The type of treatment (community vs health facility DOT)
- The direct and indirect costs of diagnosis and treatment
- Responses and mobilization of resources
- Resources available to the household or patient

9. Methodological Issues In Designing The Tool

9.1. Income Indicator Usage

In order to estimate the impact costs have on a patient, we first need to know the amount that a patient can afford to spend on TB. That is, we need to be able to judge what % of the patient's income is associated with costs of TB. There are two ways to approach this: either to ask patients with the means of surveys and interviews about their income or consumption expenditures or to use standardized measures of income, such as average wage rates, GNI per capita, or income levels. These standardized measures are usually obtained through household surveys or data supplied by UNDP, the World Bank[85], UNICEF[86], DHS[87] or WHO[88]. However, these databases do not provide recent income data on all countries.

For the purpose of developing a tool for NTP managers to estimate patient costs, both approaches face difficulties. The bottom-up approach requires substantial financial and human resources to conduct representative surveys. During the past years, researchers have become more and more hesitant to use self-reported income data and found asset based assessments households surveys more useful and representative.[89] The top-down approach is more practical, but average wage rates and GNI/capita don't provide the NTP with information specifically about the most vulnerable parts of the population, that this tool aims to target; they only represent averages and therefore underestimate the poor's burden[90]. A good and often used alternative is

---

[85] Gwatkin et al 2007: Socio-economic differences in health, nutrition and population. World Bank.http://www1.worldbank.org/prem/poverty/health/ World Development Report 2006: Selected development indicators http://www-wds.worldbank.org/external/default/WDSContentServer/IW3P/IB/2005/09/20/000112742_20050920110826/additional/841401968_2005082630000823.pdf

[86] UNICEF Multiple Indicator Cluster Surveys: http://www.childinfo.org/MICS2/natlMICSrepz/MICSnatrep.htm

[87] Demographic and Health Survey DHS: http://www.measuredhs.com/countries/start.cfm

[88] WHO/World Health Surveys: www.who.int/healthinfo/survey/en/index.html

[89] Verbal communication with researchers from McGill and Liverpool School of Tropical Medicine

[90] Russell 1996

back to index

recent data on household incomes obtained through country-level household surveys. Not every country has conducted such surveys, for they are expensive.[91]
Researchers have struggled with these problems and found different solutions. Filmer (2001) determined household assets (in India) to be closely enough related to consumption expenditures to serve as a proxy for the latter. Hence, surveys not on income, but on assets or consumption may serve the same purpose. Zhang et al (2007) used the indicator 'annual household medical expenditures during the last 12 months' as a proxy for estimating the costs for diagnosis and treatment. Fabricant et al (1999) used housing type, food expenditure and self-estimates as proxies for income levels in Sierra Leone and found that a one-day agricultural wage correlates with the average price of an out-patient visit in some countries and therefore serves as an indicator for affordable treatment.

Another difficult issue, and therefore often-times left out, is the method to estimate loss of income for individuals active in the household, but not in regular employment or waged activities. Recalling what was said on coping strategies, it is known, that in the short-run, activities are reallocated within the household.[92] In the long-run, however, they will need to be replaced. Drummond (1997) recommends either using the average wage, the cost of replacing the role, or the opportunity cost of production the individual could have contributed to, if he/she was employed. These measures however run the risk of overestimation.

Summed up, what needed is:
- Household or per capita income data (for the poorest quintile and average)
- cost as % of hh & per capita income (derived by household data and cost data)
- willingness to pay / affordability of services

9.2. Income data

Since we are trying to assess the impact of costs on the lowest income quintile of the population, the question remains which income measure to use. There is a whole body of literature on measuring poverty which addresses the same question (from a different angle). There are three basic approaches:

1) Real measures:
    a. **National household budget surveys** – dependent on availability from national statistics office. Whether any surveys have been conducted recently can be seen by searching the International Household Survey Network (IHSN) database[93].
    b. **UNDP Human Development reports** [94] (detailed reports on national situations, distinguishing between urban and rural and by districts, giving information on real per capita expenditure in local currency, adjusted to Purchasing Power Parity).
    c. For Africa, the **Africa Development Indicators** 2006[95] provide recent detailed data.
    d. World Bank **Povcalnet data** by country on average monthly income, headcount of population living in poverty, Gini index[96] Compare this data with GNI/capita and poverty line of 1$ a day.

[91] It is argued that household surveys don't include the poorest of the poor, because many households in urban slums are not interviewed, and where it is considered to be risky or difficult to identify household entities (UN Research Institute for Social Development 2007)
[92] Drummond 1997
[93] http://www.surveynetwork.org/home/?lvl1=activities&lvl2=catalog&lvl3=surveys
[94] http://hdr.undp.org/en/
[95] Household surveys p103ff http://siteresources.worldbank.org/INTSTATINAFR/Resources/ADI_2006_text.pdf

e. **Living Standards Measurement Studies**[97] by the World Bank provide datasets of household surveys for many countries and guidelines for interpreting this data.
f. **Gross national income per capita** for each country by World Bank[98]. If Gini coefficient (from Povcalnet) is low, GNI/capita can be used, don't use it with a high Gini. If % of population living below poverty line is small, GNI/capita can be used, otherwise don't use it.
g. **Gross domestic product per capita** for each country by UN Statistics Division on social indicators[99]
h. **ILO reports** on wages of unskilled/agricultural labor[100] per country

2) Absolute estimates:
   a. Absolute Poverty line: World Bank measures of absolute poverty: **1$ a day** (31 $ per month) at purchasing power parity. This can be compared to GNI/capita and mean monthly income on Povcalnet. If they are similar, GNI/capita can be used. If they are very different, don't use GNI/capita.
   b. **Basket of goods** (minimum necessities): food vs. non-food items – dependent on availability from national statistics office or also in Human Development Reports

3) Relative estimates:
   a. Relative Poverty lines: These are usually set at **50-70% of median household income**[101]. GNI could be used as baseline as well. If Gini coefficient is low, this measure can be meaningful, not so with a high Gini.[102]

With all of these measures, <u>the most recent and meaningful data</u> should be taken;

<u>Prioritization:</u>
1) Recent (5 years or less old) **national household surveys** specifying income data according to geographical location or income quintiles of the population
2) For Africa: the **Africa Development Indicators** 2006, for the rest of the world **Human Development report** data
3) If none of the above are recent or available, compare GNI/capita, GDP/capita with World Bank poverty line and relative poverty line (60% of median or average household income), taking into account % of population living below poverty line and Gini coefficient. Make meaningful choice which one to use.
4) If available, take unskilled or agricultural wage from ILO database per country.

Example: Rwanda:

1) IHSN search yields no result.
2) Search on National institute of Statistics Rwanda website yields no result.
3) Search in Africa Development Indicators 2006 yields no result (country not listed)
4) Search on Human Development Report website yields following result: National Report Rwanda 2007

---

[96] http://iresearch.worldbank.org/PovcalNet/jsp/index.jsp
[97] http://www.worldbank.org/LSMS/
[98] http://web.worldbank.org/WBSITE/EXTERNAL/DATASTATISTICS/0,,contentMDK:20535285~menuPK:1390200~pagePK:64133150~piPK:64133175~theSitePK:239419,00.html
[99] http://unstats.un.org/unsd/demographic/products/socind/inc-eco.htm
[100] http://laborsta.ilo.org/
[101] Combat Poverty Agency 2006, OECD
[102] Cut off points for high and low Ginis could be (arbitrarily taken) at 20. Low Gini <20; high Gini >20

- P. 15ff: Reaching the poor, p.19: average income in bottome quintile in 2006: Rwf18,900 /year
- P. 20: average income of a poor person has remained virtually unchanged since 2001 at Rwf150 per day against Rwf146 per day in 2001.

9.3. Remaining questions

1. The number of trips to health facilities varies considerably with the availability of DOT services. Community DOT hardly requires travel and food expenditures, whereas health facility DOT does. DOT three times weekly requires less trips than daily intake. The number of trips will also vary depending on length and nature of pre-treatment delays, the practiced procedure, opening hours of diagnostic and treatment facilities and the distance from facility to home of the patient. Delay times are periods in which the patient's productivity is already reduced and indirect costs are incurred. It is difficult to generalize the amount of reduction in productivity across all patients. Another question is whether coping costs can or should be included. It would be easy to calculate additional costs due to debt and interest payments, but it is much more difficult to estimate income loss due to sale of assets or children dropping out of school.

2. We have to assume that not all patients will be able to resume their occupation after the end of treatment and not all patients will complete their treatment. Some will have lost their job, some will have defaulted, some will not be cured. Especially HIV infected TB patients are affected by higher morbidity, less productivity and are therefore subject to continuing indirect costs.

3. None of the studies consulted accounted for a learning curve within a family or community. That is, once a family or community member has undertaken the odyssey from healer to private practitioner to public health facility and has learned about the disease and its symptoms, opening hours of facilities, costs, DOT and – most importantly – cure as treatment result, he/she will share this knowledge with his/her family and community and will be of assistance should another family or community member show TB symptoms. The direct and indirect costs for the second and following patients should therefore be lower than to the first patient.

4. Most tools which were identified during the literature review and which strive for similar aims were survey/questionnaire/interview-based. None of the tools employed (also outside the TB domain) aim to estimate costs without running surveys or operational research projects. This tool should be flexible enough to deliver meaningful data with small sample sizes, not to require too much time to complete and to be adjustable to the national or local context.

5. What about those who don't come at all because of the economic burden of seeking treatment? How could they be reached?

Annex I: List of Reviewed Literature

Akobundu et al (2006). Cost-of-Illness Studies. A review of current methods. *Pharmacoeconomics 24*(9), 869-890.

Ahlburg (2000). *The economic impact of TB*. Ministerial Conference Amsterdam, WHO 2000.

Asch et al (1998). Why do symptomatic patients delay obtaining care for tuberculosis. *Am J Respir Crit Care Med 157*(4), 1244-8.

Beyers et al (1994). Delay in the diagnosis, notification and initiation of treatment and compliance in children with tuberculosis. *Tuberc Lung Dis 75*, 260-265.

Beck et al (2001). The cost of HIV treatment and care. A global review. *Pharmacoeconomics 19*(1), 13-39.

Berman et al (1994). The household production of health: integrating social science perspectives on micro-level health determinants. *Soc Sci Med 38*, 205-215.

Boillot & Gibson (1995).The formal and informal costs of tuberculosis in Sierra Leone. *Int J Tuber Lung Dis 76*, supplement 2, 114.

Creese & Parker (1994). *Cost analysis in primary healthcare. A training manual for program managers*. WHO.

Croft & Croft (1998). Expenditure and loss of income incurred by tuberculosis patients before reaching effective treatment in Bangladesh*. Int J Tuberc Lung Dis 2*, 252-254.

Currie et al. (2005). Cost, affordability and cost-effectiveness of strategies to control tuberculosis in countries with high HIV prevalence*. BMC Public Health 12*(5),130.

Dahlgren & Whitehead (2006). Concepts and principles for tackling social inequities in health. WHO EURO. http://www.euro.who.int/document/e89383.pdf

Demissie et al (2002). Patient and health service delay in the diagnosis of pulmonary tuberculosis in Ethiopia. *BMC Public Health 2*(23).

Drummond et al (1997). *Methods for the economic evaluation of health care programmes*. New York: Oxford UP

De Maio (2000). Income inequality measures. *J Epidemiol Community Health 61*, 849-852.

EQUI-TB Knowledge Programme (2005*). Barriers to accessing TB care: how can people overcome them?* Liverpool School of Tropical Medicine. http://www.healthlink.org.uk/PDFs/tb_barriers.pdf

Guigemde et al (1997). A precise method for estimating the economic costs of Malaria: application of the method in a rural area in Burkina Faso. *Trop Med Int Health 2*, 646-653.

Floyd et al (2006). Cost and cost-effectiveness of PPM-DOTS for tuberculosis control: evidence from India. *Bull World Health Organ 84*(6), 437-45.

Floyd (2003). Costs and effectiveness-the impact of economic studies on TB control. *Int J Tuberc Lung Dis 83*(1-3):187-200.

Floyd et al (1997). Comparison of cost-effectiveness of directly observed treatment and conventionally delivered treatment for tuberculosis: experiences from rural South Africa. *BMJ 315*(7120), 1395-6.

Fabricant et al (1999). Why the poor pay more: household curative expenditures in rural Sierra Leone. *Int J Health Plann Manag 14*, 179-199.

Farmer (1997). Social science and the new Tuberculosis. *Soc Sci & Med 44*(3), 347-358.

Falkingham (2003). Poverty, out-of-pocket payments and access to health care: evidence from Tajikistan. *Social Science & Medicine 58*, 247-258.

Fryatt (1997). Review of published cost-effectiveness studies on tuberculosis treatment programmes. *Int J Tuberc Lung Dis 1*(2), 101-109.

Filmer (2001). Estimating wealth effects without expenditure data – or tears. *Demography 38*, 115-32.

Gallup & Sachs (2000). *The economic burden of Malaria*. Cambridge, MA:Center for Int development, Harvard University CID working Paper No 52.

Gibson et al (1998). The cost of tuberculosis to patients in Sierra Leone's war zone. *Int J Tuberc Lung Dis 2(*9), 731.

Gwatkin & Guillot (2000). *The burden of disease among the global poor*. Human Development Network, The World Bank, Washington DC.

Gwatkin et al (2007). *Socio-economic differences in health, nutrition and population*. World Bank. www.worldbank.org/povertyandhealth

Gwatkin et al. (2005). *Reaching the poor with health, nutrition and population services. What works, what doesn't and why.* World Bank.

Geetharamani et al. (2001). Socio-economic impact of parental tuberculosis on children. *Ind J Tub 48*, 91-94.

Gupta et al (2001). Public health. Responding to market failures in tuberculosis control. *Science 293*(5532):1049-51.

Guiguemde et al (1997). A precise method for estimating the economic costs of Malaria: application of the method in a rural area in Burkina Faso. *Trop Med Int Health 2*, 646-653.

Hanson (2002). *TB, poverty and inequity, a review of the literature* (written for WHO, unpublished)

Hanson et al (2006). *Tuberculosis in the poverty alleviation agenda*. In: Raviglione M (ed.). TB: a comprehensive international approach. New York: Informa Healthcare.

Jack (2001). The public economics of TB control. *Health Policy 57*, 79-96.

Jackson et al (2006). Poverty and the economic effects of TB in rural China*. Int J Tuberc Lung Dis. 10*(10), 1104-10.

Jacquet et al (2006). Impact of Dots Expansion on tuberculosis related outcomes and costs in Haiti. *BMC Public Health 6,* 209.

Kamolratanakul et al (1999). Economic impact of tuberculosis at the household level. *Int J Tuberc Lung Dis 3*, 596-602.

Kamolratanakul et al (2002). Cost analysis of different types of tuberculosis patient at tuberculosis centers in Thailand*. Southeast Asian journal of tropical medicine and public health 33*, 321–30.

Karim et al (2007). Gender differences in delays in diagnosis and treatment of tuberculosis. Health Policy & Planning 22, 329-334.

Kemp et al. (1996). Is TB diagnosis a barrier to care? *R Soc Trop Med Hyg 90*, 472.

Kemp et al (2007). Can Malawi's poor afford free tuberculosis services? Patient and household costs associated with a tuberculosis diagnosis in Lilongwe. *Bulletin of the WHO 85*, 580-585.

Kemp, Boxshall, Nhlema et al (2001). Application of a geographical information system to assess the realtionship between socioeconomic status and access for TB care in urban Lilongwe*. Int J Tuberc Lung Dis 5*, 167

Koopmanschap et al (1995). The friction cost method for measuring indirect cost of disease. *Journal of Health Economics 14*, 171-189.

Lambert et al (2005). Delays to treatment and out-of-pocket medical expenditure for tuberculosis patients, in an urban area of South America. *Ann Trop Med Parasitol. 99*(8), 781-7.

Luhanga et al (2001). Gender differences in access to treatment and caring for TB patients within households. *Int J Tuberc Lung Dis 5*, 167.

Lonnroth et al (2001). Can I afford free treatment? Perceived consequences of health care provider choices among people with tuberculosis in Ho Chi Minh City, Vietnam. *Soc Sci Med 52*, 935-948.

Lonnroth et al (2007) Social franchising of TB care through private GPs in Myanmar: an assessment of treatment results, access, equity and financial protection. *Health Policy and Planning 22*, 156-166.

Lonnroth et al (1999). Delay and discontinuity - a survey of TB patients' search of a diagnosis in a diversified health care system. *Int J Tuberc Lung Dis 3*(11), 992-1000.

Lienhardt et al (2001). Factors affecting time delay to treatment in a tuberculosis control porgramme in a sub-saharan african country: the experience of the Gambia. *Int. Jour of Tub and Lung Dis 5*, 233-239.

Lawn et al (1997). Pulmonary tuberculosis: diagnostic delay in Ghanaian adults. *Int Jour of Tub and Lung Dis 2*, 635-640.

Lawn et al (1997). Delays in the diagnosis of tuberculosis: a great new cost*. Journal of Tuberc Lung Dis 1*, 485-486.

Malaney (2003). *Micro-economic approaches to evaluating the burden of Malaria*. Cambridge, MA: Center for int. development, Harvard University, CID working paper no.99.

Maskus (2003). Ensuring access to essential medicines: some economic considerations. *Spec Law Dig Health Care Law 291*, 9-25.

Mark et al (1990). Productivity, health and inequality in the intrahousehold distribution of food in low-income countries. *The American economic review 80* (5), 1139-1156.

McIntire (2006). What are the economic consequences for households of illness and of paying for healthcare in low- and middle-income country contexts? *Social science and medicine 62*, 858-65.

Muniyandi et al (2006). Estimating provider cost for treating patients with tuberculosis under revised national tuberculosis control programme. *Indian Journal of Tub 53*, 12-17.

Muniyandi et al (2005). Costs to patients with tuberculosis treated under DOTS programme. *Indian J of Tub 52*, 188-196.

Muniyandi et al (2006). Socio-economic dimensions of tuberculosis control: review of studies over two decades from Tuberculosis Research Center. *J Commun Dis.38*(3), 204-15.

Mishra et al (2005). Socio-economic status and adherence to tuberculosis treatment: a case-control study in a district of Nepal. *Int J Tuberc lung Dis 9*(10), 1134-1139.

Moalosi et al (2003). Cost-effectiveness of home-based care versus hospital care for chronically ill tuberculosis patients, Francistown, Botswana. *Int J Tuberc lung Dis 7*, 80-5.

Murray (1990). Tuberculosis in developing countries: burden, intervention and cost. *Bull Int Union Against Tuberculosis 65*(1), 2-20.

Nair et al (1997). Tuberculosis in Bombay: new insights from poor patients. *Health policy Plann 12*, 77-85.

Nhlema et al (2007). Developing a socio-economic measure to monitor access to tuberculosis services in urban Lilongwe, Malawi. *Int J tuberc Lung Dis 11*(1), 65-71.

Nhlema et al (2002). *Systematic Analysis of TB and poverty*. Technical Report, Geneva: Stop TB Partnership, WHO.

Needham et al (2003). Patient seeking care barriers and tuberculosis programme reform: a qualitative study. *Health Policy 67*, 1-15.

Needham et al (1998). Barriers to tuberculosis control in urban Zambia: the economic impact and burden on patients prior to diagnosis. *Int J Tuberc Lung Dis 2,* 811-7.

Needham et al (2001). Socio-economic, gender, and health services factors affecting diagnostic delay for tuberculosis patients in urban Zambia. *Trop Med Int Health 6*, 256-259.

Needham (1996). Economic barriers for TB patients in Zambia. *The Lancet 348* (9020), 134-5.

Okello et al (2003). Cost and cost-effectiveness of community based care for tuberculosis patients in rural Uganda. *Int J Tuberc Lung Dis 7*, 72-79.

Pantoja et al (forthcoming). Free TB treatment at a high cost: economic burden faced by TB patients in a public-private mix initiative in Bangalore, India.

Peabody et al (2005). *The burden of disease, economic costs and clinical consequences of tuberculosis in the Philippines*. Oxford UP and London School of Hygiene and Tropical Medicine.

Rivera & Currais (1999). Income variation and health expenditure. Evidence from OECD countries. *Review of Development Economics 3*(3), 258-267.

Rajbhandary (2004). Costs of patients hospitalized for multidrug-resistant tuberculosis. *International Journal of Tuberculosis and Lung Disease 8* (8), 1012-1016,

Rajeswari et al (1999). Socio-economic impact of tuberculosis on patients and family in India. *Int J Tuberc Lung Dis 3,* 869-77.

Rajeswari et al (2002). Factors associated with patient and health system delays in the diagnosis of tuberculosis in South India. *Int Jour of Tub and Lung Dis 6*, 789-795.

Russell (1996). Ability to pay for health care: concepts and evidence. *Health policy Plann 11*, 219-237.

Russell (2004). The economic burden of illness for households in developing countries: a review of studies focusing on Malaria, Tuberculosis and Human Immunodeficiency Virus/Acquired Immunodeficiency syndrome. *Am J Trop Med Hyg 71* (Suppl2), 147-155.

Saunderson (1995). An economic evaluation of alternative program designs for tuberculosis control in rurual Uganda. *Social Science and Medicine 40*, 1203-1212.

Ramachandran et al (1997). *Economic impacts of tuberculosis on patients and family*. Tuberculosis Research Centre, Indian Council of Medical Research, Chennai, South India.

Sauerborn et al (1996). Household strategies to cope with the economic costs of illness. *Soc Sci Med 43*, 291-301.

Sen, Basu (1972). Economics of Health-The cost of Tuberculosis. *Indian Journal of Tub 19* (4), 144-158.

Sawert (1996). *Health economics*. WHO.

Sawert (2000). *Economic considerations for TB control*. In: Reichman: Tuberculosis, a comprehensive international approach, 799-816.

Singh et al (2002). TB control, poverty, and vulnerability in Delhi, India. *Tropical Medicine and International Health 7*(8), 693-700.

Sinanovic et al (2003). Cost and cost-effectiveness of community-based care for tuberculosis in Cape Town, South Africa. *Int J Tuberc Lung Dis 7*(9), 56-S62

Spence et al (1993). Tuberculosis and Poverty. *BMJ 307*, 759-761.

Squire et al (2005). Lost smear positive pulmonary tuberculosis cases; where are they and why did we lose them? *Int J Tub and Lung Dis 9*(1), 25-31.

Uplekar (1996). *Tuberculosis patients and practitioners in private clinics*. Bombay: the foundation for research in community health.

UN Research Insitute for Social Development UNRISD (2007). *Conference News. Equitable Access to Healthcare and Infectious Disease Control.* Report of an International Symposium 13-15 february 2006, Rio de Janeiro, Brazil.
http://www.unrisd.org/80256B3C005BCCF9/(search)/85B229CD6C973919C125736F002862D8?Opendocument&highlight=2,travassos&fromsearch=yes&query=travassos

Wandwalo et al (2005). Cost and cost-effectiveness of community based and health facility based directly observed treatment of tuberculosis in Dar es Salaam, Tanzania. *Cost Eff Resour Alloc.14*, 3-6.

Wagstaff (1991). On the measurement of inequalities in health. *Social science and medicine 33*, 545-57.

Whitehead (1992). The concepts and principles of equity and health*. Int Jour of health services 22*, 429-45.

Weis et al (1999). Treatment costs of directly observed therapy and traditional therapy for mycobacterium tuberculosis : a comparative analysis *Int J Tb & Lung Dis 3,* 976-984.

WHO (1996). *Cost analysis and cost containment in Tuberculosis control program*. WHO/TFHE/96.1 (Editor Sawert), WHO.

WHO (2005*). Addressing Poverty in TB Control*.
http://whqlibdoc.who.int/hq/2005/WHO_HTM_TB_2005.352.pdf

WHO WPRO (2004). *Reaching the Poor. Challenges for TB Programs in the Western Pacific Region*.

WHO (2004). *Compendium of indicators for monitoring and evaluating national tuberculosis programs*. WHO/HTM/TB/2004.344

World Bank (2007). *Economic Benefit of Tuberculosis Control*. Policy Research Working Paper 4295. Washington DC.

World Bank (2007). *World Development Indicators*. http://www-wds.worldbank.org/external/default/WDSContentServer/IW3P/IB/2005/09/20/000112742_20050920110826/additional/841401968_2005082630000823.pdf

World Bank (2006). *Africa Development Indicators 2006.* http://siteresources.worldbank.org/INTSTATINAFR/Resources/ADI_2006_text.pdf

World Bank (1993). *World Development Report, investing in health.* http://www.dcp2.org/file/62/World%20Development%20Report%201993.pdf

Wurtz (1999). The cost of TB. *Int J Tuberc Lung Dis 3*(5), 382-7.

Wyss et al (2001). Cost of tuberculosis for households and health care providers in Dar es Salaam, Tanzania. *Trop Med Int Health 6*, 60-8.

Zhang et al (2007). Persistent problems of access to appropriate, affordable TB services in rural China: experiences of different socio-economic groups. *BMC Public Health 8*(7), 19.

back to index

Tool to Estimate Patients' Costs

Overview of Socioeconomic Indicators to Measure Living Conditions & Impoverishment

Morris et al (2000) give a very good account on strengths and weaknesses of two approaches to measure wealth and income (which are theoretically different concepts with different trajectories to influence health and informed by different aspects of policymaking).

1) The asset-based approach as a proxy for **wealth**
    - Lists household assets (context-specific items) to elicit a weighted score which can be used to identify the poor from the non-poor.
    - Most countries have an LSMS (Living Standards Measurement Survey by the World Bank)[1], adjusted to local circumstances. Questionnaires can be downloaded from the web to identify a suitable list of assets.
    - Does not take into account the most valuable household items due to difficulty of measurement. This includes land, house, livestock, financial capital and human resources.

2) Total household expenditure as a proxy for **income**
    - Total household expenditure is an accepted alternative to household income in developing country settings.
    - Consumption/expenditure data provides a better proxy for socioeconomic status in low-resource settings than reported income (empirically proven) as it takes items into account which affect consumption but which would not appear if information on income was sought.
    - Where the majority of people are living from daily labor and agricultural work, consumption is easier to measure than income.
    - Includes a short list of key expenditures items.
    - Key items can be taken from LSMS surveys (see above) or analysis of items most highly correlated with total income.
    - Recall bias affecting reported income also affects reported expenditures.

Galobardes et al (2006) provide a good overview for widely used socioeconomic indicators, including their interpretation, meanings, values, strengths, weaknesses and measurements. For our purposes, their account of income and occupation is useful:

Income:
- Individual or household.
- If household: information on family size is needed for a weighted and comparable result.
- It can provide useful information about the poverty level when compared to the national poverty line.
- Disposable income is most useful, though difficult to discern from gross income.
- Income is age-specific. Retired and young people have lower incomes and income tends to grow over time.

[1] http://www.worldbank.org/LSMS/guide/select.html

Occupation:

- Gives information about the social status of an individual and related income.
- Provides information about working conditions and hazards.
- Information on occupation is available in many routine data collections
- Not useful with unpaid, household, informal and illegal work; the unemployed, retired and students.

Not all poverty is alike. There are different kinds of poverty stratification, for example migrants, single mothers, refugees, unemployed persons, slum dwellers, and rural farmers. They all have different socioeconomic characteristics. There are however a set of indicators which are widely used and have empirically shown to adequately capture socioeconomic status among the various poverty sub-groups.

Wide applicability:

- ☐ literacy
- ☐ level of education
- ☐ place of residence
- ☐ work status
- ☐ type of employment
- ☐ occupation
- ☐ type of work
- ☐ schooling of children
- ☐ income (household, individual)
- ☐ food availability
- ☐ food vs non-food expenditures

Context specific:

- ☐ housing tenure (rented, owned)
- ☐ housing conditions (type of building, materials, crowding)
- ☐ household amenities (water, toilet, electricity)
- ☐ assets, i.e. electric appliances, furniture
- ☐ health insurance
- ☐ land ownership
- ☐ productive assets
- ☐ school-aged children working
- ☐ adult man in household
- ☐ occupation of household head
- ☐ clothing
- ☐ social involvement
- ☐ cooking fuel source

Conclusion

The above provides list of widely used se indicators can serve as a checklist for questionnaires stratifying patients according to socioeconomic status. The interpretation of these indicators is always context-specific, though there are indicators used globally and are proven and popular measures to assess socioeconomic status. The literature sources below provide ample information on the correct interpretation and usage of socioeconomic indicators and can guide the adaptation of the generic questionnaire to local circumstances and the interpretation of survey results.

Income

It is useful to include measures of household consumption on food and non-food items as well as individual and household reported income for reasons given above. Asset-based approaches are useful tools, but context specific. If assets are included, questionnaires need to be adapted to local circumstances. This can be done by using data/questions from World Bank LSMS studies. Information on

assets can be collected through interviews away from the patient's home, but it is better to assess them directly in the patient's home.

Sources:

Ahmed (2005). How do you measure the concept of poverty? BRAC Experiences. Presentation of the BRAC Research and Evaluation Division. Presented at Bellagio Conference 5-8 December 2005.

Falkingham & Namazie (2001). Identifying the poor: a critical review of alternative approaches. DFID commissioned paper. London School of Economics.

Falkingham & Namazie (2002). Measuring health and poverty: a review of approaches to identifying the poor. DFID.

Galobardes et al (2006). Indicators of socioeconomic position part 1. Journal of Epidemology and Community Health 60, 7-12.

Galobardes et al (2006). Indicators of socioeconomic position part 2. Journal of Epidemology and Community Health 60, 95-101.

Morris et al (2000). Validity of rapid estimates of household wealth and income for health surveys in rural Africa. Journal of Epidemology and Community Health 54, 381-387.

Nhlema et al (2007). Developing a socio-economic measure to monitor access to tuberculosis services in urban Lilongwe, Malawi. Int J tuberc Lung Dis 11(1): 65-71.

World Bank Institute (2007). Analyzing Equity using Household Survey Data. A guide to Techniques and their Implementation. Available under http://siteresources.worldbank.org/INTPAH/Resources/Publications/459843-1195594469249/HealthEquityFINAL.pdf

back to index

Tool to Estimate Patients' Costs

Indicators to be measured with questionnaire

Please refer to the guidelines on interpretation to see which questions cover each indicator. We recommend to analyze for the median and mean (if applicable).

1. **Direct costs to patient before and during diagnosis**
   - Direct costs before and during TB diagnosis
   - The type of provider that was consulted before the patient reached the public facility

2. **Patient and Health System Delays**
   - Patient delay (time gap between onset of symptoms and first visit at public clinic)
   - Diagnostic delay (time gap between onset of symptoms and diagnosis by the NTP)
   - Health system delay (doctor delay and treatment delay)
   - Total delay (patient + health system)
   - The type of provider consulted before patient reached public facility
   - Health-seeking behavior of patient

3. **Indirect costs before & during diagnosis**
   - Indirect costs before and during TB diagnosis

4. **Direct costs of patients during treatment**
   - Direct costs during TB treatment
   - Total direct costs due to TB
   - Costs of hospitalization for TB patient

5. **Indirect costs during treatment**
   - Indirect costs during TB treatment

6. **Total costs of TB patients**
   - Total direct costs of TB patients (pre-diagnostic, diagnostic, treatment)
   - Total indirect costs of TB patients (pre-diagnostic, diagnostic, treatment)
   - Total costs of TB patients (indirect + direct before diagnosis, during diagnosis, during treatment)
   - Cost of TB including pain and suffering (willingness to pay)

7. **Productivity**
   - % reduction of productivity due to TB

8. **Coping costs**
   - % of patients who take out loan
   - Costs due to interest on loan
   - % of patients who sell assets
   - Type of assets sold
   - % reduction of household income spent on food due to TB
   - Extent of reduction in food consumption
   - % of patients whose children miss school to help finance costs due to TB

9. **Guardian costs**
   - Direct costs of guardians
   - Indirect costs of guardians
   - Total costs of guardians

back to index

10. Additional healthcare costs (including HIV)
- Additional costs due to other diseases

11. Willingness and ability to pay
- Cost of TB including pain and suffering

12. Income and affordability of TB and healthcare
- % of household income spent on food
- % of household income spent on TB (pre-diagnostic and diagnostic costs)
- % of household income spent on TB treatment
- % of household income spent on TB (prediagnostic, diagnostic, treatment)

- % of per capita income spent on TB pre-diagnostic and diagnostic costs
- % of per capita income spent on TB treatment
- % of per capita income spent on TB (pre-diagnostic, diagnostic, treatment)

- % of household income contributed by TB patient
- % reduction of household income due to TB illness of household member
- % reduction of personal income due to TB

13. Health Insurance
- % of patients covered by any kind of health insurance
- % of costs due to TB reimbursed by health insurance

14. Gender / social costs of TB
- % of women who cannot seek care by themselves
- % of women who are financially independent
- % of TB patients whose private or social life was affected by TB
- Type of effect on private or social life
- % of patients where daughters don't attend school regularly due to TB case in family
- % of patients where daughters replaced work due to TB case in family
- Difference in direct costs between men and women
- Difference in indirect costs between men and women
- Difference in patient delays between men and women
- Difference in health system delays between men and women
- Difference in reduction of productivity due to TB between men and women
- Difference in reduction of personal income due to TB between men and women

15. Socioeconomic questions
- % of literate and illiterate patients
- Educational level of patients, head of household, spouse of head of household, primary income earner
- Type of Occupation of patients
- Type of employment status of patients
- Level of impoverishment of household
- % of patients belonging to lowest income quartile/quintile of country
- % of patients belonging to poorest socioeconomic group
- % of patients belonging to minority (tribe/ethnic group/religion)

back to index

Tool to estimate patients' costs

Questionnaire

Questionnaire Number:

Patient registration Number :

Date of Interview (dd/mm/yy)	Name of Province	Name of District	Place of interview (household / facility name)	Interviewer Name
**Category of Facility**	1. Dispensary 2. Health Centre 3. District Hospital 4. Mission Hospital			

Introduction to the patient:
My name is (name). The organization I am working for, (name of organization), is interested in the costs that people face when they are seeking health care. Therefore, we would like to inquire how much people spend on healthcare, and more specifically on Tuberculosis before and during diagnosis and during treatment.

It is important for you to understand that your participation in this study is completely voluntary. We would be really grateful if you would agree to participate in this study, but do feel free to refuse. If you refuse, there will be no consequence for you and you will receive whatever care and treatment you need at the health facility as usual. If you decline to participate you will not lose any benefit that you are entitled to such as receiving care and support that is provided at the clinic.

If you choose to participate in this study you need to know that you may withdraw from the study at any stage without giving any explanation for your withdrawal. Your answers will be kept confidential. At some point I will ask you about your personal income and the income of your household. We will NOT provide this information to any tax or welfare authorities, also not after the end of the study.
This survey will take ca 30 minutes.

Do you have any questions? Do you want to participate? (circle) **Yes / No**
If Yes: Thank you!
If No: Is there a reason why not?

1. *Language* not good enough 2. Time constraint 3. Not comfortable 4. Unspecified

Patient Information *(to be filled in by Interviewer with the help of patient card; fill in also if interview is refused for non-response analysis)*	
**1. Gender** 1. male 2. female	**Age of patient:**
**2. Type of TB** (circle)	1. pulmonary smear + 2. pulmonary smear - 3. Extra-pulmonary
**3. Total duration of planned treatment** (circle)	1. (6 months) 2. (8 months) 3. Other
**4. Treatment Regimen** (circle)	1. Cat I (new Pulmonary) 2. Cat II (retreatment) 3. Cat III (new ss- or Extra-Pulmonary) 4. Cat IV (chronic, MDR)
**5. Currently in intensive or continuation phase?**	1. Intensive 2. Continuation
**6. Interviewee**	1. Same as patient 2. DOT supporter / guardian 3. Other
**7. HIV status** (only if indicated on card!)	1. positive 2. negative 3. not tested 4. unknown 5. declined

Proceed according to inclusion/exclusion criteria previously defined!

back to index

**8. Date of Investigation** (first sputum or xray dd/mm/yy)	**Date of starting Treatment** (dd/mm/yy)

Minimum treatment duration should be defined – proceed if in agreement with inclusion criteria.

Previous Treatment	
**9. a) Have you ever had TB treatment before?**<br>*Cross-check with patient card; If No, go to 10.*	1. Yes (mm/yy treatment ended) ……. ……. 2. No
**b)** If yes: **Have you completed your previous TB treatment?**	1. Yes 2. No
**c)** If No: **why not?**<br>1. Lack of money for treatment costs 2. Drug side effects 3. Moved 4. distance to facility<br>5. Other (specify):	

Delay, Prediagnostic & Diagnostic Costs

10. What symptoms did you experience that led you to seek treatment for your current illness? How long did you experience these symptoms before you went to seek treatment?

1. Cough yes ☐ no ☐________ months 2. Night sweats yes ☐ no ☐ ________ months

3. Coughing up blood yes ☐ no ☐________ months 4. Weight loss yes ☐ no ☐ ________ months

5. Other (specify) yes ☐ no ☐ ________ months

11. Did you seek treatment or advice for these symptoms at any of the following? *Check all that apply*
Where did you go first? *Circle first place of treatment*

1. District hospital yes ☐ no ☐ 2. Dispensary yes ☐ no ☐ 3. Health Centre yes ☐ no ☐

4. Mission hospital yes ☐ no ☐ 5. Pharmacy, drug & grocery store yes ☐ no ☐

6. Herbalist yes ☐ no ☐ 7. Private hospital/clinic yes ☐ no ☐ 8. Other (specify): ☐________

b) Have you visited a traditional healer? yes ☐ no ☐

If other than public provider was chosen in 11):

12. Why did you not go to the public health facility, such as government clinic or hospital when you first realized you were sick? *Circle most applicable.*

1. distance to facility 2. too expensive 3. time consuming to wait 4. lack of available facilities

5. mistrust of government health services provision 6. Belief system 7. No drugs available

8. other (specify)

**13. How far is the nearest government facility for**			
**a) diagnosis and treatment**	____ hours walking	______ hours with transport	other:__________
**b) treatment only**	____ hours walking	______ hours with transport	other:__________

back to index

14. About how much did you spend for each of these visits before you were diagnosed with TB, including the visit when you actually received your diagnosis?
For all that don't apply, mark N/A; Fill one line per visit

	**Provider** (copy from question xy providers where patient sought treatment or advice)	**Total Time spent per visit** (in hours, includes travel time)	**Administrat ive Costs** (consultative , registration)	**Test costs** (for sputum or other except xray)	**Xray costs (**includes sending xrays to radiologist, travel & fees)	**Drug costs** (all kinds total)	**Travel Costs** (return total)	**Food costs** (total)	**Accomo-dation Costs** (total)	**Sub-Total costs per visit**	**Insurance Reimburse-ment** *If yes: amount, if no n/a*
**Visit 1**											
**Visit 2**											
**Visit 3**											
**Visit 4**											
**Visit 5**											
**Visit 6**											
**Visit 7**											
**Visit 8**											
**Visit 9**											
**TOTAL**	---------------------------------------										

**Total Direct Prediagnostic & Diagnostic costs (sum sub-totals) minus insurance =**	***Name of currency***

back to index

Treatment Costs

Costs related to DOT

15. Where do you currently take your TB drugs?
If the patient has visited two different DOT places, tick the current place and report costs only for that place.
If DOT at home, go to 19.

1. Health facility / hospital   2. Home   3. Community   4. Workplace   5. Dispensary

15 b) How many times per week do you go there place to take your drugs? ☐ 3 times ☐ 5 times ☐ 6 times ☐other

**16. How long does it take you to get there** (one way)	____ hours walking ______ hours with transport other:__________
**17. How long does one of these visits take on average, including time on the road and waiting time** (total turnaround time)**?**	Hours
**18. From your home to the DOT place, how much does it cost if you take transport?** (both ways)	
**19. How much do you spend on food on the road, while waiting, for lunch?**	

Costs related to picking up the TB drugs – where drugs are currently picked up

**20. How often do you travel to the health facility / hospital for picking up your TB drugs?**	Times / month
**21. How long does it take you to get there** (one way)	____ hours walking _____ hours with transport ______ other
**22. How long does one of these visits take on average, including time on the road and waiting time** (total turnaround time) ?	hours
**23. From your home to the facility, how much does it cost if you take transport?** (both ways)	
**24. If you go to a facility to pick up your drugs, how much do you spend on food on that day?** (on the road, while waiting, lunch etc.)	
**25. a) Do you have to pay administration fees when picking up your TB drugs?** *If No, go to 26.* **b)** If YES, **how much?**	1. Yes 2. No
**26. a) Do you have any accommodation costs when picking up your TB drugs?** *If No, go to 27.* **b)** If YES**: how much?**	1. Yes 2. No

Costs related to follow up tests

**27. a) Did you ever have to go to the health facility in addition to your regular visits for follow up tests since the beginning of treatment?** *If No, go to 28.*	1. Yes 2. No
**b) If yes, how many times?**	Times
**c) If yes, did you have to pay any additional costs any time during the entire period?**	1. Yes 2. No
**d) If so, what kind of costs and how much?** Fees_______ sputum test ________ Xray______ TB Drugs _______ Other Drugs________ Other_________	Total:
**e) How long does one of these follow-up visits take on average, including time on the road, waiting time and tests** (total turnaround time) **?**	Hours

back to index

Guardian Costs	
**28. a) Does any family/friend/DOT supporter accompany you on any visits or go in your place to collect your TB drugs** ? *If No, go to 29.*	1. Yes 2. No
**b)** *If YES,* **on how many visits has your family/friend/DOT supporter accompanied you or gone in your place?** *Record pre-diagnosis/diagnosis visits and treatment visits separately*<br><br>Complete at data entry:<br><br>Pre-diagnosis/diagnosis costs per visit: Transport ______ Food ______ Accommodation ____<br><br>Costs during treatment per visit: Transport ______ Food ______ Accommodation __________	_____ Diag. times<br><br>______Treatment times<br><br>Total Diag:<br><br>Total Treatm:
**c) How much does your friend/family/DOT supporter earn per day?**	1.<br><br>2. Doesn't earn
**d) Why did someone accompany you?**<br><br>1. Distance 2. Security 3. Administrative barriers 4. Too ill to travel alone<br><br>5. Was required for treatment 6. Other (specify)	

Hospitalization	
**29. Have you been hospitalized before or during your TB treatment?**<br>*If No, go to question 38.*	1. Yes 2. No
**30.** If YES: **how many days in total did you stay at the hospital?**	days
**31. How much did you pay in the hospital during your entire stay?**<br><br>Hospital administration fees: Sheets/Linnen:<br><br>Food (not provided by hospital): Transport (return):<br><br>Drugs: Tests: Others:	Total:
**32. Did any family/friend stay with you while in hospital?** *If No, go to question 38.*	1. Yes 2. No
**33.** If YES**: How many days did he/she stay with you (sleep there)?**<br><br>**34. Were there any extra costs for your relative/friend for staying at the hospital?**<br><br>Accommodation (hospital or other): Food:<br><br>Transport: Other:	Days<br><br>1. Yes 2. No<br><br>Total Costs:
**35. How much does your friend/family normally earn per day?**	1.<br><br>2. Doesn't earn
**36. a) Did any other family/friend visit you while in hospital?** *If No, go to 38.*<br><br>**b) If yes, how many people visited you?**<br><br>**c) how many times did they visit you?**<br><br>Accommodation per person: Food per person:<br><br>Transport per person: Other:<br><br>**37. How long were the visits including traveling time?**	1. Yes 2. No<br><br>Persons<br>Times<br><br>Total number of visits:<br><br>Total Cost per person:<br><br>hours

back to index

Other Costs *Food Supplements*	
**38. a) Do you buy any supplements for your diet because of the TB illness, for example vitamins, meat, energy drinks, soft drinks, fruits or medicines?** *If No, go to 39.*	1. Yes 2. No
**b)** If YES: **What kind of items?** (specify)<br><br>1. Fruits 2. Drinks 3. Vitamins/Herbs 4. Meat 5. Other (specify):	
**c) How much did you spend on these items in the last month approximately?**	

Other Illnesses	
**39. a) Do you have any chronic illness for which you are receiving treatment?**<br>*If No, go to 40.*<br><br>**b)** If yes: **which?**	1. Yes 2. No
**c) Are there any additional costs for you because of this other illness <u>besides</u> the costs that you have already mentioned?** *If No, go to 40.*	1. Yes 2. No
**d)** If YES: **How much are these additional costs on average per month?**<br><br>Tests: Drugs: Transport: Food:<br><br>Other:	Total:
**40. How much did you spend on healthcare on average per month BEFORE the TB illness?**	
**41. How much do you spend on healthcare on average per month NOW?**	

Insurance	
**42. a) Do you have any kind of private or government health/medical insurance scheme?**<br>*If No, go to 43.*	1. Yes 2. No
**b)** If YES: **What type?** 1. reimbursement scheme 2. monthly medical allowance 3. donor<br><br>4. family/community fund 5. Western scheme (contract) 6. Other (specify)	
**c) Have you received reimbursement for any costs related to the TB illness?**<br>*Cross-check with question xy (table on prediagnostic & diagnostic costs) If No, go to 43.*	1. Yes 2. No
**d) How much have you received as reimbursement?**	

Coping Costs	
**43. Did you borrow any money to cover costs due to the TB illness?**<br>*If No, go to question 45.*	1. Yes 2. No
**44. a)** If YES: **How much did you borrow?**<br><br>**b) From whom did you borrow?** *Circle most appropriate*<br><br>1. Family 2. Neighbors/friends 3. Private bank 4. Cooperative<br><br>5. Other (specify):	
**c) What is the interest rate on the loan? (%)**<br><br>1. less than xx 2. xx to xy 3. More than xy 4. I don’t pay any interest	

back to index

5. I am not expected to pay back the money

**45. a) Have you sold any of your property to finance the cost of the TB illness?** *If No, go to 46.*	1. Yes 2. No
**b)** If YES**: What did you sell?** *Circle most appropriate*<br>1. Land 2. Livestock 3. Transport/vehicle 4. Household item 5. Farm produce<br>6. Other (specify):	
**c) What is the estimated market value of the property you sold?**	
**d) How much did you earn from the sale of your property?**	

Socioeconomic Information *Individual Situation and Income*

46. Who is the primary income earner in the household? *Circle most appropriate*

1. Patient 2. Wife/mother 3. Husband/father 4. Extended family 5. Son/daughter

6. Other (specify)

What is the highest level of education of ...?

47. The patient?

1. Not attended/illiterate 2. primary 3. secondary 4.graduate/certificate 5. other

48. Primary income earner?

1. Not attended/illiterate 2. primary 3. secondary 4.graduate/certificate 5. other

49. Head of household?

1. Not attended/illiterate 2. primary 3. secondary 4.graduate/certificate 5. primary income earner = head of hh

50. Spouse of head of household? *If more than one spouse, choose highest level of education*

1. Not attended/illiterate 2. primary 3. secondary 4. graduate/certificate 5. other

**51. Are you currently formally employed?**<br>*Name all options first*	1. Yes, formal work *(go to 54)* 7. Combination (specify)<br>2. No, informal work *(go to 54)*<br>3. On sick leave *(go to 52)* 8. Other (specify)<br>4. Retired *(go to 52)*<br>5. School, university *(go to 58)*<br>6. Housework *(go to 54)*

**52. Is the reason for Not Working related to the TB illness?**	1. Yes 2. No
**53.** If Yes**: When was the last time you were working? (mm/yy)**	

54. How are you usually paid?

1. cash 2. in kind 3. cash and in kind 4. not paid 5. bank transferred salary 6. other

55. What was your estimated personal take home earning per month BEFORE the TB illness? (includes welfare, disability, or other social support):

1. Under xx per week 2. Xx to xy per week 3. Xy to xz per week 4. More than xz per week 5. Don't earn

**56. What is your estimated personal take home earning per month NOW**? (includes welfare, disability, or other social support)<br><br>1. Under xx per week 2. Xx to xy per week 3. Xy to xz per week 4. More than xz per week 5. Don't earn	
If answer to 56 differs from 55:<br>**57. Is the change related to the TB illness?**	1. Yes 2. No

**58. a) Have you ever stopped working/going to school/doing housework due to TB?**<br>*If No, go to 59.*	1. Yes 2. No
**b)** If YES**: for how long?**	1. Less than 1 month 2. one month 3. 2-3 months 4. 4-5 months 5. more than 6 months
**59. a) Does someone stay home <u>specifically</u> to take care of you?**<br>*If NO, go to 60*	1. Yes 2. No
**b**) If YES: **for how long?**	Weeks
**c) Did they quit their income-earning job to stay home and care for you?**	1. Yes 2. No
**60. How regularly did you work before you became ill with TB?**	1. Throughout the year 2. Seasonal/part of the year 3. Day labor 4. Other
**61. Did you have to change jobs when you became ill with TB?**	1. Yes 2. No
**62. What is your main occupation?** *Tick all that applies, cross-check with question 51.*<br><br>1. Sales/Service 2. Agriculture 3. Household 4. Production/construction<br><br>5. Combination (specify) 6.Other (specify)	
**63. How many hours did you work on average per day BEFORE you became ill with TB?**	Hours
**64. How many hours do you work on average NOW per day?**	Hours
If answer to 64 differs from answer to 63:<br>**65. Is the change related to the TB illness?**	1. Yes 2. No
If answer to 64 differs from answer to 63:<br>**66. a) Is someone doing the work that you used to do?**<br><br>**b)** 1. daughter 2. son 3. spouse 4. friend 5. nobody 6. other family	
**67. a) Do you have children of or below school age?** *If No, go to 68.*	1. Yes 2. No
**b) Do all of your children of school age attend school regularly?** *If YES, go to 67d)*	1. Yes 2. No
**c)** If NO: **Why not?** *Circle most appropriate*<br><br>1. Needs to help around the house 2. No money for school fees 3. Also sick 4. Has to work to earn income<br><br>5. Other (specify):	
**d) Do any of your children of or below school age work to finance costs due to the TB illness?**	1. Yes 2. No
**68. If you employed someone to do the housework for your household, how much would you have to pay him/her per day?**<br><br>**a) While you are sick**	

back to index

**b) While you are healthy**	
**69. Are you financially independent?**	1. Yes 2. No

**70. a) Has the TB illness affected your social or private life in any way?** *If No, go to 71.*<br>1. No 2. Divorce 3. Loss of Job 4. Dropped out of school 5. Separated from spouse/partner<br>6. disruption of sexual life 7. Sick child 8. Other (specify):	
**b)** If Yes: **Has this resulted in a financial burden?**	1. Yes 2. No
**71.** ***What is your tribe / ethnic group / religion?***	1. 2. 3. 4. 5.

Household Income and Spending

72. How much do you estimate was the average income of your household per month BEFORE the TB illness ? (for all persons in the house, including patient; includes welfare payments, government assistance or other social support)

1. income patient: 2. income rest of household 3. welfare payments

4. government assistance 5. Other: TOTAL:

73. How much do you estimate is the average income of your household per month NOW ?

1. income patient: 2. income rest of household 3. welfare payments

4. government assistance 5. Other: TOTAL:

**74. How many people regularly sleep in your house?** (including patient)<br>*If patient lives alone, go to question 77 and replace the word 'household' with 'you'*	
**75. How many of the household members are paid for working?** (including patient)<br>(*includes payment in kind or farm produce)*	
**76. a) Besides yourself, does anyone else of your household receive treatment for TB?**<br>*If No, go to 77.*	1. Yes 2. No
**b)** If Yes: **How many?**	
**77. How much food did your household consume every month on average BEFORE the TB illness?**<br>*Calculate value*<br><br>*If home production:*<br>**If the food that you consumed per month before the TB illness was sold on the market: How much would it be worth? (plus how much you spent on average on food not produced at home?)**	
**78. How much food does your household consume NOW every month on average?** *Calculate value (for same number of people)*<br><br>*If home production:*<br>**If the food that you consume per month now was sold on the market: How much would it be worth? (plus how much you spent on average on food not produced at home?)**	
**79.** *If answer to 78 differs from 77:* **Has the amount of food consumed per month changed due to the TB illness?**	1. Yes 2.No

back to index

Socioeconomic Indicators	
**80. What is your electricity supply?**	1. Own connection 2. Shared connection 3. None
**81. What is your source of drinking water?**<br>1. Rainwater 2. lake/pond/ dam/river 3. public well 4. private well/bore hole 5. piped water 6. bottled water	
**82. What type of toilet facility is available?**<br>1. no facility/bush/field 2. shared pit toilet/latrine 3. own pit toilet/latrine 4. flush toilet	
**83. How many rooms are there in your house?**<br>1. 1 room 2. 2 rooms 3. 3 rooms 4. 4 or more rooms	
**84. Current place of residence?**	1.Urban 2. Urban Slum 3. Rural 4. Other (specify)
**85. Do you own the house or residence you live in?**	1. Yes 2. No
**86. Do you own….**<br>*Include standard assets adapted to country Demographic and Health Survey (DHS)*<br>1. mobile phone<br>2. washing machine<br>3. motorcycle<br>4. bicycle<br>5. land (quantify)<br>6. etc…	
**87. If the government could provide you with some service to ease the burden of TB on you and your household, what would you prefer to have?** *State options, choose one*<br>1. Transport vouchers 2. food vouchers 3. More efficient service 4. Other (specify):	

We would like to know the cost of the TB illness on the welfare of your household; that is, we would like to put a value on the TB illness which includes pain and suffering.

Therefore, we would like to know how much it would be worth to you if you could avoid becoming ill with TB in the first place. Note that we don't ask what you actually can, but what you would be willing pay if you had an unlimited amount of money.

**88. How much would you be willing to pay for not becoming ill with TB in the first place?**
1. Under xx 2. between xx and xy 3. over y 4. Other (specify)

Thank you for your cooperation! Is there anything you would like to ask or say?

Comments by Interviewer:

Date, Signature by Interviewer:

back to index

Tool to Estimate Patients' Costs

Guidelines for Adaptation of Questionnaire to local circumstances

Contents

Language
Pre-testing
Specific Questions to be adapted

1. Language

The validity of results is heavily affected by the answers given in an interview. The language used is an important component to guarantee that the questions are properly understood and adequately answered. This is especially the case when interviewing vulnerable populations such as ethnic minorities, the very poor, migrants, and refugees. Unless the population to be interviewed is native English speaking, the questionnaire needs to be translated as there is evidence that the reliability of questions is greater when they are administered in the respondent's mother tongue, even if the respondent is multilingual. The translation can be written underneath the English questions or a separate questionnaire can be designed.

The translation is best done by someone who understands the objective of the questionnaire, the intent of the questions and who speaks both languages fluently. In order to check the validity of the translation, it is recommended to translate the questionnaire back into English by someone who has not seen the original version, and is not familiar with the background context of the questionnaire. The back-translated version is then compared with the original one and differences in meaning need to be adjusted. Time-permitting, it is always recommended to have a bilingual peer compare both versions and evaluate the questions according to content, meaning and clarity of expression.

Another way of ensuring cross-validity is to interview a set of respondents in English and another set in the local language. Their answers are then compared to detect differences in understanding.

It is important that the language used in the questionnaire corresponds to the daily spoken language of the patient – rather than the high-end, sophisticated language used by academics. The translated questionnaire should therefore be pretested.

2. Pre-testing

The objectives of pre-testing are to identify questions that are poorly understood, ambiguous or evoke hostile or other undesirable responses. The already-translated questionnaire should be used for pre-testing. A pretest should answer the following questions:

- □ Are all words understood?
- □ Are the questions interpreted similarly by all respondents?
- □ Do the closed-ended questions have answers that are applicable to each respondent?
- □ Do some questions evoke answers that can't be interpreted?

Steps in Pre-testing:

1. Obtain peer evaluation of draft questionnaire
2. Test the revised questionnaire on friends, colleagues etc

3. Prepare instructions and train interviewers for pilot test
4. Pre-test the questionnaire on a sample of respondents (ca. 10-50)
5. Obtain comments from interviewers and subjects; review pre-test responses to check for potential misunderstandings
6. Revise questions that cause difficulty
7. (Pretest again – recommended if time permits)
8. (Revise again)
9. Prepare revised instructions and train interviewers for implementation of full data collection
10. Monitor performance of the questionnaire during early phase of study

3. Specific questions to be adapted

Pre-Diagnostic and Diagnostic Costs Section

Where did you go first? *Check all that apply, includes current clinic*

- Adapt the places where help is sought to local circumstances, adding the names of facilities. Depending on how far the patient is into treatment, it will be more or less difficult to recall items from the past. Attitudes towards the passage of time differ tremendously by country and culture. Instead of weeks, some might think in agricultural seasons or weather seasons (rainy season, dry season) or other time references, such as the moon calendar. The interviewer should help the patients to recall items by prompting, i.e. "Was it before Bayram, or after?"

- Interviewers should have a clear understanding of how to record costs in the questionnaire, as some costs will be recurring (e.g. transport) whereas others will happen less regularly (e.g. x-ray or sputum tests).

How much did you spend for each of these visits? *Fill one line per visit or group several visits to one type into one line*

- In order to record the costs correctly and coherently throughout the survey, the interviewers need to be instructed explicitly about the definitions of the types of costs, what is meant with cost of food, cost of travel and cost of accommodation and how they can help patients recalling items by prompting.

If other than public provider was chosen: **Why did you not go to the public health facility, such as government clinic or hospital when you first realized you were sick?**

- This question investigates the reasons behind the specific health-seeking behavior of the patient. This is usually of high interest to the program, but the WHY question actually does not yield additional information regarding costs to TB patients.

Treatment Costs Section

Where do you take your TB drugs? *Go to the appropriate section (DOT at health facility, home, community, workplace). If DOT at health facility / hospital; specify type of health facility:*

- The types of DOT differ from program to program. While the most common DOT schemes are listed here already, this section needs to be adapted to the specific DOT schemes in the country/region.

Why did someone accompany you?

- This is a gender-sensitive WHY question which does not yield additional information regarding costs to TB patients.

Other Costs Section

Do you have any kind of private or government health/medical insurance scheme? If YES: **What type?**

- These questions need to be adapted according to the insurance/funding schemes that are available in the respective country or region.

Do you have any chronic illness for which you are receiving treatment? If yes: **which?**
Are there any additional costs for you because of this other illness besides the costs that you have already mentioned? If YES: **How much are these additional costs on average per month?**

- This question should elicit costs for any other wide-spread illnesses such as malaria, HIV/AIDS, and diarrhea allowing us to later compare TB costs to other illnesses costs. It may be useful to assess these additional costs by category of disease, rather than as aggregate additional illness.

- Depending on the program practice in the country, it may or may not be possible ask directly for the patient's HIV status. Local privacy policies should be respected If people are likely to say No when asked whether they are co-infected, although they are, it does not make sense to ask for additional costs.

Socioeconomic Information Section

Are you currently formally employed?
When was the last time you were working?
Is the reason for Not Working related to the TB illness?
How regularly did you work before you became ill with TB?
What is your main occupation?
How are you usually paid?

- These are questions to derive more information regarding socioeconomic status, and may be problematic in areas with extensive subsistence agriculture, high unemployment or a large informal sector. Their value to the questionnaire should be tested to decide whether to include them or not.

Are you currently formally employed?
What is your main occupation?
Current place of residence?

- The suggested answers to these questions have to be adapted to current practices in the country/region. If not known, consult a recent Demographic and Health Survey (available at national statistics office) or a Living Standards Measurement Survey/Questionnaire (available under http://www.worldbank.org/LSMS/guide/select.html)
- It may be useful to subdivide occupational categories into formal, informal and subsistence groupings for analysis.

What is the highest level of education for ...?
Primary income earner?
Head of household?
Spouse of head of household?

- Adapt the pre-defined answers to the question according to local schooling system.

back to index

Household Income and Spending Section

How much do you estimate was the average income of your household per month BEFORE TB?
How much do you estimate is the average income of your household per month NOW ?

- For these questions it is suggested to provide different ranges for the respondent to pick from, to avoid social desirability bias and to make the question less threatening to the respondent. These ranges have to be adapted to local levels. If not known, results from Living Standards Measurement Surveys can be used (available under http://www.worldbank.org/LSMS/guide/select.html) or alternatively the ranges given by UNDP Human Development Reports can be used (available at http://hdr.undp.org/en/reports/ choose Theme 'Income and economic growth'). It is important that all questions asking for any kind of expenditure should be recorded in the same currency and with regard to the timeframe listed in the question throughout the questionnaire!

- Ensure that the question is posed to uncover the total income of the household, not limited to the income available to the patient. For those survey areas where cash income is less likely, develop a list of prompt questions that interviewers can use to quantify less cash-based incomes.

Socioeconomic Indicators Section

What is your electricity supply?
What is your source of drinking water?
What type of toilet facility is available?
How many rooms are there in your house?

- These aspects of housing amenities are popular, but they are only a suggestion. These questions might not give information about socioeconomic situation of the respondent. For example, if almost everybody has an electricity supply, it doesn't make sense to ask for it. Additional questions could ask about housing tenure (rented, owned), housing conditions (type of building, materials, crowding), assets (electric appliances, furniture, bicycle etc), cooking fuel source, land ownership, productive assets.

- Which housing indicators are used depends on the type of housing present in the region, materials used, and customs. The applicability of these questions can be tested by regression analysis.

What is your tribe/ethnic group/religion?

- This question has been left open (and therefore in italics), because it is aimed to distinguish minorities and vulnerable populations from majority populations. This depends on the prevalence of minorities and ethnic groups in the country. For example, in Sri Lanka it is useful to know whether the respondent is Sinhala or Tamil, in the Philippines it would be useful to ask whether the respondent is Christian, Muslim or Buddhist.

- This question has been deliberately put at the end of the questionnaire, for ethnicity and religion are always sensitive subjects and asking it in the beginning might influence the way how subsequent questions are answered.

How much food did your household consume every month on average BEFORE the TB illness?
If home production: **If the food that you consumed per month before the TB illness was sold on the market: How much would it be worth?** (plus how much you spent on average on food not produced at home?)

How much food does your household consume NOW every month on average?

If home production: **If the food that you consume per month now was sold on the market: How much would it be worth? (plus how much you spent on average on food not produced at home?)**

If answer to xy differs from xy: **Has the amount of food consumed per month changed due to the TB illness?**

- Since income is difficult to measure, it is often preferred to measure consumption as a proxy for income. The best way is to distinguish between food and non-food items. This also lets us calculate the % of food costs of total costs, which also gives an idea about the social welfare of the household.
- In this questionnaire, the question on non-food item consumption is not included, because it takes at least ½ hour to estimate all costs that occur in a month on an item by item basis and it is heavily dependent on the type of expenditures that residents in a certain country have. If enough time is available, it should be considered to include a question on non-food expenditures with a list of items derived from the last Demographic and Health Survey.
- As an alternative approach, basic foodstuffs used but not produced by most households could be investigated (such as sugar, cooking oil and flour) for household expenditure proxy. Attention should be paid to inflation in food staple prices and accounted for in data interpretation.

References

Armstrong, White & Saracci (1994). *Pinciples of Exposure Measurement in Epidemiology*. Oxford: Oxford UP.

Morris et al (2000). Validity of rapid estimates of household wealth and income for health surveys in rural Africa. Journal of Epidemology and Community Health 54, 381-387.

World Bank Institute (2007). *Analyzing Equity using Household Survey Data. A guide to Techniques and their Implementation*, Chapters 2 and 6 http://siteresources.worldbank.org/INTPAH/Resources/Publications/459843-1195594469249/HealthEquityFINAL.pdf

back to index

Tool to Estimate Patients' Costs

Methodology, Sampling, Interviewer Training

Contents
Methodology
Sampling
Survey Examples
Coding
Interviewer Training Issues

1. Methodology

The general procedures in survey research require the following steps, which we will apply to the Tool to Estimate Patients' Costs:

1) Decide on research question and goals of the study

The research question here is: What are the costs for TB patients before / during diagnosis and during treatment? The answer to this question will then also give us an idea whether TB services are affordable to patients.

2) Decide on the population relevant to the study

The survey aims to have a close look at the poor affected by TB. Since TB patients are usually found among the low-income groups, the population should contain a larger proportion of poor people than what is found in the overall population of a country. Inclusion and exclusion criteria need to be decided upon, such as whether only new patients, re-treatment, MDR or a mixture shall be studied; whether specifically defaulters are of interest; whether unregistered patients shall be included or patients who also/only visit private sector facilities, etc.

3) Decide on method used to collect the data

This will be done through administrating a questionnaire in face to face interviews (see Guidelines for Adaptation).

4) Write research protocol and submit to ethics committee for ethical clearance

5) Develop a sampling strategy

This section on methodology will be followed by a section on sampling.

6) Adapt questionnaire and codes

See the Guidelines for Translating and Adapting the provided questionnaire.

7) Conduct interviews

8) Enter and analyze data

We recommend to analyze **median** and mean values for costs.

You can use any statistical package. We used **Epi Info**, which can be downloaded for free on the CDC website.
We have prepared a data entry template with the questions of the questionnaire for Epi Info. Epi Info will be the mechanism that stores and analyzes your data and subsequent data set, so it is crucial that the template is suitable for the context in which data is being collected. This template uses the generic Tool questionnaire, and there is a data entry field for each question and possible answer. Each completed questionnaire is entered into the database (using Enter Data), and saved there for cleaning and later analysis (using Analyze Data). Epi Info stores the entered data in a format that can also be used in Excel and Access. Codes for each answer are already included in the template.

To make changes to the layout of the data entry template, select 'Edit View' while in the Enter Data section of the program, or simply go to 'Make View', open the Tool template called "Kitui", and make changes directly to the template. It is recommended to first make a copy of the file and work in the copy, because Epi Info stores changes immediately and later revisions may then not be possible anymore.

It is strongly recommended that a statistician familiar with Epi Info be consulted before and during data entry with the Tool template to minimize errors. Furthermore, the **'Epi Info for Beginners' manual** is recommended to give an overview of how to use this free software: "Epi Info Beginner's Manual with Exercises" by the Great Lakes Epidemiology Center Community Based Research Training, Updated: 02/2004.

9) Report results

See the Guidelines on Interpretation of Results and Suggestions for Interventions based on Results.

2. Sampling

This survey will provide information on a subset of a population and, if appropriately selected, can provide the basis for making inferences about the population. The sample is selected from a sampling frame, which is the total of the sampling units. Advice on sampling may be sought from the national statistics office.

Points to consider when estimating sample size are:

- main indicators to be measured
- number of sites / mean costs
- hypothesized proportions
- population to be studied (new / retreatment / MDR patients / defaulters etc.)

Possible Scenario:
The Tool is used to survey only new patients with at least one month of treatment completed. TB patients will be interviewed at health facilities. The unit of analysis is the TB patient; sampling units are health facilities; secondary units are districts and primary units are provinces. The sampling frame is the TB register of a diagnostic and treatment facility. A small survey to generate baseline data without large sample sizes is conducted. One province is purposively selected. Within this province, 5 districts are sampled, and in each district there are 16 diagnostic and treatment facilities, a total of 80 facilities. If we want to interview 200 patients, 50 per facility, we need to sample 4 facilities. Statistical software programs such as SPSS or STATA can help generate a sample.

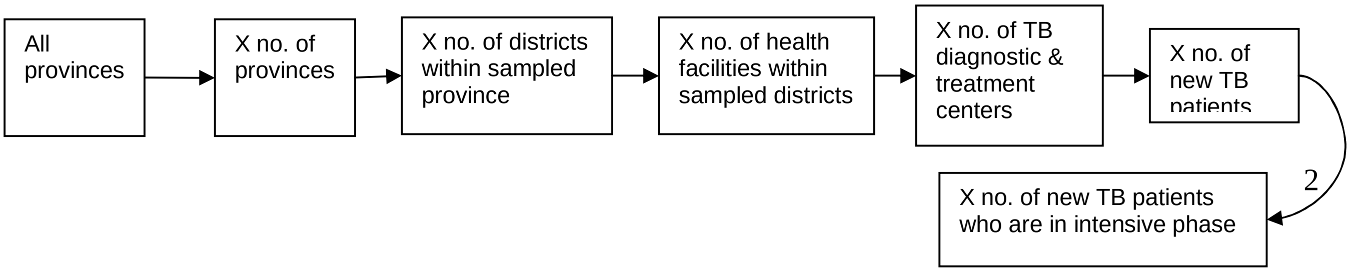

Purposive Sampling
It is conceivable to draw a purposive / convenience sample if a certain district or province is of particular interest. Inferences about the studied population are then however limited to this particular district/province. An initial decision should be made as to whether the aim of the survey is to establish a representative result/baseline or to get a basic idea about the current situation in a certain area. Representativeness of a sample size can be assessed through the total number of TB patients at a point in time (not the number of new cases!).

Patients can be sampled as they come in on clinic days, until a certain number is reached, or they can be sampled by going through the register and taking a random sample. Depending on the size of the health facility and the number of patients on treatment, the first method might be more practicable in small clinics, whereas the latter might be easier in big clinics. The type of clinic days need to be taken into account to avoid bias. For example, if those patients in intensive phase might attend the health facility on different days than those in continuation phase. Patients can be also interviewed at home, but this may introduce problems of confidentiality and social desirability bias[1]. Our questionnaire is flexible about which methods are chosen.

Non-response bias
When patients are interviewed, it is important to record non-responses to minimize bias in data interpretation, including those who refused an interview and those who cannot be interviewed because of language barriers (such as migrants or minorities).

3. Target group, inclusion and exclusion criteria

The thoughts below may help in defining inclusion and exclusion criteria <u>before</u> the questionnaire is adapted to local circumstances and according to the objectives of the survey.

- **Timing of interview:** The questionnaire is designed to interview TB patients who have completed at least one month of TB treatment. A good balance between recall bias and cost experience needs to be found.
- **Target group:** In assessing the financial burden of poor patients, the target group could be narrowed down to poor TB patients, targeted through purposively sampling poor districts. Since costs due to an HIV co-infection are to be taken into account and can be significant, the sampling of the patients may take the prevalence of HIV in the region into account. Here, purposive sampling may also be applied. The questionnaire in its generic form is tailored towards new patients, but it can be adapted to interview re-treatment and even MDR patients. It is recommended to choose only one or two of these sub-categories of TB patients, in order limit variance in the data and produce meaningful results.
- **Place of interview:** Patients can be either interviewed at the health facility or at home. Both have advantages and disadvantages. Our generic questionnaire leaves this open, as well as how far the patient has advanced into treatment.

4. Examples of small surveys on patient costs

Jackson et al (2006). *Poverty and the economic effects of TB in rural China*. Int J Tuberc Lung Dis 10(10):1104-1110.

[1] **Social desirability bias** is a term used in scientific research to describe the tendency of respondents to reply in a manner that will be viewed favorably by others. This will generally take the form of overreporting "good" behavior or underreporting "bad" behavior.

Four counties were selected on the grounds of being part of a World Bank financed project. Cases were chosen from economically productive age group (25-60), restricted to new cases until the target of 40 patients per county was reached (160 cases).

Kemp et al (2007). *Can Malawi's poor afford free tuberculosis services? Patient and household costs associated with a tuberculosis diagnosis in Lilongwe*. Bulletin of the World Health Organization 85(5), 580-587.

Nhlema Simwaka et al (2007). *Developing a socio-economic measure to monitor access to tuberculosis services in urban Lilongwe, Malawi.* Int J Tuberc Lung Dis 11(1): 65-71.

"Five health centers in urban Lilongwe were selected where there is the highest burden of TB cases in Malawi. Only patients in the intensive phase of treatment were selected above 16 years of age. At each treatment centre a list of new pulmonary TB pa[tients in the intensive phase of treatment was drawn up in chronological order and numbered. The number of patients systematically sampled from this list was proportional to the total number of TB patients in the intensive treatment phase at the centre."
"For the cross-sectional survey, a sample size of 179 TB patients was calculated based on the assumption that the poverty rate within urban Lilongwe is 38%, that the rate of poverty among TB patients is at least 15% higher than in the general population. Confidence interval 95%. Treatment registers of five urban DOT centers were used to identify every third new adult patient in the intensive phase of treatment."

Kamolratanakul et al (1999). *Economic impact of tuberculosis at the household level*. Int J tuberc Lung Dis 3(7), 596-602.

"Socio-economic data for tuberculosis patients were collected in a cross-sectional survey performed at 16 health care facilities in Thailand in 1996/97. The study was conducted at two district hospitals, one provincial hospital and one referral center within each of the country's four administrative regions. The study sites for each region were determined by random sampling. At each study site, a cluster sample of all adult tuberculosis patients who completed treatment between August 96 and February 97 was subjected to in-depth interviews using a structured questionnaire." The total sample size was 687 patients. The distribution of these 673 patients grossly corresponded to the distribution of the general population.

5. Coding

Each question in the questionnaire needs to receive a code. This code serves to give a name to the variable which is measured and will be used to identify the question during data analysis. The version of the questionnaire for the analyzer should include these codes. Following are examples of codes:

**Where do you take your medicine?**	1. Health facility / hospital<br>2. Home<br>3. Community<br>4. Workplace	**DOTHC**<br>**DOTHOME**<br>**DOTCOM**<br>**DOTWP**

back to index

**How often do you travel to the health facility / hospital for taking your medicine?** NRVISITDOT	Times / week
**How much does one of these visits cost on average?** COSTVISITDOT<br>1. transport 2. food 3. fees 4. tests: sputum: xray: other tests:<br>5. TB medicines 6. Other medicines 7. Food supplements 8.Other<br>Total:	

**Have you ever stopped working due to TB?** STOPWORKTB	1. Yes 2. No
**If yes, for how long?** TIMESTOPWORKTB	1. one month 2. 2-3 months 3. 4-5 months<br>4. more than 6

6. Training of Interviewers – important issues

a. Depending on how far the patient has progressed with treatment, it might be difficult for him/her to recall cost items. The interviewer should make it as easy as possible for the patient to recall by using local methods of time structuring; Interviewers should be given examples how to prompt responses regarding time and types of costs.

b. Interviewers need to be instructed about indicator definitions, such as types of costs, what is meant by cost of food, cost of travel and cost of accommodation, what is included and what is excluded and how they can help patients recalling items by prompting. This will help to ensure consistency in interviews and prompting by interviewers.

c. Interviewers need to be sensitized on the different phases (intensive, continuation) and types of TB treatment (hospitalization, different forms of DOT) and associated costs (sputum conversion test, follow up test, medicine collection etc.), to avoid double counting costs. It also needs to be clear to the interviewers what counts as TB drugs and what are additional drugs that are prescribed/bought.

d. Interviewers should be familiar with the concept of guardian costs; a guardian is someone who accompanies the patient to the health facility/hospital or other visits because the patient cannot go by himself. The guardian incurs direct and indirect costs. If the guardian lives in the same household as the patient, the cost to the household is much higher if guardian costs are included.

e. Difficult concepts such as willingness to pay, coping and guardian costs need to be explained in detail with clear instructions on the intent behind the questions to ensure that interviewers are able to explain these concepts to patients.

i. It may be complicated for some patients to address questions that refer to their situation “prior to their diagnosis” vs. “post diagnosis” as they may not be clear when or where their diagnosis was made. In addition there may be other big changes in their life that coincide with their illness. It is important to teach interviewers how to tease apart issues (if any) so that changes in income etc. are truly caused by their TB, and not because of some other unrelated event.

f. Interviewers should be informed about the nature of TB, what their participation means for their own health and how they can protect themselves. Depending on which kind of patients are interviewed (new, re-treatment or MDR patients), and how far the patient is into treatment, risks to

interviewer health differ. For example, patients who are in their first month of treatment might still be infectious. The interviewer needs to be aware of that and knowledgeable about infection control measures; i.e. conducting the interview outside or in a well-ventilated room.

References:

Cochran (1977). *Sampling Techniques*. New York: John Wiley & Sons.

Meier, Brudney & Bohte (2008). *Applied Statistics for Public and Nonprofit Administration*. Boston, MA: Wadsworth Publishing.

World Bank (2007). *Analyzing Health Equity using Household Survey Data*. Available under http://siteresources.worldbank.org/INTPAH/Resources/Publications/459843-1195594469249/HealthEquityFINAL.pdf

back to index

Tool to Estimate Patients' Costs

Guidelines for interpretation of results generated by questionnaire

Table of contents

1.	Direct costs to patient before and during diagnosis	2
2.	Patient and Health System Delays	2
3.	Indirect costs before & during diagnosis	3
4.	Direct costs of patients during treatment	3
5.	Indirect costs during treatment	4
6.	Total costs of TB patients	5
7.	Productivity	5
8.	Coping costs	5
9.	Guardian costs	6
10.	Total healthcare costs (including HIV)	7
11.	Willingness and ability to pay	7
12.	Income and Affordability of health- and TB care	8
13.	Health Insurance	9
14.	Gender / social costs of TB	9
15.	Socioeconomic questions	10
Annex 1 : Income Indicator Usage and Income Data		12

back to index

1. Direct costs to patient before and during diagnosis

Measured indicator:
- direct costs before and during TB diagnosis
- the type of provider that was consulted
- the health seeking behavior of the patient

Questions:
- **Where did you go first?** *Check all that apply, includes current clinic*
- **Have you visited a traditional healer?**
- **How much did you spend for each of these visits?**

Information on the type of provider is useful for identification of the main consulted providers outside the public health sphere and can guide planning in respect to public private co-operations (PPM).

2. Patient and Health System Delays

Measured indicator:
- patient delay (time gap between onset of symptoms and first visit at any health facility)
- health system delay (time gap between first visit to facility and beginning of treatment)
- total delay (patient + health system delays)
- the type of provider consulted before patient reached program facility
- health seeking behavior of patient

Questions:
- **What symptoms did you experience that led you to seek treatment for your current illness? How long did you experience these symptoms before you went to seek treatment?**
- **Where did you go first?** *Check all that apply, includes current clinic*
- **Have you visited a traditional healer?**

These delays do not only increase the infectivity of a patient and lead to more serious illness by the time the patient presents him/herself, but also represent a time span in which additional costs are incurred. By addressing barriers and reasons for delay to timely diagnosis and treatment by the NTP, costs to TB patients, particularly among the poor, can be effectively reduced. For example, if the survey finds that patient delay is long, the NTP may choose to invest more in information and communication strategies to inform people about availability of health services and symptoms of TB. If on the other hand, the health system shows to be a delaying factor, the NTP will need to address issues such as decentralization of services, human resources, patient flow etc.

Onset of symptoms | **First visit to any facility** | **Diagnosis** | **Treatment begin**

Patient Delay

Health System Delay

Total Delay

back to index

3. Indirect Cost Before & During Diagnosis

Measured indicator:
- Indirect costs before and during TB diagnosis

Questions:
- **Have you ever stopped working/going to school/doing housework due to TB?**
- **What was your estimated personal take home earning per month BEFORE the TB illness?**
- **If you employed someone to do the housework for your household, how much would you have to pay him/her per day? While you are sick / While you are healthy**

The indirect costs are calculated by multiplying the time that the patient did not work with the average individual take home earning BEFORE TB or household replacement costs (see section on Income) .

4. Direct Costs of Patients During Treatment

Measured indicator:
- Direct costs during TB treatment
- Total direct costs due to TB
- Costs of hospitalization for TB patient

Questions:
- **How often do you travel to the health facility / hospital for taking your TB drugs?**
- **Where do you take your TB drugs?**
- **From your home to the facility, how much does it cost if you take transport?**
- **If you go to a facility, how much do you spend on food on that day**
- **Do you have to pay administration fees when picking up your TB drugs?**
- **Do you have any accommodation costs when picking up your TB drugs?**
- **Did you ever have to go to the health facility in addition to your regular visits for follow up tests since the beginning of treatment?** *If No, go to question xy*
- **If yes, did you have to pay any additional costs any time during the entire period?**
- **If so, what kind of costs and how much? If yes, how many times?**
- **Have you been hospitalized since you began your TB treatment?**
- **What do you estimate were the total costs of hospitalization?**
- **Do you buy any supplements for your diet because of the TB illness, for example vitamins, energy drinks, soft drinks, fruits or medicines?**
- **How much did you spend on these items in the last month approximately?**

The type of DOT, the treatment regimen (daily or three times weekly) and hospitalization may significantly affect direct (travel, food, fees) and indirect costs (time) to patients during treatment. The results of these questions should be therefore analyzed together with the results on direct costs before treatment by the NTP.

5. Indirect Costs During Treatment

Measured indicator:
- Indirect costs during TB treatment

Questions:
- **Have you been hospitalized at the beginning of your TB treatment?**
- **If yes, how many days in total did you stay at the hospital?**
- **Have you ever stopped working/going to school/doing housework due to TB?**
- **If yes, for how long?**
- **Does someone stay home specifically to take care of you? for how long?**

- **Did they quit their income-earning job to stay home and care for you?**
- **Did you have to change jobs when you became ill with TB?**

The time span in which the patient wasn't able to work is compared with the date of diagnosis and date of starting treatment. This determines the time span before treatment and during treatment during which the patient could not work.

- **If you employed someone to do the housework for your household, how much would you have to pay him/her per day? While you are sick / While you are healthy?**

This question measures the opportunity cost of a patient who is not paid for his/her work because of his/her work in the household. The aim of this question is not to assess whether or not the household now employs someone additional to do the housework since the onset of TB.

- **How long does it take you to get there (one way)**
- **How often do you travel to the health facility / hospital for taking your TB drugs?**
- **How long does one of these visits take on average, including time on the road**
- **How long does one of these follow-up visits take on average, including time on the road, waiting time and tests**
- **If yes, how many times did he/she go with you?**
- **how many days in total did you stay at the hospital?**
- **How many days did he/she stay with you (sleep there)?**
- **Did any other family/friend visit you while in hospital?**
- **If yes, how many people visited you?**
- **How many times did they visit you?**
- **How long were the visits including traveling time?**

Add the time spent on health facility DOT to the time spent on medicine collection and to the time spent on follow-up test visits and multiply this with the average personal take home earning that the patient earns NOW.

6. Total Costs of TB Patients

Measured indicator:

- Total direct costs of TB patients (prediagnostic, diagnostic, treatment)
- Total indirect costs of TB patients (prediagnostic, diagnostic, treatment)
- Total costs of TB patients (indirect + direct before diagnosis, during diagnosis, during treatment)
- Cost of TB on welfare of the household (willingness to pay)
- Cost of TB including pain and suffering (willingness to pay)

Total costs are the sum of:

- Direct costs before and during diagnosis
- Direct costs during treatment
- Indirect costs before and during diagnosis
- Indirect costs during treatment

Each subject is measured separately and the analysis of the data needs to put this information together again to obtain a full picture and relate the total costs then to income and food expenditure. See section on Income for details.

back to index

7. Productivity

Productivity means the ability of a person to work. When a person is healthy, he/she works for example 8 hours a day, and when he/she falls ill, he/she can only work 3 hours per day. This is a difference in productivity.

Measured Indicator:
- % reduction of productivity due to TB

Questions:
- **How many hours have you worked on average per week BEFORE you became ill with TB?**
- **How many hours do you work on average NOW per week?**
- **Is the change related to the TB illness?**
- **Is someone doing the work that you used to do?**
- **Is the reason for Not Working related to TB or any other illness?**
- **Have you ever stopped working/going to school/doing housework due to TB?**
- **If yes, for how long?**

8. Coping Costs

Coping costs of a household are costs to meet daily requirements despite extra expenditures or loss of income. These include the sale of assets, taking up debt, saving on food or other items (this is measured by the difference in food consumption before TB and during the treatment), taking a child out of school to care for the patient or taking up another job.

Measured Indicator:
- % of patients who take out loan
- costs due to interest on loan
- % of patients who sell assets
- Type of assets sold
- % reduction of household income spent on food due to TB
- Extent of reduction in food consumption
- % of patients whose children miss school to finance costs due to TB

Questions:
- **Do any of your children of or below school age work to finance costs due to the TB illness?**
- **Did you borrow money to cover costs due to the TB illness?**
- **Did you borrow any money to cover costs due to the TB illness?**
- **Have you sold any of your property to finance the cost of the TB illness?**
- **If YES: What did you sell?**
- **How much did you earn from the sale of your property?**
- **What is the estimated market value of the property you sold?**
- **How much food does your household consume NOW every month on average?**
- **Has the amount of food consumed per month changed due to the TB illness?**

back to index

9. Guardian Costs

A guardian is someone who accompanies the patient to the health facility/hospital or other visits because the patient cannot go by himself. Therefore, the guardian incurs also direct and indirect costs; as this is most likely another household member, the cost to the household is much higher if guardian costs are included.

Measured Indicator:

- Direct costs of guardians
- Indirect costs of guardians
- Total costs of guardians

Questions:

- **Does any family/friend/DOT supporter accompany you on these visits or go in your place to collect your TB drugs** ?
- **If yes, how many times did he/she go with you?**
- **How much does your friend/family/DOT supporter earn per day?**

- **Did any family/friend stay with you while in hospital?**
- If YES**: How many days did he/she stay with you (sleep there)?**
- **Were there any extra costs for your relative/friend for staying at the hospital?**
- **Did any other family/friend visit you while in hospital?**
- **If yes, how many people visited you?**
- **how many times did they visit you?**
- **How long were the visits including traveling time?**
- **How much does your friend/family normally earn per day?**

- **Does someone stay home specifically to take care of you?**
- If YES: **for how long?**
- **Did they quit their income-earning job to stay home and care for you?**

These are all guardian costs, which are costs incurred by the family due to the TB illness of one of the family members (or friend). The direct and indirect costs of hospitalization and treatment support for patients and their family can be assessed with these questions. The indirect cost is determined by the length of the visit (in hours) times forgone wage (per day) times the number of visits (assuming that each visit takes place at different days). Forgone wage can be then either calculated per hour or per half day lost.

The cost of transport for families to visit can be taken over from the transport item in the question *What do you estimate were the total costs of hospitalization?* The cost of transport needs to be multiplied by the answer given to question how often family visited. Note that for reasons of feasibility, costs of food for family members during visits to the hospital are not included.

Total Guardian direct costs:
(number of visits to hospital x cost of transport) +
(number of accompanied visits for treatment support x cost of visit to treatment supporter) +
(number of accompanied visits for medicine collection x cost of visit for medicine collection) +
(number of accompanied visits for follow up tests x cost of visit for follow up test)
+
Total Guardian indirect costs:
(Total time investment treatment support in hours / 8 x personal income per day) +
(number of days stayed in hospital x personal income per day) +
(number of visits to hospital x length of visit / 8 x personal income per day)

10. Additional Healthcare Costs (including due to HIV)

Measured Indicator:
- additional costs due to other illnesses

<u>Questions:</u>
- **Do you have any chronic illness for which you are receiving treatment?**
- If yes: **which?**
- If YES: **How much are these additional costs on average per month?**

It is desirable for a better comparison to elicit costs for any other wide-spread illnesses such as malaria, HIV/AIDS, or diarrhea. This allows us later to compare the TB costs to other illnesses costs. Depending on the program practice in the country, it may or may not be possible to ask patients their HIV status. Local privacy policies should be respected. If people are likely to say No when asked whether they are co-infected, although they are, it does not make sense to ask for additional costs. If it is not clear, asking about HIV status directly should be avoided, because patients might not be willing to say that they are infected or receive treatment. It will be difficult to discern costs due to an HIV co-infection or due to other illnesses. Since TB and HIV are closely connected, however, the likelihood that any additional illness is HIV is higher than for other illnesses.

Total monthly direct cost of healthcare is = (Total Direct cost TB / number of months between first help sought and time of interview) + average monthly costs due to other illnesses
The total monthly healthcare cost can be then compared to household income and personal income.

11. Willingness and Ability to Pay

Measured indicator:
- Cost of TB including pain and suffering

<u>Question:</u>
- **How much would you be willing to pay for not becoming ill with TB in the first place?**

- **How much did your household spend on FOOD every month on average BEFORE TB?**
- **How much does your household spend on FOOD NOW every month on average?**

- **Are there any additional costs for you because of this other illness <u>besides</u> the costs that you have already mentioned?**
- If YES: **How much are these additional costs on average per month?**

Willingness to pay incorporates the cost of pain and suffering, since people are expected to include them when evaluating how much they would pay to reduce their risk of illness or death. The cost of an illness on welfare of the household can be determined by the value the household would put on avoiding the disease.
Willingness to pay is not equal to ability to pay for the poor, because they might be willing but unable and therefore compensating by sacrificing on nutrition and other important items (see Coping costs).

The question does NOT mean what the patient can afford but - imagining unlimited income – how much he/she would be willing to pay, that is, how much <u>value</u> he/she associates with avoiding the disease. This number has to be then related to the information on personal income to see the differences in ability and willingness to pay.

back to index

12. Income and Affordability to TB Treatment and Healthcare

In order to estimate the impact costs have on a patient, we first need to know the amount that a patient can afford to spend on seeking and obtaining services. That is, we need to be able to judge what % of the patient's income is associated with costs of TB. Income is difficult to measure, but necessary for its relation to costs and consumption. Our primary goal is to see what the costs of TB patients are, whether or not they are too high and lastly, whether TB services are affordable for patients.

It is recommended that data income gathered through the survey be compared with findings of standardized measures obtained through household surveys or collected by UNDP, the World Bank, UNICEF, DHS or WHO (see Annex 1 on income indicator usage and income data).

Since income is difficult to measure, it is often preferred to measure consumption as a proxy for income. The best way is to distinguish between food and non-food items. This also lets us calculate the % of food costs of total costs, which also gives an idea about the social welfare of the household.

In this questionnaire, the question on non-food items is not included, because it takes time to estimate all costs that are incurred in a month on an item-by-item basis and it is heavily dependent on the type of expenditures that residents in a certain country have. If enough time is available, including a question on non-food expenditures with a list of items derived from the last Demographic and Health Survey should be considered.

Measured indicator:

- % of household income spent on food
- % of household income spent on TB pre-diagnostic and diagnostic costs
- % of household income spent on TB treatment
- % of household income spent on TB (pre-diagnostic, diagnostic, treatment)

- % of per capita income spent on TB pre-diagnostic and diagnostic costs
- % of per capita income spent on TB treatment
- % of per capita income spent on TB (pre-diagnostic, diagnostic, treatment)

- % of household income contributed by TB patient
- % reduction of household income due to TB illness of household member
- % reduction of personal income due to TB

- % of personal income spent on food

<u>Questions</u> (in addition to the ones on pre-diagnostic, diagnostic, treatment costs)

- **Do you have any chronic illness for which you are receiving treatment?**
- If yes: **which?**
- **Are there any additional costs for you because of this other illness <u>besides</u> the costs that you have already mentioned?**
- If YES: **How much are these additional costs on average per month?**
- **How many people live in your household?**
- **How many of the household members are paid for working?**
- **Besides yourself, does anyone else of your household receive treatment for TB?**
- **How much do you estimate is the average income of your household per month**
- **What was your estimated personal take home earning per month BEFORE your illness?**
- **What is your estimated personal take home earning per month NOW**
- **How much food did your household consume every month on average BEFORE the TB illness?**
- **How much food does your household consume NOW every month on average?**

back to index

13. Health Insurance

Insurance is essentially a transfer payment from the past to the future when need arises. Depending on the type of insurance, reimbursements need to be either deducted from patient's cost during treatment or insurance contributions need to be added to patients' costs. It is important to avoid double counting of expenditures for insurance payments and income for insurance reimbursements. Information on insurance coverage and the type of insurance reveals the degree of vulnerability of a patient and his/her family as well as whether they are part of a general social welfare system of private initiative, ie whether the welfare system captures the poor or not. If patients are subject to a reimbursement scheme, the reimbursement might be paid much later; until then, the costs that are to be reimbursed are real direct costs and should be counted as such.

Measured Indicator*:*
- % of patients covered by any kind of health insurance
- % of costs due to TB reimbursed by health insurance

Questions:
- **Do you have any kind of private or government health/medical insurance scheme?**
- If YES: **What type?**
- If YES: **Have you received reimbursement for any costs related to the TB illness?**
- If YES**: How much have you received as reimbursement?**

14. Gender / social costs of TB

The survey needs to be gender-sensitive, because evidence shows that women take longer to seek care due to stigma and social exclusion, heavier workloads, prioritization of other family members over own well-being, lack of independence, inaccessibility to financial resources and powerlessness in decision-making; they experience longer provider, diagnostic and treatment delays. Women are engaged in more activities that need to be replaced in the household, while girls replace these activities more than boys. In addition, women have higher direct costs than men, because they often need somebody to accompany them, they are less mobile and have less financial resources and women experience greater loss of income probably because of more lost work days.

Measured indicator:
- % of women who cannot seek care by themselves
- % of women who are financially independent
- % of TB patients whose private or social life was affected by TB
- Type of effect on private or social life
- % of patients where daughters don't attend school regularly due to TB case in family
- % of patients where daughters replaced work due to TB case in family
- Difference in direct costs between men and women
- Difference in indirect costs between men and women
- Difference in patient delays between men and women
- Difference in health system delays between men and women
- Difference in reduction of productivity due to TB between men and women
- Difference in reduction of personal income due to TB between men and women

Questions (in addition to the ones on direct, indirect, delay, productivity and income)
- **Why did someone accompany you?**
- **Are you financially independent?**
- **Is someone doing the work that you used to do?**
- **Do all of your children of school age attend school regularly?**

- **Do any of your children of or below school age work to finance costs due to the TB illness?**
- **Has the TB illness affected your social or private life in any way?**

15. Socioeconomic Questions

See also Guidelines on Socioeconomic Indicators

Measured Indicator:
- % of literate and illiterate patients
- Educational level of patients, head of household, spouse of head of household, primary income earner
- Level of impoverishment of household
- % of patients belonging to lowest income quartile/quintile of country
- % of patients belonging to poorest socioeconomic group
- % of patients belonging to minority (tribe/ethnic group/religion)

Questions:

- **What is the highest level of education for** ...? **Primary income earner? Head of household? Spouse of head of household?**
- **Are you currently formally employed?**
- **How are you usually paid?**
- **What is your main occupation?**
- **How many of the household members are paid for working?**
- **Do you own the house or residence you live in?**
- **Current place of residence?**
- **Do you own….**
- **How many rooms are there in your house?**
- **What type of toilet facility is available?**
- **What is your source of drinking water?**
- **What is your electricity supply?**
- ***What is your tribe / ethnic group / religion?***

Answers to socioeconomic questions can be given points in a scoring system for a total score in order to group respondents in different socioeconomic groups according to their score. It is not easy to devise a good scoring system and it should be avoided to just “invent” one. UNDP has used scoring systems and DHS as well. If a scoring system is used in the latest DHS, this could be taken and adapted to the questionnaire.
The UNDP Human Development reports split income data into quintiles. These quintiles should be compared to the data gained through the survey to group patients according to socioeconomic status.

Annex 1: Income Indicator Usage and Income Data

In order to estimate the impact costs have on a patient, we first need to know the amount that a patient can afford to spend on TB. We need to be able to judge what percentage of the patient's income is associated with costs of TB. There are two ways to approach this: either to ask patients through surveys and interviews about their income or consumption expenditures, or to use standardized measures of income, such as average wage rates, GNI per capita, and income levels. These standardized measures are usually obtained through household surveys or data supplied by UNDP, the World Bank[1], UNICEF[2], DHS[3] or the WHO[4]. However, these databases do not provide up-to-date income data on all countries.

For the purpose of developing a tool for NTP managers to estimate patient costs, both approaches face difficulties. The bottom-up approach requires substantial financial and human resources to conduct representative surveys. In the past, researchers have become more and more hesitant to use self-reported income data. Instead, they found data from household surveys more useful and representative.[5] The top-down approach is more practical, but average wage rates and GNI/capita don't provide the NTP with information specifically about the most vulnerable parts of the population targeted by the Tool. Top-down approaches only represent averages and therefore underestimate the financial burden on the poor.[6] A good and often-used alternative is recent data on household incomes obtained through country-level household surveys. Not every country has conducted such surveys, as they are expensive.[7]

Researchers have struggled with these problems and found different solutions. Filmer (2001) determined household assets in India to be sufficiently related to consumption expenditures to serve as a proxy for the latter. Hence, surveys on assets or consumption rather than income may serve the same purpose as surveys on income. Zhang et al (2007) used the indicator 'annual household medical expenditures during the last 12 months' as a proxy for estimating the costs for diagnosis and treatment. Fabricant et al (1999) used housing type, food expenditure and self-estimates as proxies for income levels in Sierra Leone and found that a one-day agricultural wage correlates with the average price of an out-patient visit in some countries and therefore serves as an indicator for affordable treatment.

Another difficult issue, and therefore often-times left out, is the method to estimate loss of income for individuals active in the household, but not in regular employment or waged activities. It is known, that in the short-run, activities are reallocated within the household.[8] In the long-run, however, they will need to be replaced. Drummond (1997) recommends either using the average wage, the cost of replacing the role, or the opportunity cost of production the individual could have contributed to if he/she was employed. These measures however run the risk of overestimation.

---

[1] Gwatkin et al 2007: Socio-economic differences in health, nutrition and population. World Bank.http://www1.worldbank.org/prem/poverty/health/ World Development Report 2006: Selected development indicators http://www-wds.worldbank.org/external/default/WDSContentServer/IW3P/IB/2005/09/20/000112742_20050920110826/additional/841401968_2005082630000823.pdf
[2] UNICEF Multiple Indicator Cluster Surveys: http://www.childinfo.org/MICS2/natlMICSrepz/MICSnatrep.htm
[3] Demographic and Health Survey DHS: http://www.measuredhs.com/countries/start.cfm
[4] WHO/World Health Surveys: www.who.int/healthinfo/survey/en/index.html
[5] Verbal communication with researchers from McGill and Liverpool School of Tropical Medicine
[6] Russell 1996
[7] It is argued that household surveys don't include the poorest of the poor, because many households in urban slums are not interviewed, and where it is considered to be risky or difficult to identify household entities (UN Research Institute for Social Development 2007)
[8] Drummond 1997

Income data

In trying to assess the impact of costs on the lowest income quartile or quintile of the population, the question remains which income measure to use. There is an extensive body of literature on measuring poverty which addresses the same question from different angles. There are three basic approaches:

1) Real measures:
   a. **National household budget surveys** – dependent on availability from national statistics office. Whether any surveys have been conducted recently can be seen by searching the International Household Survey Network (IHSN) database[9].
   b. **UNDP Human Development reports** [10] (detailed reports on national situations, distinguishing between urban and rural and by districts, giving information on real per capita expenditure in local currency, adjusted to Purchasing Power Parity).
   c. For Africa, the **Africa Development Indicators** 2006[11] provide recent detailed data.
   d. World Bank **Povcalnet data** by country on average monthly income, headcount of population living in poverty, Gini index[12] Compare this data with GNI/capita and poverty line of 1 US$ a day.
   e. **Living Standards Measurement Studies** [13] by the World Bank provide datasets of household surveys for many countries and guidelines for interpreting this data.
   f. **Gross national income per capita** for each country by World Bank[14]. If Gini coefficient (from Povcalnet) is low, GNI/capita can be used, don't use it with a high Gini. If % of population living below poverty line is small, GNI/capita can be used, otherwise don't use it.
   g. **Gross domestic product per capita** for each country by UN Statistics Division on social indicators[15]
   h. **ILO reports** on wages of unskilled/agricultural labor[16] per country

2) Absolute estimates:
   a. Absolute Poverty line: World Bank measures of absolute poverty: **1 US$ a day** (31 US$ per month) at purchasing power parity. This can be compared to GNI/capita and mean monthly income on Povcalnet. If they are similar, GNI/capita can be used. If they are very different, don't use GNI/capita.
   b. **Basket of goods** (minimum necessities): food vs. non-food items – dependent on availability from national statistics office or also in Human Development Reports

3) Relative estimates:
   a. Relative Poverty lines: These are usually set at **50-70% of median household income**[17]. GNI could be used as baseline as well. If Gini coefficient is low, this measure can be meaningful, not so with a high Gini.[18]

With all of these measures, the most recent and meaningful data should be taken:

Prioritization:

1) Recent (5 years or less old) **national household surveys** specifying income data according to geographical location or income quintiles of the population

---

[9] http://www.surveynetwork.org/home/?lvl1=activities&lvl2=catalog&lvl3=surveys
[10] http://hdr.undp.org/en/
[11] Household surveys p103ff http://siteresources.worldbank.org/INTSTATINAFR/Resources/ADI_2006_text.pdf
[12] http://iresearch.worldbank.org/PovcalNet/jsp/index.jsp
[13] http://www.worldbank.org/LSMS/
[14]http://web.worldbank.org/WBSITE/EXTERNAL/DATASTATISTICS/0,,contentMDK:20535285~menuPK:1390200~pagePK:64133150~piPK:64133175~theSitePK:239419,00.html
[15] http://unstats.un.org/unsd/demographic/products/socind/inc-eco.htm
[16] http://laborsta.ilo.org/
[17] Combat Poverty Agency 2006, OECD
[18] Cut off points for high and low Ginis could be (arbitrarily taken) at 20. Low Gini <20; high Gini >20

2) For Africa: the **Africa Development Indicators** 2006, for the rest of the world **Human Development Report** data
3) If none of the above are recent or available, compare GNI/capita, GDP/capita with World Bank poverty line and relative poverty line (60% of median or average household income), taking into account % of population living below poverty line and Gini coefficient. Make meaningful choice which one to use.
4) If available, take unskilled or agricultural wage from ILO database per country.

Example: Rwanda:

1) IHSN search yields no result.
2) Search on National institute of Statistics Rwanda website yields no result.
3) Search in Africa Development Indicators 2006 yields no result (country not listed)
4) Search on Human Development Report website yields following result: National Report Rwanda 2007
    - P. 15ff: Reaching the poor, p.19: average income in bottom quintile in 2006: Rwf18,900 /year
    - P. 20: average income of a poor person has remained virtually unchanged since 2001 at Rwf150 per day against Rwf146 per day in 2001.

back to index

Action points and possible interventions based on findings

Stage	Problem / Finding	Possible Actions & Interventions
Before and during diagnosis	Long patient delay	Improve accessibility through decentralizing diagnostic centers and/or integrating diagnostic services into general healthcare.<br>Engage other partners/providers in provision of TB services, e.g. Public-Private Mix activities.<br>Communication and awareness campaigns (targeting the poor) about TB symptoms and treatment and availability of services.<br>Conduct Knowledge, Attitudes and Practice Survey to generate baseline information in patient delay<br>Develop a TB health promotion plan<br>Identify whether particular minorities have long delay and investigate reasons.<br>Encourage demand for services by community
	Long health system delay	Training of healthcare staff in effective communication with patients<br>Investigate staff attitudes and practices<br>Assess patient flow at the health center<br>Consider a health service quality assurance system<br>Consider incentive system for staff such as for example possibility to attend training courses, performance-based salary payments<br>Develop feedback system between community and health services
	Women come late	Develop communication and advocacy activities geared towards women<br>Investigate whether this is due to staff attitudes, opening hours of facilities, intrinsic motivation, stigma, social or any other reasons<br>Cooperate with reproductive health services for referral system of symptomatic women<br>Cooperate with women's groups / NGOs
	Men come late	Investigate whether this is due to stigma, opening hours of facilities, accessibility, fear of losing job or any other reason.<br>Take diagnostic services where men primarily work<br>Start or engage with patient support group
	High direct diagnostic costs	Reduce unnecessary tests or unnecessary food supplements
	High accommodation costs	Organize overnight stay possibilities
	High transport costs	Organize transport of sputum samples or slides from remote areas to laboratories<br>Make diagnostic facilities easily accessible
	High user fees	Abolish user fees for TB patients<br>Consider introduction of health insurance scheme<br>Reimburse user fees
	High indirect costs	Consider cooperation with social welfare programs, community funds, micro health insurance etc.

back to index

Action points and possible interventions based on findings

Stage	Problem / Finding	Possible Interventions
During treatment	High transport costs	Integrate TB services into public health workplace interventions
	High informal payments	Provide TB drugs free of charge in public services<br>Discourage under the table payments through measures to increase staff motivation, monitoring or punitive measures<br>Let patients evaluate service provision<br>Reduce unnecessary drugs, tests and food supplements
	High food costs	Provide food package, food vouchers<br>Cooperate with partners such as World Food Program or charities
	High accommodation costs	Provide possibility for overnight stay
	High guardian costs	Health insurance<br>Provide transport vouchers for patient and guardian
	High costs for DOT visits<br>High costs for medicine collection<br>Long time on the road	Introduce community DOT<br>Provide transport vouchers<br>Provide transport<br>Decentralize services
	high costs for follow up tests	Provide transport vouchers or transport<br>Reduce/eliminate fees for tests<br>Provide food package
	High costs for hospitalization<br>Long hospitalization periods	Hospitalization of only very seriously ill cases<br>Reduce hospitalization to minimum (1 to 2 months max)<br>Provide food, sheets, medicines, tests for free
	High costs for 'special foods'	Sensitize public health staff to inform patient about unnecessary special foods
	High coping costs due to loans	Health Insurance, micro insurance
	Patients save on food	Provide food package<br>Health insurance
	High costs for non-TB medical care	Cooperate with HIV program for more integrated services
Labor Costs	Many patients lose their jobs	Discuss/cooperate/advocate with labor organizations, employers, politicians and consider legal framework to protect TB patients<br>Organize workplace programs

Reference: WHO (2005*). Addressing Poverty in TB Control*. http://whqlibdoc.who.int/hq/2005/WHO_HTM_TB_2005.352.pdf

Separated printfiles

1 Introduction

2 Literature Review

3 Review SE indicators

4 List of indicators to be measured

5 Questionnaire

6 Guidelines adaptation

7 Guidelines methods sampling interviewer training

8 Guidelines interpretation of results

9 Guidelines interventions
